# From Bench to Bedside: The Role of Extracellular Vesicles in Cartilage Injury Treatment

**DOI:** 10.34133/bmr.0110

**Published:** 2024-11-22

**Authors:** Pan Jin, Huan Liu, Xichi Chen, Wei Liu, Tongmeng Jiang

**Affiliations:** ^1^ Health Science Center, Yangtze University, Jingzhou 434023, Hubei, China.; ^2^ Key Laboratory of Emergency and Trauma of Ministry of Education, Key Laboratory of Haikou Trauma, Key Laboratory of Hainan Trauma and Disaster Rescue, The First Affiliated Hospital of Hainan Medical University, Hainan Medical University, Haikou 571199, China.; ^3^ Engineering Research Center for Hainan Bio-Smart Materials and Bio-Medical Devices, Key Laboratory of Hainan Functional Materials and Molecular Imaging, College of Emergency and Trauma; Hainan Provincial Stem Cell Research Institute; Hainan Academy of Medical Sciences, Hainan Medical University, Haikou 571199, China.

## Abstract

Cartilage repair is the key to the treatment of joint-related injury. However, because cartilage lacks vessels and nerves, its self-repair ability is extremely low. Extracellular vesicles (EVs) are bilayer nanovesicles with membranes mainly composed of ceramides, cholesterol, phosphoglycerides, and long-chain free fatty acids, containing DNA, RNA, and proteins (such as integrins and enzymes). For mediating intercellular communication and regulating mechanisms, EVs have been shown by multiple studies to be effective treatment options for cartilage repair. This review summarizes recent findings of different sources (mammals, plants, and bacteria) and uses of EVs in cartilage repair, mechanisms of EVs captured by injured chondrocytes, and quantification and storage of EVs, which may provide scientific guidance for promoting the development of EVs in the field of cartilage injury treatment.

## Introduction

Osteoarthritis (OA) is a degenerative joint disease characterized by articular cartilage degradation, synovial inflammation, and subchondral bone remodeling [1]. Many factors contribute to the development of OA, such as age, obesity, joint trauma, genetic predisposition, and low-level inflammation. Although the pathogenesis of OA is still being explored and is not fully understood, articular cartilage injury is the key to OA. The incidence of OA has doubled in the last 3 decades, currently affecting more than 500 million people, and the incidence of the disease will continue to increase in the coming decades, which may be related to the aging population and obesity epidemic [2–4]. At present, there are limited means to prevent the progression of OA. Existing drugs can only relieve symptoms, with little effect on the repair and regeneration of damaged cartilage, and some drugs have serious side effects [5,6]. When the disease progresses to the end stage, joint replacement becomes the only treatment [7,8]. However, artificial joint replacement also has certain disadvantages, such as the longevity of the implant, infection caused by the implant, and the possible need for a second operation [9,10]. Therefore, new treatments that can help delay the progression of the disease are urgently needed.

Articular cartilage is a special connective tissue on the surface of joints and acts as a shock absorber, buffer, and lubricant. It is a blood-free tissue without blood vessels or nerves, and it relies mainly on synovial fluid in the joint cavity for nutrients and oxygen [11]. This lack of blood supply underlies the poor recovery ability of articular cartilage, and therefore, the repair and regeneration of damaged articular cartilage have been a challenge. Cartilage damage is usually caused by trauma, prolonged weight bearing, degeneration, and eventually OA [12]. The main components of articular cartilage are chondrocytes and extracellular matrix (ECM). Chondrocytes are the only cell type in articular cartilage, and chondrocyte hypertrophy, aging, and death are important factors in the occurrence of many joint diseases [13]. The ECM is mainly composed of type II collagen and proteoglycan, which together maintain the physiological functions of chondrocytes, which is reckoned as a hallmark of OA. Cartilage damage occurs when the degradation rate of ECM exceeds that of synthesis [14]. In recent years, extracellular vesicles (EVs) have become the focus of research for mediating the repair and regeneration of cartilage [15–18].

**Fig. 1.**
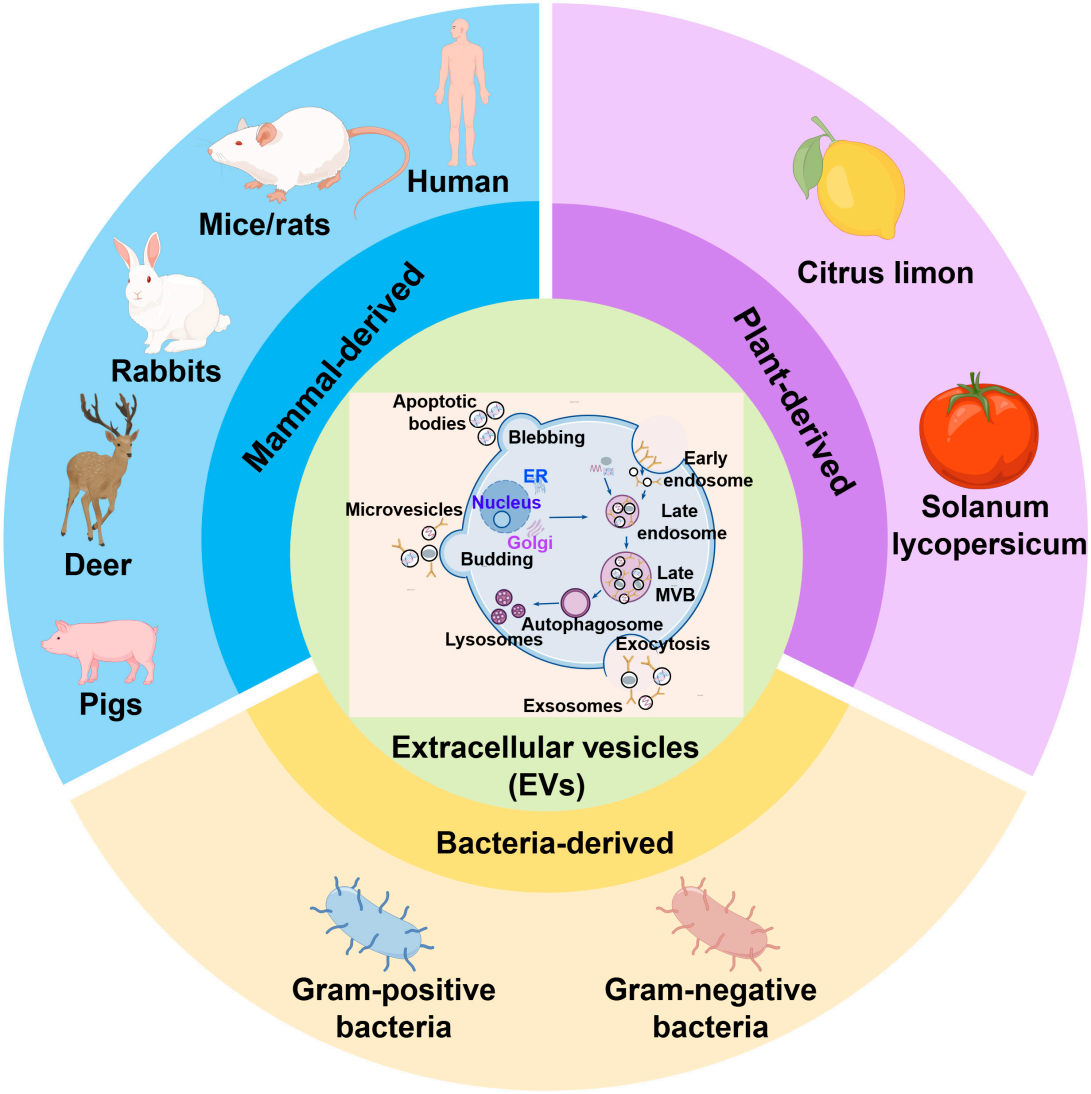
EVs of typical sources (mammals, plants, and bacteria) serve as effective treatment options for cartilage repair. By Figdraw.

**Fig. 2.**
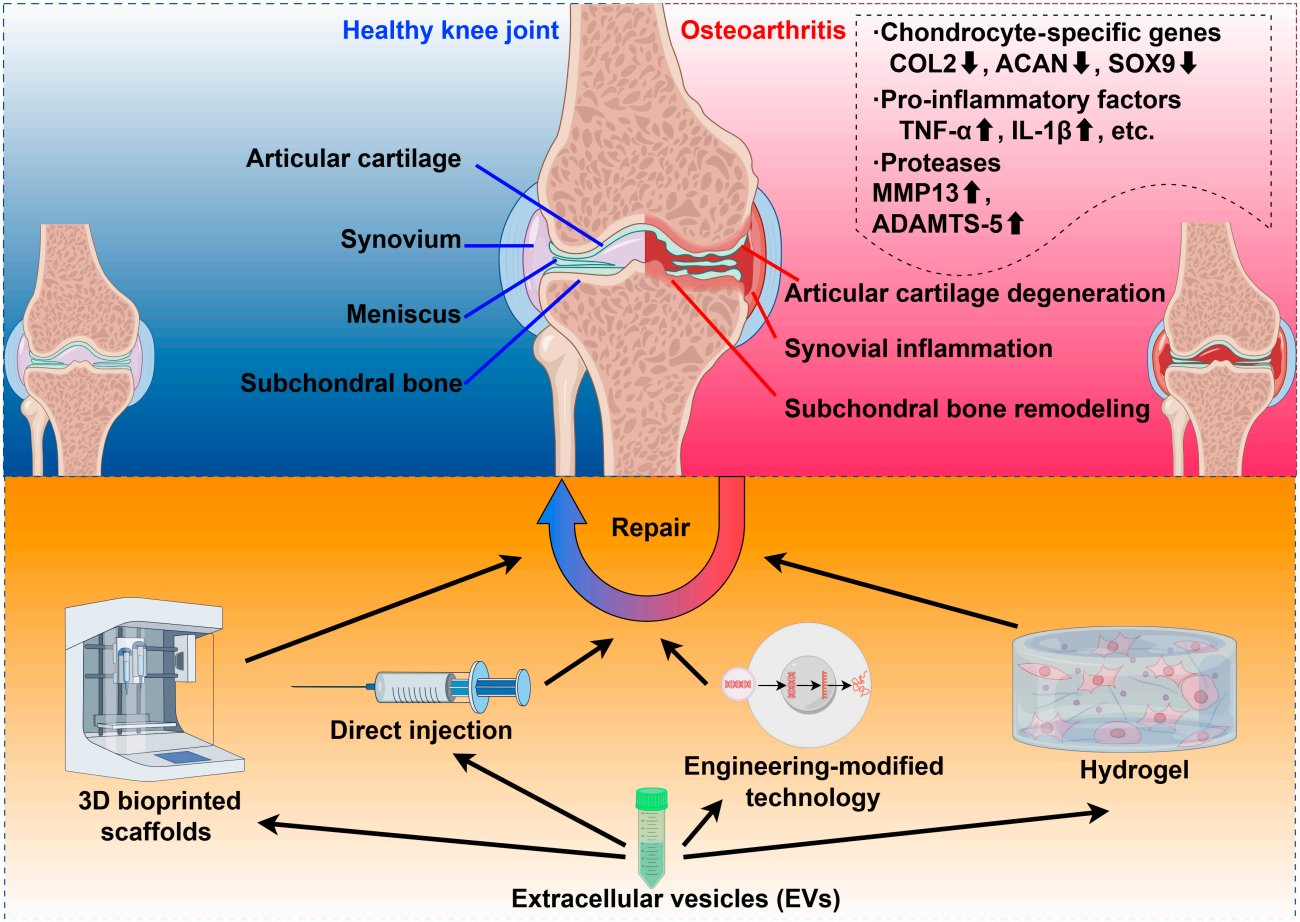
Different methods (direct injection, hydrogels, engineering-modified technology, and 3D bioprinted scaffolds) were adopted for EV delivery, thus enhancing cartilage repair. By Figdraw.

EVs are membrane vesicles of different origins and are mainly divided into 3 categories: exosomes, microvesicles, and apoptotic bodies. Exosomes are tiny vesicles (30 to 150 nm in diameter) secreted by the plasma membrane that are characterized by invagination and inward budding, forming a distinctive cup-like structure. Microvesicles or ectosomes (100 to 1000 nm) are formed by direct budding of cell membranes, while apoptotic bodies (~500 nm) are produced by the plasma membrane of apoptotic cells and undergo contraction and fragmentation [15,16]. As endocytosis carries vesicles, early endosomes develop into multivesicular bodies through the action of a series of intracellular biological and chemical factors [19]. Under the control of a series of endolysosomal systems including endosomal sorting complexes required for transport (ESCRT) machinery and Syndecan-syntenin-ALIX, exosomes are formed in the plasma membrane by budding [20]. Microvesicles or ectosomes arise from multiple biogenesis pathways, such as releasing large oncosomes from bladder cancer cells, forming smaller “classical” ectosomes on the surface of colorectal cancer cells, secreting more smaller ectosomes including ARMMs (ARRDC1-mediated microvesicles) through arrestin domain-containing protein 1 (ARRDC1)-dependent pathway, and budding off from the tip or side of the protruding surface. Most studies suggest that apoptotic bodies do not play a therapeutic role in cartilage injury, but recent research revealed that apoptotic bodies can inhibit cartilage damage and ameliorate abnormal gait via reversing the imbalance of M1/M2 macrophages [21]. EVs perform a variety of functions in biological systems, but it is difficult to obtain very pure and single EV samples by the current methods of EV separation for extracellular particles that are heterogeneous with undefined biogenesis origin (Fig. S1A) [22]. At present, the extraction methods of EVs reported in the literature include but are not limited to 7 methods as shown in Fig. S1B [22]. Up to now, no single extraction method can be used to extract all EVs, and more research is needed to optimize the extraction process of EVs from different sources and with different requirements. Because of the lack of specific markers, the identification of EVs is also a difficult problem to be solved. According to the guidance manual “MISEV 2018” published by the International Extracellular Vesicle Association, the characterization of EVs was verified by nanoparticle tracking analysis (NTA) measurement of particle size distribution, transmission electron microscopy (TEM) analysis of morphology, and Western blot (WB) detection of marker proteins. The particle size distribution and particle concentration of EVs were analyzed by NTA, the morphological characteristics of EVs were observed by TEM, and EV marker proteins were detected by WB [23]. Innovative fluorescence labeling and microchips including surface-enhanced Raman scattering (SERS)-multichannel platform utilized for visualizing EVs are state of the art nowadays [24,25]. MISEV2023 recommends the continuation of EV characterization using the 5 classes of marker proteins in MISEV 2018, but suggests that affinity-based enrichment schemes involving the quad-trans proteins CD9, CD63, and CD81 are not specific to exosomes, and not all EVs will display these proteins. Meanwhile, the research guideline recommended orthogonal methods for EV characterization, because they are unlikely to have the same biases [22]. EVs are widely found in the human body, animals, plants, and bacteria (Fig. 1) and are secreted by both prokaryotic and eukaryotic cells [26]. EVs have been isolated from a variety of body fluids, including blood, urine, saliva, breast milk, amniotic fluid, ascites, cerebrospinal fluid, bile, and semen [27]. EVs can promote cell proliferation, regulate immunity, promote angiogenesis and tissue repair, and have great potential in the treatment of cartilage regeneration [17,28]. Except for direct injection, cutting-edge technologies including combining with hydrogels [29], 3-dimensional (3D) bioprinted scaffolds [30], and engineering-modified strategies [31,32] are also adopted for EV delivery (Fig. 2).

## Application of EVs of Different Sources in Cartilage Repair

### Mammals

#### EVs derived from human

EVs are important mediators for transmitting information between cells and for regulating physiological functions. EVs derived from mesenchymal stem cells (MSCs) have been reported to have cartilage regeneration potential, immune properties, and protective effects on cartilage [33]. At present, the efficacy of MSC therapy is mainly because EVs derived from MSCs can deliver specific substances to recipient cells [34,35]. Furthermore, it is noteworthy that the Chinese Research Hospital Association introduced guidelines pertaining to human MSC-derived small EVs (hMSC-sEVs) on 2021 December 28, with these guidelines being enforced as of 2022 January 1. The standard elaborates on the technical specifications for hMSC-sEVs, encompassing the criteria for test methods and regulations, packaging, user instructions, labeling, storage, transportation, and waste management procedures [36].

EVs of human umbilical cord MSCs: Human umbilical cord MSCs (hUCMSCs) are pluripotent stem cells derived from neonatal umbilical cord mesenchymal tissues. Studies on hUCMSCs demonstrate their strong proliferation and differentiation ability and high immunomodulatory activity without ethical issues [37]. hUCMSC-EVs are reported to have several advantages and characteristics, including ease of extraction and amplification, low risk, and biological characteristics due to their origin from hUCMSCs [37]. In addition, hUCMSC-EVs also have anti-inflammatory effects and protect cartilage by interacting with METTL3 to reduce m6A levels of NLRP3 in macrophages, thereby up-regulating ECM proteins (COL2A1 and Aggrecan) and down-regulating ECM protein-degrading enzymes (ADAMTS5 and MMP13) [38]. Li et al. [39] found that hUCMSC exosomes (hUCMSC-Exos) alleviated OA by regulating the polarization of macrophages, and in vitro experiments also found that exosomes reversed the damage to OA-like chondrocytes in vitro caused by IL-1β. Other researchers also found that hUCMSC-EVs promoted the polarization of M2-type macrophages and the expression of anti-inflammatory factors and inhibited the level of inflammation-related factors in OA chondrocytes caused by IL-1β, thus exerting an immunomodulatory effect and alleviating the cartilage degeneration of OA [40]. In addition, UCMSC-Exos inhibited the p53 signaling pathway, decreased the expression of the OA cartilage aging gene, and improved the vitality of OA cartilage cells. It also promoted the secretion of hyaline cartilage matrix and collagen II (COL II), allowing its cartilage to regenerate [41]. Wang et al. [42] found that hUCMSC-Exos had a reverse effect on the expression of genes and proteins related to inflammation in chondrocytes. hUCMSC-Exos reduced apoptosis of chondrocytes and promoted the proliferation and differentiation of chondrocytes. Studies have shown that hUCMSC-EVs can maintain chondrocyte homeostasis and alleviate OA pathology. The key mechanism behind this therapeutic effect is the regulation of the NOD-like receptor family, pyrin domain containing 3 (NLRP3) pathway and pyrodeath by miR-223. miR-223 directly binds to the 3′ untranslated region (3′UTR) of NLRP3 mRNA and inhibits activation of the NLRP3 pathway, thus reducing the inflammatory response and apoptosis of chondrocytes and promoting cartilage repair and recovery of joint function [43]. Hu et al. [29] used a combination of gelatin methacrylate (GelMA) and nano-clay hydrogel (Gel-nano) to transport hUCMSC-EVs. In vivo studies showed that the Gel-nano hydrogel had good biocompatibility and that it promoted a sustained release of EVs (up to 31 days), thus promoting long-term regeneration of cartilage. Moreover, due to the addition of nano-clay to the GelMA hydrogel, the mechanical and biological properties were improved. Sun et al. [30] designed a 3D-printed double-layer porous hydrogel scaffold with different moduli and compositions in upper and lower layers. Black phosphorus (BP) and hUCMSC-Exos were added in GelMA to fabricate the upper layer. In addition to BP and hUCMSC-Exos, β-tricalcium phosphate (β-TCP) with osteoconductive and osteoinductive effects was added to GelMA to manufacture the lower layer. In vivo and in vitro experiments showed that the hydrogel scaffold promoted the differentiation and repair of cartilage tissues. Exosomes were released continuously for 28 days, and the final release rate reached 92%.

EVs of human embryonic MSCs: It has been reported that exosomes derived from embryonic stem cells promote the dynamic remodeling of cartilage and accelerate the repair of cartilage defects. In addition, they can promote the proliferation of proliferating cells [proliferative cell nuclear antigen (PCNA)], reduce apoptosis of apoptotic cells [cleaved caspase-3 (CCP3)], and inhibit the production of inflammatory cytokines [44]. Zhang et al. [45] created cartilage defects on the trochlear groove of the distal femur of rats and treated them with human embryonic MSC-derived exosomes and phosphate-buffered saline (PBS). The results showed that after 12 weeks of treatment, the cartilage defect was completely repaired in the exosome treatment group, while the PBS treatment group only had fibrous repair tissue. Wang et al. [46] found that human embryonic MSC-derived exosomes maintained a chondrocyte phenotype, increased COL II synthesis, and decreased ADAMTS5 expression, thereby reducing cartilage destruction and matrix degradation in a destabilization of the medial meniscus (DMM) mouse model of OA. Other studies have confirmed that the application of exosomes derived from human embryonic MSCs in temporomandibular joint OA inhibited inflammation, restored stromal homeostasis, promoted cell proliferation, and reduced cell apoptosis, thus repairing temporomandibular joint damage [47]. In addition, another study has shown that multiple injections of full-term human placenta tissue-derived exosomes (pExos) relieved OA pain, repaired cartilage damage, and reduced the expression of proteins associated with inflammation, degradation, and apoptosis in vivo; furthermore, pExos promoted chondrocyte migration and proliferation by regulating cell growth-related signaling pathways [such as the AKT and extracellular signal–regulated kinase (ERK) signaling pathways] and inhibited inflammation and the process of cartilage degradation in vitro [48]. To compare the efficacy of MSC exosomes with hyaluronic acid (HA) against HA alone for functional cartilage regeneration in a rabbit osteochondral defect model, Wong et al. [49] isolated MSC exosomes from a clonal immortalized E1-MYC 16.3 human embryonic stem cell-derived MSC line and created critical-size osteochondral defects (4.5-mm diameter and 1.5-mm depth) on the trochlear grooves in the knees of 18 rabbits. After randomly allocating to 2 treatment groups, three 1-ml injections of either exosomes and HA or HA alone were administered intraarticularly immediately after surgery and thereafter at 7 and 14 days after surgery. At 6 and 12 weeks, gross evaluation, histologic and immunohistochemical analysis, and scoring were performed. The results showed that the combination of MSC exosomes and HA exhibited a superior cartilage reparative response when compared with HA alone. After confirming the effectiveness of exosomes in rabbits, Zhang et al. [50] created bilateral osteochondral defects (6-mm diameter and 1-mm depth) in the medial femoral condyles in knees of 12 micropigs, and exosomes from immortalized E1-MYC 16.3 human embryonic stem cells and HA (exosome + HA) or HA alone were injected intraarticularly after surgery and thereafter at 8 and 15 days. Magnetic resonance imaging (MRI) at 15 days, 2 months, and 4 months and macroscopic, histological, biomechanical, and micro-computed tomography (μCT) analyses at 4 months indicated that the combination of MSC exosomes promotes functional cartilage and subchondral bone repair. However, using exosomes of the same stem cell origin, the work of another group came to the opposite conclusion. Hede et al. [51] created 2 chondral defects (ø = 6 mm) in the trochlea of each knee in Göttingen minipigs: one defect was made in the distal, medial trochlea, and another was made in the lateral trochlea, 0.5 to 1 cm proximal to the first defect. The pig was then randomized to receive intraarticular injections of either 1 mg of MSC-EVs in 1 ml of PBS or 1 ml of PBS at 2 and 4 weeks postoperatively. Ultrasound-guided injections were performed at 2 and 4 weeks, and gross examination, histology, histomorphometry, immunohistochemistry, and μCT analysis were evaluated at 6 months after surgery. In vivo experiments revealed that intraarticular injections of MSC-EVs were effective in enhancing healing of subchondral bone instead of cartilage and MSC-EVs impaired optimal cartilage repair.

EVs of human synovial MSCs: Synovial MSCs (SMSCs) are pluripotent stem cells derived from the synovial tissue that have characteristics of self-renewal and multidirectional differentiation. The synovial membrane is a part of the joint intima, in which the mesenchymal cells have the characteristics of stem cells and can differentiate into osteocytes, chondrocytes, adipocytes, and other cell types [52]. EVs derived from lipopolysaccharide (LPS)-preconditioned SMSCs were reported to inhibit ECM degradation and alleviate knee OA [53]. It was found that modification of SMSC-Exos through overexpression of miR-140-5p compensated for a deficiency of cartilage ECM and promoted the regeneration of cartilage tissue [54]. Wang et al. [55] found that SMSC-Exos promoted the proliferation and migration of chondrocytes but had no effect on ECM secretion. They overexpressed miR-155-5p, and the results showed that after SMSC-miR-155-5p-Exo treatment, the secretion of ECM-related proteins CoL II and SOX9 was 40 times that after SMSC-Exo treatment. Qiu et al. [56] reported that exosomes rich in miR-129-5p human SMSCs alleviated IL-1β-induced inflammatory response and apoptosis of chondrocytes by targeting HMGB1. In another study, exosomes released by human SMSCs overexpressing miR-212-5p (SMSC-212-5p-Exos) showed that SMSC-212-5p-Exos inhibited up-regulation of IL-1β-induced E74-like ETS transcription factor 3 (ELF3), reduced the expression of degenerate molecules and matrix metalloproteinases, and reduced the expression of inflammatory molecules [57]. Another study injected SMSC-derived exosomes directly into the joint cavity once a week, 5 to 8 weeks after DMM. The results showed that the exosomes inhibited degradation of the ECM and apoptosis of chondrocytes and promoted repair of cartilage injury [58]. In the process of investigating the effects of exosomes derived from synovial fluid-derived cells (SFDCs) cultured under normoxic conditions in a 2D monolayer or encapsulated within a 3D matrix for chondrogenic differentiation in vitro and cartilage defect repair in vivo, Han et al. [59] found that 3D-cultured exosomes exhibited better healing properties and chondrogenic markers including CD63, CD81, and CD9 exhibited higher expression in the 3D culture compared to exosomes derived from a 2D culture. This study implies that different culture regimens affect the surface protein expression of EVs, and the relevant mechanism needs further study to elucidate.

EVs of human bone marrow MSCs: EVs derived from human bone marrow MSCs (BMMSCs or BMSCs) could reduce inflammatory responses in OA chondrocytes, including pro-inflammatory cytokines and collagenase activity. BMMSC-EVs also inhibited activation of the nuclear factor κB (NF-κB) signaling pathway, which plays an important role in OA pathology. The study also found that adding BMMSC-EVs to an OA chondrocyte culture promoted the production of proteoglycan and type II collagen and restored and maintained the homeostasis of cartilage [60]. Mao et al. [61] established a mouse model of OA induced by collagenase and found that human BMSC-miR-92a-3p-Exos injected into the joint cavity substantially inhibited the degradation function of chondrocytes and reduced the degradation of cartilage. Further investigation revealed that the main reason was the inhibition of the expression of WNT5A, a signaling pathway protein related to cartilage formation and degradation. Zhou et al. [62] isolated pBMSC-Exos from tissue specimens surgically removed from children with polydactylism and injected the exosomes into joint cavities. This significantly improved a knee cartilage injury of an OA mouse model induced by collagenase, and the OARSI score was significantly reduced. In addition, they compared pBMSC-Exos with BMSC-Exos and found that pBMSC-Exos had better efficacy. It has been reported that hBMSC-EVs promote the proliferation and migration of OA chondrocytes and reduce cell apoptosis. They have also been shown to reverse the down-regulated expression of chondrocyte degradation genes under IL-1β stimulation and reduce the phosphorylation level of inflammation-related signaling pathways [63]. Wang et al. [64] also reported that exosomes derived from human BMMSCs significantly increased the survival of IL-1β-induced chondrocytes and inhibited their apoptosis. Liao et al. [65] established a rat model of OA by anterior cruciate ligament transection (ACLT) and medial meniscectomy (MMx) and isolated exosomes from BMMSCs. They injected exosomes into the joint cavity twice a week for 4 weeks and found that the method alleviated OA inflammation and promoted cartilage regeneration. In addition, Yang et al. [66] prepared hBMSC-Exos, and the experimental results showed that hBMSC-Exos promoted the repair of cartilage defects of the patellar groove in rabbits. Another study, using an injectable silk fibroin (SF) hydrogel containing articular chondrocytes and hypoxia preconditioned exosomes, showed in vivo that the hydrogel system sustainably released exosomes for 31 days and effectively promoted cartilage regeneration in a rat model of cartilage deficiency [67]. Considering these problems including a low yield of primary cells, long time of in vitro expansion, and diminished differentiation capability after passaging by extracting BMSCs directly from bone marrow, Deng et al. [68] used recombinant human bone morphogenetic protein 2 (BMP-2)-loaded gelatin sponge scaffolds to construct in vivo osteo-organoids. They suggest that the in vivo osteo-organoid-derived MSCs exhibited stronger anti-replicative senescence capacity during passage and amplification, and can expand more high-purity MSCs in a shorter time (6 days versus 12 days for obtaining passage 1 MSCs) while retaining stronger stem cell properties when compared with traditional extraction methods. However, there is no comparative study to evaluate the repair effect of EVs from the in vivo osteo-organoid-derived MSCs and exosomes from BMSCs on cartilage injury.

EVs of human adipose MSCs: Adipose tissue is mainly derived from liposuction, and the cells obtained are not subject to the ethical concerns of other stem cell sources, such as embryonic stem cells. Adipose tissue is a viable alternative to bone marrow because of its abundant sources and low incidence of adverse sequelae from the donor [69,70]. EVs derived from adipose-derived mesenchymal stem cells (ADSCs) may protect the cartilage from damage by regulating autophagy. One study showed that microvesicles and exosomes isolated from an ADSC-conditioned medium inhibited IL-1β-induced oxidative stress in OA chondrocytes, and the effect of microvesicles was greater than that of exosomes. In addition, they also enhanced the expression of autophagy markers in OA chondrocytes and exerted anti-inflammatory and anti-catabolic effects for treating cartilage degradation [71]. Wu et al. [72] reported that MSC-derived exosomes with miR-100-5p up-regulated autophagy by inhibiting the mTOR pathway, thus reducing damage to cartilage from IL-1β, enhancing anabolism, and inhibiting catabolism. In addition, exosomes extracted from subcutaneous fat stromal cells inhibited the expression of mTOR to induce chondrocyte autophagy and promote cartilage repair (Fig. S2) [72]. Microvesicles were also shown to reduce the activity of matrix metalloproteinases and the expression of MMP-13 while enhancing the expression of COL II to protect cartilage [73]. Studies have shown that hADSC-EVs inhibited the expression of MMP-13 and ADAMTS5, increased the expression of type II collagen, reduced the degradation of cartilage matrix, and promoted the repair of damaged cartilage [74]. In addition, ADSC-derived EVs used to treat OA chondrocytes were found to reduce the production of inflammatory mediators, reduce the inflammatory response, and enhance the production of anti-inflammatory cytokines. Cavallo et al. [75] found that ADSC-derived EVs inhibited activation of the NF-κB signaling pathway, down-regulated ADAMTS5, and counteracted IL-1β-induced inflammatory effects. Li et al. [76] reported that miR-376c-3p in hADSC-Exos regulated the WNT–β-catenin signaling pathway by inhibiting the expression of WNT3 and WNT9a, alleviating chondrocyte degradation and synovial fibrosis. Another group used tissue-specific acellular ECM and human adipose MSC-derived exosomes to create a dual-network hydrogel scaffold with biomimetic properties through 3D printing. The expression levels of cartilage-specific genes, including an aggregation protein (ACAN), gel prototype (COL II), and SRY-box transcription factor 9 (SOX9), were significantly up-regulated in preclinical rat models by the 3D-printed tissue-specific hetero-bilayer scaffold and promoted cartilage regeneration [77].

EVs of human MSCs from other sources: EVs can also be extracted from stem cells from other sources and have been shown to play an important role in cartilage repair. Liu et al. [78] also used this method to establish a rat OA model, and their intraarticular injection of human urine stem cell-derived exosomes at 4 to 8 weeks after surgery also promoted cartilage regeneration and subchondral bone remodeling. Other scientists report a method of encapsulating stem cell-derived exosomes (SC-Exos) into photo-induced imide cross-linked hydrogels to prepare SC-Exo composite hydrogel (EHG) tissue patches. The results of an in vivo study showed that EHGs promoted the repair and regeneration of a rabbit patellar bone cartilage defect [79]. In addition, Yang et al. [80] developed an injectable Diels–Alder crosslinked HA/polyethylene glycol (PEG) hydrogel (DAHP) for controlled release of small stem cell-derived EVs (MSC-sEVs) to improve OA. In in vivo studies, they established a rat OA model by ACLT and partial MMx (pMMx) and injected iMSC-sEVs loaded in a DAHP hydrogel into the joint cavity 1 week later. The results showed that the DAHP hydrogel-controlled release group had a similar effect on improving OA as the multiple injection group.

EVs of human chondrocytes: As the resident cells of articular cartilage, EVs of human chondrocytes are easy to be reckoned as therapeutic agents in OA. Liang et al. [81] fabricated chondrocyte-affinity peptide (CAP) surface engineering of human knee cartilage-derived exosomes for targeted delivery of miR-140. Their results demonstrated that CAP-exosomes can specifically bind to chondrocytes, deliver miR-140 in vitro, and rejuvenate OA cartilage by suppressing matrix-degrading enzymes in vivo.

EVs of other human tissues: When the inner wall of the blood vessel is damaged, platelets are activated and gathered at the injured site, releasing a large number of tissue repair factors to promote the repair of the injured area. As a highly concentrated platelet product with characteristics of safety, easy access, and affordable cost, platelet-rich plasma (PRP) participates in tissue regeneration of many domains [82]. Zhang et al. [83] investigated the inclusion of human PRP-derived exosomes in a poloxamer 407 and 188 mixture-based thermoresponsive hydrogel and found that the PRP-Exo incorporated Gel (Exo-Gel) alleviated cartilage damage in a mouse model of subtalar OA and that the Exo-Gel sustained exosome release in vivo for up to 28 days.

#### EVs derived from mice

To evaluate the immunomodulatory function in arthritis, Cosenza et al. [84] isolated exosomes from BMMSCs of C57BL/6 mice and investigated the immunosuppressive effects on T and B lymphocytes in vitro and in the delayed-type hypersensitivity (DTH) and collagen-induced arthritis (CIA) models. Their results show that MSC-derived exosomes exerted a more effective anti-inflammatory role in suppressing inflammation in vitro and in vivo. Exosomes with miR-206 derived from mouse BMMSCs decreased the apoptosis index of OA mice by negatively regulating Elf3, thus promoting the proliferation and differentiation of osteoblasts and alleviating OA in mice [85]. Shen et al. [86] found that BMSC-Exos in mice increased the expression of TUC339, decreased the percentage of M1-type macrophages, and increased the percentage of M2-type macrophages, with consistent expression of polarization-labeled proteins. In vivo experiments confirmed that mice BMSC-Exos inhibited inflammation and promoted the activity of chondrocytes, thereby improving the pathological status of OA. Pang et al. [87] used a GelMA hydrogel to encapsulate nanovesicles derived from MSCs (GELMA-NVs) and found that GELMA-NVs promoted chondrocyte catabolism and inhibited chondrocyte synthesis in a DMM-OA model. In addition, they significantly polarized macrophages toward the M2 phenotype and inhibited the secretion of pro-inflammatory cytokines, thus reducing the severity of cartilage injury. The sustained release time of nanovesicles derived from the MSCs was up to 30 days. Zeng et al. [88] also reported a mussel-inspired multifunctional hydrogel system loaded with exosomes and icariin, which achieved a synergistic effect of the 2 components. It slowed cartilage degeneration, promoted cartilage regeneration, inhibited MMP13 secretion, and showed cartilage protection in vivo. Yin et al. [89] investigated an injectable hyperbranched PEG crosslinked HA hydrogel microparticle containing miR-99a-3p-modified subcutaneous fat SC-Exos, which, with an extended exosome retention time, inhibited ADAMTS4 expression and enhanced ECM integrity and was beneficial for long-term treatment of OA. In addition, EVs derived from mouse synovial tissues have also been shown to improve OA. One study showed that BMP-7-modified SMSC-Exos promoted synovial macrophage M2 polarization and improved the pathological changes of OA [90]. Long et al. [91] isolated SMSC-Exos from mice and inhibited the release of inflammatory cytokines, ECM degradation, and autophagy defects in chondrocytes by delivering MATN3. Qiu et al. [92] showed that the delivery of miR-485-3p by exosomes derived from SMSCs promoted cartilage repair, thereby delaying the progression of OA. Yang et al. [93] reported that vascular endothelial cell-derived exosomes in mice reduced the antioxidant capacity of chondrocytes, inhibited autophagy, and exacerbated knee degeneration in mice with DMM-induced OA, which was contrary to the effect of other tissue-derived EVs on OA. Qin et al. [21] isolated mouse macrophage-derived EVs, and the M2 macrophage-derived apoptotic bodies (M2-ABs, a type of EV) were rich in miR-21-5p, which negatively correlated with articular cartilage degradation. The results of in vivo studies showed that intraarticular injection of M2-ABs alleviated ACLT-induced OA and the inflammatory response caused by M1 macrophages, prevented articular cartilage injury, and improved gait abnormalities in OA mice (Fig. S3A). On the contrary, an experiment conducted by Ebata et al. [94] suggest that macrophage-derived EVs exacerbate cartilage catabolism and the progression of OA by initiating the noncanonical pyroptosis of chondrocytes (Fig. S3B).

#### EVs derived from rats

**Fig. 3.**
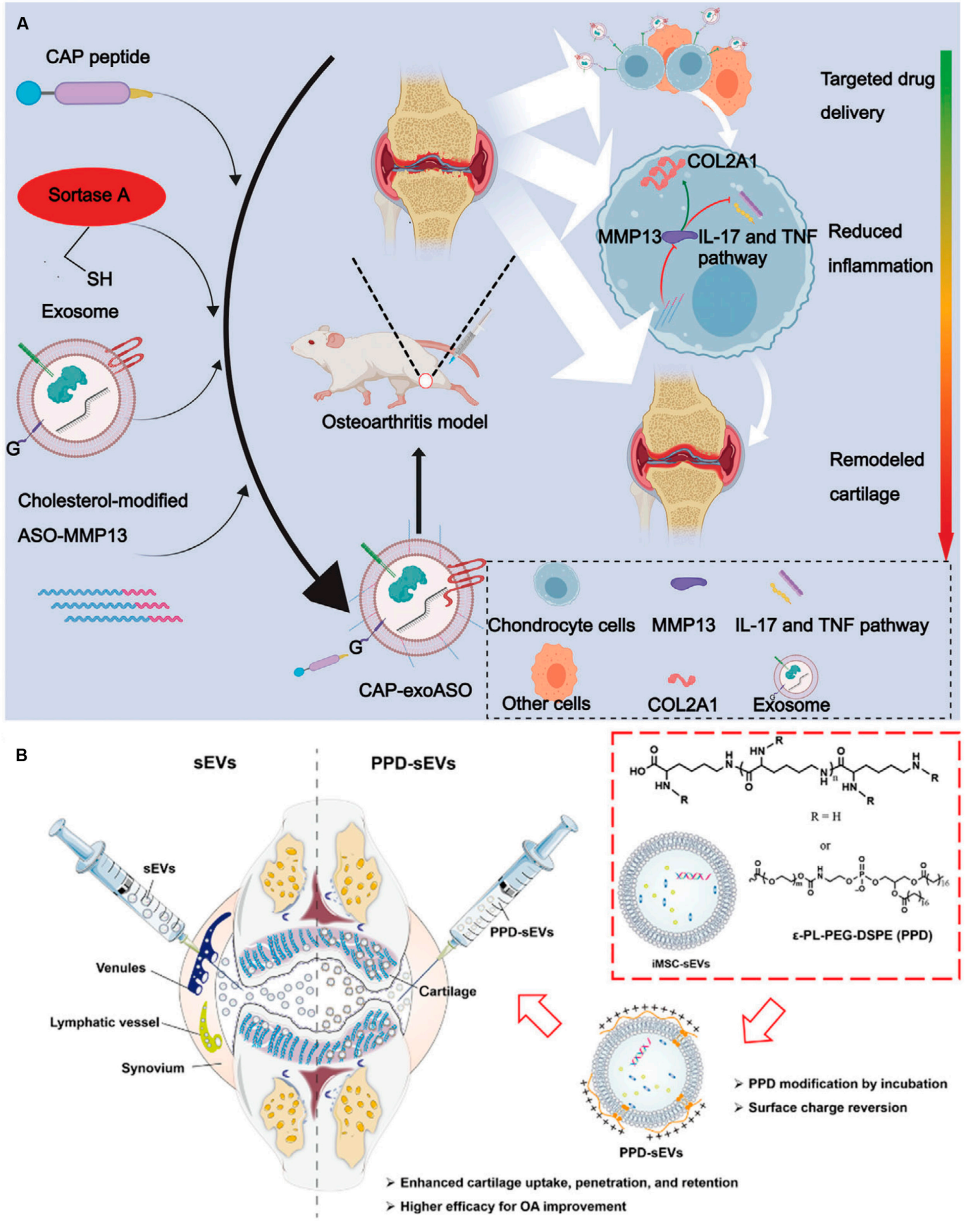
Usages of engineering-modified EVs in OA. (A) CAP-exoASOs restored joint homeostasis by silencing MMP13, reducing inflammation, and remodeling cartilage. Reproduced from [109] with permission. Copyright 2024 Wiley-VCH GmbH. (B) PPD-sEVs exhibited better therapeutic efficacy than sEVs in OA treatment. Reproduced from [110] with permission. Copyright 2021 The Authors. *Journal of Extracellular Vesicles* published by Wiley Periodicals, LLC on behalf of the International Society for Extracellular Vesicles.

Wang et al. [95,96] conducted a study wherein exosomes extracted from rat MSCs were utilized. Following treatment with transforming growth factor-β1 (TGF-β1), an overexpression of miR-135b was observed, which in turn negatively regulated Sp1. This process facilitated the polarization of M2 macrophages, thereby enhancing the repair of cartilage and promoting the proliferation of chondrocytes. Dong et al. [97] found that exosomal miR-127-3p derived from rat BMMSCs inhibited CDH11 in chondrocytes and further blocked the Wnt/β-catenin pathway, thereby protecting chondrocytes from OA injury. In another study, experimental OA in rats was induced by injection of sodium iodoacetate, and endoarticular injection of BMMSC-derived exosomes caused decreased expression of MMP13 protein and up-regulation of COL2A1 protein. In addition, exosome treatment also reduced Osteoarthritis Research Society International (OARSI) scores and joint pain in OA rats [98]. Zhang et al. [99] induced a rat model of OA by using improved Hulth technology, in which exosomes derived from rat BMMSCs were injected into the joint, which promoted the transformation of synovial macrophages from the M1 type to the M2 type, reduced the production of inflammatory cytokines, and promoted the release of anti-inflammatory factors. This reduced the infiltration of synovial inflammatory cells and damage to the articular cartilage, thereby delaying the progression of OA. Jin et al. [100] also revealed that rat BMMSC-derived exosomes have anti-inflammatory and cartilage protective effects on OA. Wan et al. [101] developed a novel photo-crosslinked spherical GelMA coated with engineered exosomes modified with a cartilage-friendly WYRGRL (W) peptide and loaded with the small inhibitor LRRK2-IN-1 (W-Exo-L@GelMA). W-Exo-L@GelMA demonstrated a strong chondrocyte targeting effect and was shown to reduce chondropathy and subchondral bone loss in mouse models of OA. Guan et al. [102] incorporated aldehyde-functionalized chondroitin sulfate (OCS) into methylacrylyl gelatin (GM) to generate GMOCS hydrogels as exosome carriers. It was found that the GMOCS-Exo hydrogel alleviated damage to chondrocytes caused by IL-1, enhanced synthesis of ECM in chondrocytes, promoted chondrogenesis, and released exosomes continuously for 14 days. Multifunctional hydrogels inspired by mussels are heat-sensitive, self-healing, and adhesive. Zhang et al. [103] studied an injectable mussel-stimulated high-adhesion hydrogel that encapsulated exosomes in an alginate-dopamine, chondroitin sulfate, and regenerated fibroin (AD/CS/RSF) hydrogel. The AD/CS/RSF/Exo hydrogel accelerated the regeneration and ECM remodeling of cartilage defects of the patellar groove in rats. In addition, Zhou et al. [104] isolated exosomes from rat synovial fibroblasts and injected CFC-miR-126-3P-Exos into the joint cavity of OA rats, which promoted chondrocyte proliferation, inhibited apoptosis and inflammation, and salvaged cartilage degeneration. Sang et al. [105] loaded primary rat chondrocyte-derived exosomes (CC-Exos) into a heat-sensitive hydrogel and found that the exosome-loaded hydrogel continued to release exosomes for 14 days and that it positively regulated chondrocyte proliferation, migration, and differentiation. Moreover, by regulating the polarization of macrophages, excessive inflammatory responses can be reduced, effectively preventing the destruction of articular cartilage. With the development of engineering-modified technology, modifying EVs and the use of engineered EVs is a feasible method in cartilage therapy [106,107]. Thomas et al. [108] isolated exosomes from L-cells stably transfected with WNT3a and detected its effect with human articular chondrocytes and cartilage defects. In vitro study showed that WNT3a was successfully loaded on exosomes and resulted in activation of WNT signaling, and in vivo experiments demonstrated that exosomes can penetrate cartilage tissue and exosomes loaded with WNT3a improved osteochondral repair. To achieve targeted delivery, exosomes were extracted from primary chondrocytes of Sprague–Dawley (SD) rats and ligated with CAP using Sortase A and subsequently incubated with cholesterol-modified antisense oligonucleotide (ASO) targeting matrix metalloproteinase-13 (ASO-MMP13) to construct a chondrocyte-targeted drug delivery exosomes (CAP-exoASO). Compared with exosomes without CAP (ExoASO), CAP-exoASOs attenuate IL-1β-induced chondrocyte damage and prolong the retention time of ASO-MMP13 in the joint without distribution in major organs following intraarticular injection. These results showed that CAP-exoASOs attenuated inflammation, promoted chondrocyte proliferation and collagen synthesis, and reduced apoptosis, thereby restoring joint homeostasis (Fig. 3A) [109]. Considering the negative charge against chondrocyte uptake, Feng et al. [110] modified MSC-sEVs with a novel amphiphilic cationic polymer named ε-polylysine-polyethylene-distearyl phosphatidylethanolamine (PPD) to reverse the surface charge from negative to positive. In vitro and in vivo experiments all demonstrated that PPD-sEVs exhibited superior therapeutic efficacy than MSC-sEVs in OA treatment (Fig. 3B).

#### EVs derived from rabbits

Rabbit CC-Exos have been reported to promote the migration, proliferation, and synthesis of cartilage matrix in cartilage progenitor cells, leading to cartilage regeneration via the TGF-β/SMAD signaling pathway. Compared with the BMSC-Exo-treated control group in one study, CC-Exo treatment maintained the stability of the constructed cartilage, reduced the inward growth of blood vessels, and prevented cartilage hypertrophy. In addition, CC-Exos also exhibited anti-angiogenic properties, which helped promote cartilage repair [111]. Qi et al. [112] isolated exosomes from rabbit BMMSCs and demonstrated that these exosomes effectively inhibited changes in the IL-1β-induced mitochondrial membrane potential, cell survival rate, apoptosis, and mitochondria-mediated apoptosis by regulating p38, ERK, and AKT signaling pathways. Chen et al. [113] used 3D printing to manufacture a cartilage ECM/GelMA/exosome scaffold with radially oriented channels using desktop-stereolithography technology. In vitro experiments showed that the scaffold effectively retained exosomes for 14 days. In a rabbit osteochondral defect model, the 3D-printed scaffolds were better at retaining exosomes for at least 7 days than controls (exosomes in PBS). The scaffolds greatly promoted cartilage and subchondral bone regeneration, reversed mitochondrial dysfunction, and polarized synovial macrophages to the M2 phenotype. In addition, researchers isolated exosomes from rabbit plasma and loaded them with miRNA-140 and showed through in vitro experiments that the exosomes promoted differentiation of BMMSCs into chondrocytes [114]. What is more, some scientists have generated an innovative approach to enhance the therapeutic efficacy of EVs from rabbit MSCs by leveraging cell co-aggregation and dynamic 3D cultures, potentially paving the way for advanced OA treatments [115].

#### EVs derived from deer

As a special organ of mammals, antlers can be completely regenerated every year. Their regeneration mainly relies on antler stem cells, which can continuously self-renew and differentiate into multi-lineage cells, thus forming organs. Compared with other types of stem cells, antler stem cells are easy to isolate and exhibit a higher ability to proliferate and differentiate [116,117]. Lei et al. [118] found that exosomes secreted by antler stem cells slowed the aging of MSCs and promoted cartilage repair and remodeling. After exosomal treatment, the aging phenotype of human MSCs improved, and the expression of related genes also changed. In a mouse model of OA induced by ACLT injury, antler stem cell-derived exosome treatment partially restored grip strength levels, alleviated bone erosion and cartilage degradation, promoted cell proliferation, and reduced cell senescence. In addition, RNA sequencing analysis also found that exosome treatment reversed gene expression patterns associated with OA [118]. After full-thickness cylindrical articular cartilage defects (2 mm in diameter, 1 mm in depth) in rat model were established through drilling surgery, hydrogels loaded with exosomes derived from deer antler stem cells (ASC-Exos) were implanted into the defects. Histologic evaluation of cartilage repair after defects indicated that the level of cartilage repair in the experimental group (ASC-Exos) was higher than that in the positive control (hADSC-Exos) and negative control (Dulbecco’s modified Eagle’s medium) groups (*P* < 0.05). In vitro experiments showed that ASC-Exos significantly enhanced the proliferation abilities of chondrocytes and the proliferation abilities and the migration abilities of BMSCs (*P* < 0.05). ASC-Exos up-regulated the expression levels of Aggrecan, COL II, and Sox9 mRNA and proteins in chondrocytes. Analysis of ASC-Exos protein components revealed the presence of active components such as transferrin (TF), S100A4, and insulin-like growth factor-binding protein 1 (IGF1). All results demonstrated that ASC-Exos have a significant effect on cartilage damage repair, which may be attributed to their promotion of chondrocyte and BMSC proliferation and migration, as well as the maintenance of chondrocyte phenotype. This effect may be mediated by the presence of TF, S100A4, and IGF1 [119].

#### EVs derived from pigs

As a clinically relevant large animal model, pigs are frequently used to detect the therapeutic efficacy in experiments of cartilage injuries and OA. In vitro study of Guo and Fan [120] demonstrated that EVs isolated from the external ears of swine promoted the proliferation and migration of ADSC, facilitated the differentiation of ADSCs into elastic chondrocytes, and maintained a characteristic phenotype with a higher expression of chondrocyte-specific markers COL2A1, ACAN, and SOX9 expression and lower expression of the fibrosis cartilage marker COL1A1 in the chondrogenic differentiation process. The conflicting results suggest that the role of EVs in cartilage repair is still unclear. More preclinical large animal experiments are needed to clarify the role of EVs in cartilage repair and the exact mechanisms involved before translational and clinical research can be carried out.

### Plants

EVs derived from various mammalian cells are widely used in various tissue regeneration fields as communication tools between cells. However, EVs of mammalian origin are isolated from cell culture supernatants, which involve time-consuming and labor-intensive processes, and therefore, these EVs are not cost-effective [121]. The EVs of plant origin are widely derived from nature, are easier to obtain, and have a higher economic benefit compared to human and animal origins [122]. In the 1960s, Jensen [123] discovered multivesicle bodies in cotton. Then, Halperin and Jensen [124] made a breakthrough: They observed polyvesicles in carrot cell cultures for the first time. EVs of plant origin have been isolated from many plants, such as ginger, lemon, tomato, grape, bitter melon, blueberry, apple, aloe vera, *Arabidopsis thaliana*, ginseng, sunflower, cabbage, yams, and others [125–128]. There is increasingly more evidence that plant EVs have anti-inflammatory, antioxidant, anti-tumor, and other biological activities in vivo and in vitro [129]. Due to the advantageous extraction of plant exosome-like nanovesicles (PELNVs) (Fig. S4A) [130], they are appropriate modalities for drug delivery and tissue regeneration (Fig. S4B) [122]. Studies have also suggested the potential role of plant-derived EVs in promoting cartilage tissue formation. Yıldırım et al. [131] isolated EVs from lemons and tomatoes. In experiments, they treated cells with different concentrations of lemon- and tomato-derived exosomes and found that 100 μg/ml was the best dose for treating cells, and both exosomes promoted cell proliferation. In addition, tomato-derived exosome-like vesicles (TELVs) promoted the differentiation of adipose stem cells into chondrocytes and up-regulated the expression of genes associated with chondrogenesis, such as COL2, ACAN, and SOX9. In contrast, lemon-derived exosome-like vesicles (LELVs) down-regulated the expression of genes involved in chondrogenesis. This suggests that plant-derived exosome-like vesicles play an important role in chondrogenesis of adipose stem cells.

### Bacteria

**Fig. 4.**
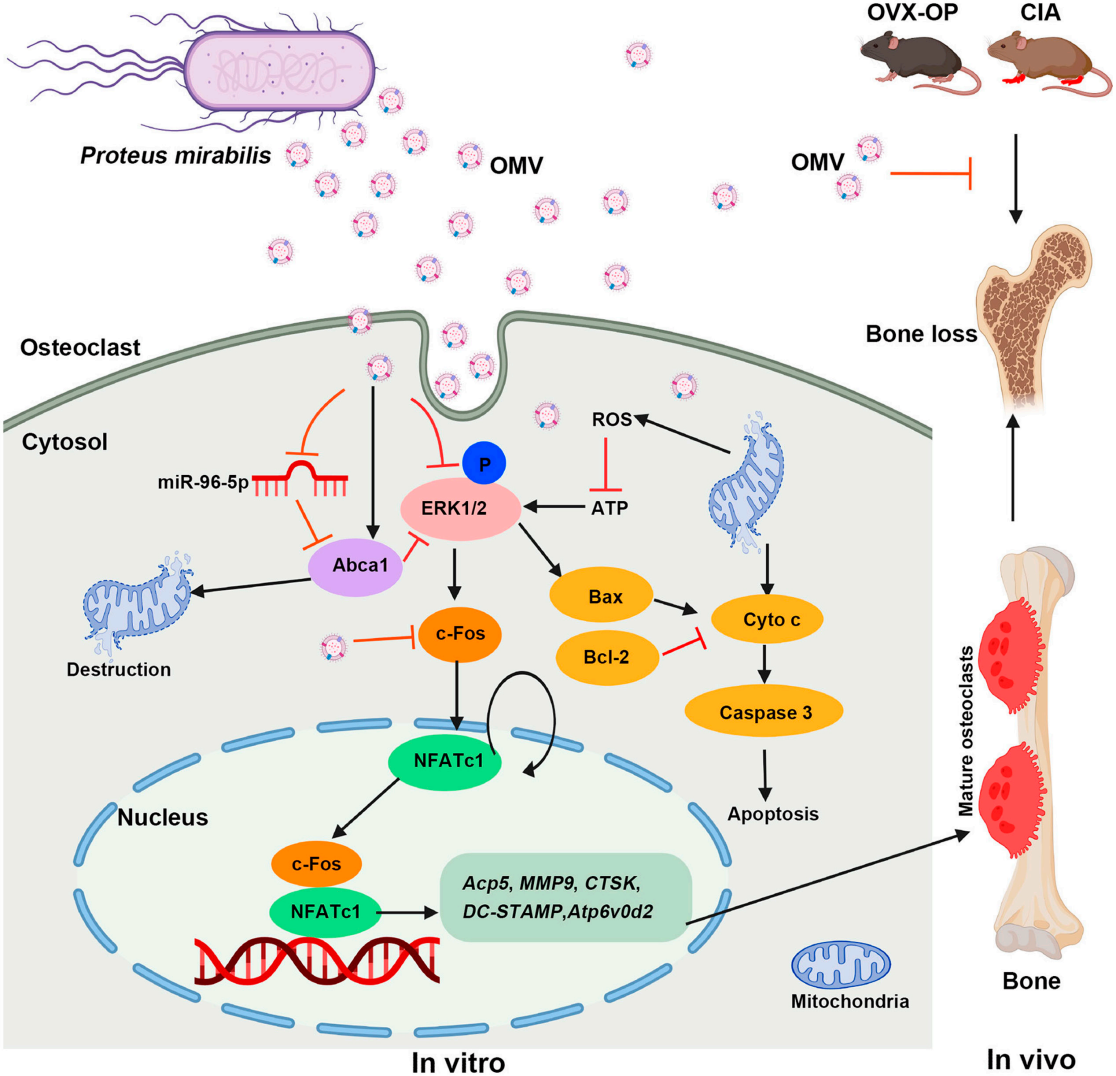
*P. mirabilis* vesicles inhibit bone loss not only in the ovariectomy (OVX) model but also in the subchondral bone of mice with collagen-induced arthritis (CIA). Reproduced from [136] with permission. Copyright 2022 Wang et al. Distributed under CC BY. Published by Frontiers Media S.A.

Bacteria are a wide range of microorganisms. Compared with mammals and plants, bacteria can reproduce on a large scale, and their extraction rate of EVs is much higher. The outer membranes of gram-negative bacteria and the plasma membranes of gram-positive bacteria produce bacterial EVs through bubble and lysate biogenetic pathways [132]. Gut microbiota plays a crucial role in OA, and changes in the gut microbiota may alter or even reverse the progression of OA [133]. Through in vivo animal experiments, Liu et al. [134] found that milk-derived EVs (mEVs) alleviate the progression of OA by increasing potential beneficial bacteria (*Firmicutes*, *Ruminococcaceae*, *Akkermansiaceae*) and reducing pro-inflammatory bacteria (*Proteobacteria*). EVs generated by gut bacteria have been identified as important mediators in microbiota–host interaction [135]. Bacterial EVs from *Proteus mirabilis* are revealed to exert an inhibitory effect on osteoclastogenesis and bone resorption not only in the ovariectomy model but also in subchondral bone of mice with CIA (Fig. 4) [136]. Research of Liu et al. [137] suggested that exosomes (one of EVs) produced by osteoclast up-regulate the expression of chondrodegradation-related proteins including ADAMTS5 and MMP13. That is to say, bacterial EVs from *P. mirabilis* may restrain cartilage degeneration by suppressing osteoclast activity and down-regulating cartilage degradation-related genes. Although there are currently no direct experiments of bacterial EVs on cartilage or OA, it is considered a viable option to delay the progression of OA [138]. Table S1 shows the typical sources, characterization, different delivery methods, and potential mechanisms of EVs in cartilage repair.

## Mechanisms of EVs Captured by Injured Chondrocytes

**Fig. 5.**
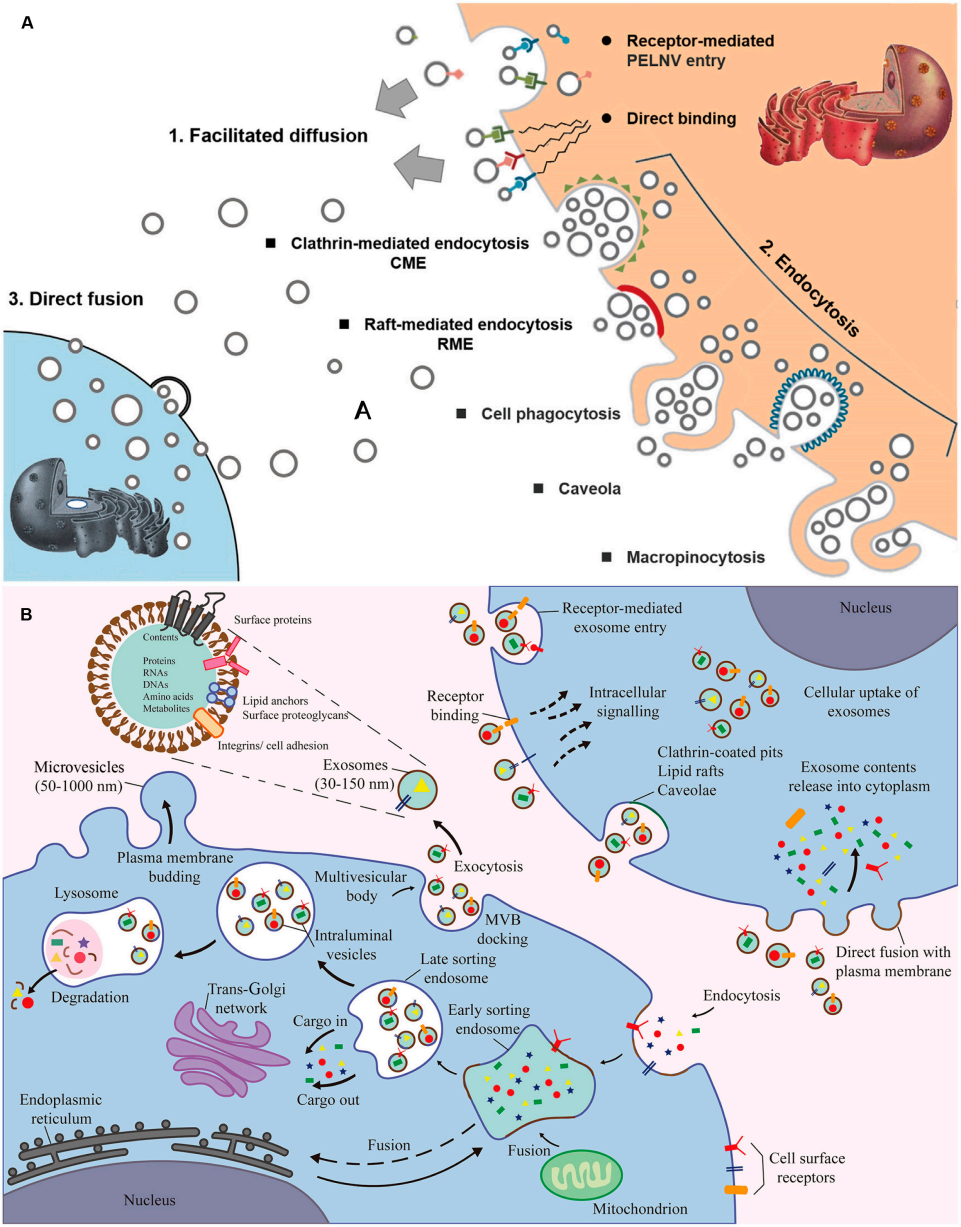
Mechanisms of EVs captured by injured chondrocytes. (A) Communication between EVs and surrounding cells. Reproduced from [130] with permission. Copyright 2020 The American Society of Gene and Cell Therapy. (B) Multiple pathways of cellular uptake of exosomes. Reproduced from [139] with permission. Copyright 2021 Li et al. Distributed under CC BY. Published by Frontiers Media S.A.

After being released from donor cells, EVs transfer multiple biomolecules to the recipient cells, such as small RNAs, mRNAs, microRNAs (miRNAs), other noncoding RNAs along with lipids and DNA, proteins, lipids, and other pharmacologically active metabolites. EVs communicate with surrounding cells mainly through 3 ways: facilitated diffusion (direct binding, receptor-mediated entry, etc.), endocytosis (clathrin-mediated endocytosis, raft-mediated endocytosis, cell phagocytosis, caveola, macropinocytosis, etc.), and direct fusion (Fig. 5A) [130]. Uptake pathways of EV subpopulations are different. According to the current research, the pathways of cellular uptake of exosomes comprise receptor-mediated exosome entry, receptor binding, clathrin-coated pits, lipid rafts, caveolae, and direct fusion (Fig. 5B) [139]. With a diameter of approximately 30 to 150 nm, exosomes are the most studied EV. In an investigation of exploring the mechanisms of exosomes derived from miRNA-210-overexpressing BMSCs (BMSCs-210-Exos) protecting injured chondrocytes from LPS-induced injury, He et al. [140] found that exosomes endocytose into chondrocytes to inhibit tumor necrosis factor receptor superfamily member 21 (Tnfrsf21) and weaken the NF-κB pathway. Using a new real-time method [fluorescence lifetime imaging microscopy (FLIM)] to investigate EV kinetics with living cells, Saari et al. [141] found that exosomes deliver the drug mainly by endocytosis. Based on the 2 studies, we conclude that exosomes captured by injured chondrocytes through endocytosis, but the specific mechanism needs to be further studied. Meanwhile, on detecting the cellular uptake mechanism of different EV subtypes, loaded with paclitaxel, they revealed that microvesicles deliver the drug by both endocytosis and cell membrane fusion. As to the mechanisms by which apoptotic bodies are taken up by cells, no authoritative studies have been found. Overall, the mechanism by which EVs are captured by injured chondrocytes is still unclear, and more research in this area is urgently needed.

## Quantification and Storage of EVs

With the development of EV-related research, the qualitative, quantitative, and storage of EVs have become an urgent problem before clinical research. First, we must mention the definition of EVs. There are a large number of communication particles between cells, but not all particles are the EVs mentioned in this article. The EVs mentioned in this article refer to the spontaneous formation of cells, rather than extrusion or other causes. As shown in Table S2, MISEV2023 provides a clear nomenclature proposals among EVs, extracellular particles, and other vesicles [22]. For practical purposes, developing metrics to map aspects of EV biology and therapeutic potency onto quantifiable features is undoubtedly the ultimate goal [142]. The identification of EVs includes evaluating the presence of EVs, the number of EVs, and the ratio of EVs to non-EVs. Due to the lack of specific markers and overlapping size range, the current EV preparations including “exosome” preparations are heterogeneous with undefined biogenesis origin and undetermined purity. As a result, no single method could identify all EVs, and the identification must be evaluated by multiple methods. Exosomes from different sources have different protein, lipid, nucleic acid, and other biomolecule content, and their surface antigen markers are also different. Although these 3 proteins CD9, CD63, and CD81 have been reported to be present on the surface of most exosomes, the absence of these 3 proteins does not necessarily mean that they are not exosomes. In other words, even the most studied exosomes currently lack specific protein markers, let alone microvesicles, and apoptotic bodies. According to MISEV2023, EV preparation should be defined by quantitative measures of the source of EVs (e.g., number of secreting cells, volume of biofluid, and mass of tissue), tested for the presence of components associated with EV subtypes or EVs generically, provided an indication of the instrument/method limit of detection (LOD) when EVs are characterized with quantitative metrics, approximations of the abundance of EVs (particle number, protein, and/or lipid content), and the ratio of EVs to non-EVs should be detected. A potency test is an ideal tool to define the identity and predict the therapeutic effectiveness of EVs, but the diverse underlying pathologies of EVs against multiple diseases are not easy to fully elucidate [143]. Although lacking specificity and sensitivity, quantification of particle number concentration, particle size, total protein, total lipids, total RNA, characterization of EVs by morphology and protein composition, non-protein markers of EVs, and localization of EV-associated components can be used to evaluate the quality of EVs comprehensively. In order to ensure the reliability and repeatability of the experiment, the quantification detection cannot be avoided. Drawing on methods from published literature, flow cytometry-based methods (bead-based flow cytometry, single-EV flow cytometry), genetic protein tagging, mass spectrometry proteomics, microscopy-based methods (atomic force microscopy, diffraction-limited fluorescence microscopy, dynamic light scattering, electron microscopy, NTA, single-particle interferometric reflectance imaging sensing, super-resolution microscopy), nucleic acid characterization, protein and non-protein labeling of EVs, Raman spectroscopy, resistive pulse sensing, and WB all can be used to quantify EVs [22].

EVs are derived from living cells or tissues, and living cells and tissues are sensitive to changes in the surrounding environment. However, unlike living cells, EVs are nonactive and nonrenewable and can only maintain vitality for a short time. Therefore, how to store and maintain its efficient vitality is particularly important. The influence of cell and tissue preservation before and after separation on the quality of EV extraction was also significant. Due to the diversity of EV tissue sources, it remains to be further studied whether preserving the prerequisite tissues or cells of EV extraction is more conducive to preserving the activity of EVs or directly preserving EVs is more conducive to preserving the activity of EVs. Both pre-separation and post-separation storage techniques are under development. Comparative research should be actively researched in a long-term process. Considering that pre-separation storage influences the characterization of EVs, care should be taken to minimize environmental impacts and possible bacterial or other microbial infections prior to separation. Strictly recording all storage parameters before and after EV separation (including use of preservatives or cryoprotectants, temperature, time, freezing procedure, storage vessel, number of freeze–thaw cycles, and thawing method) is equally important for other research groups to replicate experimental results and improve experimental conditions. The isolated EVs can be preserved for a period of time under freezing conditions, but it is recommended to avoid repeated freezing and thawing. Although other temperatures have been studied, the current recommended temperature for long-term preservation is −80 °C. Much remains unknown in this region, but a preliminary expert consensus has begun to emerge, as described in more detail in MISEV2023 [22].

## Conclusions and Perspectives

EVs are nanoscale vesicles produced by paracrine secretion, which have made important progress in the field of cartilage repair and regenerative medicine in recent years. In experimental research, EVs have been shown to be effective in promoting chondrocyte proliferation and matrix synthesis, especially EVs derived from stem cells, such as MSCs, which are rich in growth factors and signaling molecules associated with promoting chondrogenesis. In addition, by changing the source cells of EVs or pretreating and modifying EVs, their ability to promote cartilage repair can be further enhanced. Different tissue-derived EVs have different effects on cartilage regeneration. Li et al. [144] isolated and identified EVs derived from adipose, bone marrow, and SMSCs. All 3 EVs promoted MSC viability and migration, and all showed good chondrogenic and osteogenic abilities, but ADSC-EVs were the most effective. Another study showed that exosomes derived from ADSCs were capable of stimulating cartilage formation [145]. Although some studies have revealed the positive role of EVs in cartilage repair, the specific mechanism of action still needs to be further explored. At the clinical application level, EVs face challenges such as standardization of production, mass production, storage stability, and precise targeted delivery. One clinical study showed that a single injection of BMMSC-derived EVs in the joint cavity showed pain reduction and joint function relief after 6 months of follow-up. BMMSC-derived EVs have been shown to be safe and effective in the treatment of OA [146]. EVs derived from human tissues do not present immunogenic problems, but with the aging of the body, age-related EVs are also produced, which may have adverse effects on treatment. Other mammals are relatively close to humans, but extracting EVs from these origins is time-consuming and laborious. Plant origin is more extensive, and the cost of plant-derived EVs is lower, but plant EVs lack clear identification markers. The source of bacteria is more abundant and its amplified yield is higher, but it is also easy to receive the contamination of miscellaneous bacteria. The advantages and disadvantages of mammalian, plant, and bacterial EVs are listed in Table. There are still relatively few clinical trials on EVs, however, and clinical studies on EVs in the field of cartilage repair need to be strengthened to evaluate their safety and efficacy. Moreover, a long-term tracking mechanism should be established to monitor the therapeutic effect and possible long-term side effects, providing solid data support for the clinical application of EVs.

**Table.** Advantages and disadvantages of EVs derived from mammals, plants, and bacteria

Origins of EVs	Advantages	Disadvantages
Mammals	Homology	Immunogenicity, lack of clinical studies
Human	Autogenous tissue or cell source	Low cost-effectiveness, time-consuming, and laborious
Human umbilical cord mesenchymal stem cells (hUCMSCs)	Strong differentiation potential and multi-lineage differentiation ability	The activity of autologous tissue decreased with the prolongation of preservation time, and the allogeneic tissue may have immunogenicity
Human embryonic mesenchymal stem cells (hEMSCs)	The immunogenicity of stem cells was lower than that of umbilical cord stem cells	The activity of autologous tissue decreased with the prolongation of preservation time, and the allogeneic tissue may have immunogenicity
Human synovial mesenchymal stem cells (hSMSCs)	Maintaining cartilage homeostasis, meditate the crosstalk between the synovium and cartilage	Synovial tissue is not easy to classify and purify, and may include other tissues.
Human bone marrow mesenchymal stem cells (hBMMSCs or hBMSCs)	Stem cells persistently exist in the human body and have a strong ability to expand and differentiate	Reduced capacity of these cells for self-renewal, proliferation, and differentiation with increasing donor age
Human adipose-derived mesenchymal stem cells (hADSCs)	A lower risk of donor site morbidity	May contain EVs associated with fat promotion
Human chondrocytes	Exhibiting homologous functions	Chondrocyte phenotype is easily lost during amplification, resulting in impure exosomes extracted
Mice/rats	Individual sources are abundant, and fecundity is strong	Research results are inconsistent
Rabbits	Routine laboratory animals prior to clinically relevant large animal experiments	The reliability of the experimental results is more reliable than that of mice/rats experiments, but worse than that of clinically relevant large animal experiments
Deer	Stem cells proliferate more vigorously than other mammals	Immunogenicity and biocompatibility should be examined
Pigs	High homology with human gene	Possible transmission of zoonotic diseases
Plants	Natural provenance, no zoonotic or human pathogens, nonimmunogenic and innocuous traits, readily accessible, cost-effective, and low-risk solutions, high yield, and reproducibility.	Variations in plant sources, growth conditions, and extraction protocols can impact the composition and functionality. Markers of EVs have not yet been clearly reported and demand further investigation. Lack of clinical studies
Bacteria	Harvested from mono- or polymicrobial culture in vitro, in vivo/ex vivo sources such as body fluids or feces, and environmental samples ranging from soil to seawater	EVs from nontarget species are likely present. Lack of clinical studies

In recent years, researchers have developed a variety of tissue engineering strategies for improving the therapeutic effect of EVs, providing more possibilities for the targeted delivery and controlled release of EVs, but there are still many challenges. Further optimization of the material properties of hydrogels and scaffolds, improvement of their stability and biocompatibility, and exploration of more carrier design strategies are expected to make major breakthroughs in the field of drug delivery and tissue repair. In conclusion, EVs, as a type of cell-free therapy, have broad application prospects and potential in cartilage repair. With continued progress in technology and in-depth research, it is believed that EVs will provide more effective and reliable means and methods for the treatment of cartilage injury and other diseases.

## Data Availability

The data are freely available upon request.

## References

[1] Roelofs AJ, De Bari C. Osteoarthritis year in review 2023: Biology. Osteoarthr Cartil. 2024;32(2):148–158. 10.1016/j.joca.2023.11.002 37944663

[2] Hunter DJ, Bierma-Zeinstra S. Osteoarthritis. Lancet. 2019;393(10182):1745–1759. 31034380 10.1016/S0140-6736(19)30417-9

[3] Long H, Liu Q, Yin H, Wang K, Diao N, Zhang Y, Lin J, Guo A. Prevalence trends of site-specific osteoarthritis from 1990 to 2019: Findings from the global burden of disease study 2019. Arthritis Rheumatol. 2022;74(7):1172–1183. 35233975 10.1002/art.42089 PMC9543105

[4] Wong AY, Samartzis D, Maher C. The global burden of osteoarthritis: Past and future perspectives. Lancet Rheumatol. 2023;5(9):e496–e497. 38251491 10.1016/S2665-9913(23)00207-2

[5] Richard MJ, Driban JB, McAlindon TE. Pharmaceutical treatment of osteoarthritis. Osteoarthr Cartil. 2023;31(4):458–466. 10.1016/j.joca.2022.11.005 36414224

[6] Li S, Cao P, Chen T, Ding C. Latest insights in disease-modifying osteoarthritis drugs development. Ther Adv Musculoskelet Dis. 2023;15:1759720X231169839. 10.1177/1759720X231169839 PMC10184265 37197024

[7] Wilken F, Buschner P, Benignus C, Behr AM, Rieger J, Beckmann J. Pharmatherapeutic treatment of osteoarthrosis—Does the pill against already exist? A narrative review. J Pers Med. 2023;13(7):1087. 37511701 10.3390/jpm13071087 PMC10381646

[8] Pandey P, Singh R, Srivastava S, Mishra MK. A review on osteoarthritis and its eradication approaches: An update. Curr Drug Res Rev. 2024. 10.2174/0125899775267865231115051056 38284719

[9] Klemt C, Padmanabha A, Esposito JG, Laurencin S, Smith EJ, Kwon YM. Elevated ESR and CRP prior to second-stage reimplantation knee revision surgery for periprosthetic joint infection are associated with increased reinfection rates. J Knee Surg. 2023;36(4):354–361. 34375998 10.1055/s-0041-1733902

[10] Martinez R, Chen AF. Outcomes in revision knee arthroplasty: Preventing reoperation for infection Keynote lecture—BASK annual congress 2023. Knee. 2023;43:A5–A10. 37524637 10.1016/j.knee.2023.07.010

[11] Wu M, Zheng K, Li W, He W, Qian C, Lin Z, Xiao H, Yang H, Xu Y, Wei M, et al. Nature-inspired strategies for the treatment of osteoarthritis. Adv Funct Mater. 2023;34(4):2305603.

[12] Li M, Yin H, Yan Z, Li H, Wu J, Wang Y, Wei F, Tian G, Ning C, Li H, et al. The immune microenvironment in cartilage injury and repair. Acta Biomater. 2022;140:23–42. 34896634 10.1016/j.actbio.2021.12.006

[13] Rim YA, Nam Y, Ju JH. The role of chondrocyte hypertrophy and senescence in osteoarthritis initiation and progression. Int J Mol Sci. 2020;21(7):2358. 32235300 10.3390/ijms21072358 PMC7177949

[14] Rapp AE, Zaucke F. Cartilage extracellular matrix-derived matrikines in osteoarthritis. Am J Physiol Cell Physiol. 2023;324(2):C377–C394. 36571440 10.1152/ajpcell.00464.2022

[15] Boulestreau J, Maumus M, Jorgensen C, Noel D. Extracellular vesicles from mesenchymal stromal cells: Therapeutic perspectives for targeting senescence in osteoarthritis. Adv Drug Deliv Rev. 2021;175: Article 113836. 34166759 10.1016/j.addr.2021.113836

[16] Li M, Fang F, Sun M, Zhang Y, Hu M, Zhang J. Extracellular vesicles as bioactive nanotherapeutics: An emerging paradigm for regenerative medicine. Theranostics. 2022;12(11):4879–4903. 35836815 10.7150/thno.72812 PMC9274746

[17] Wan R, Liu S, Feng X, Luo W, Zhang H, Wu Y, Chen S, Shang X. The revolution of exosomes: From biological functions to therapeutic applications in skeletal muscle diseases. J Orthop Translat. 2024;45:132–139. 38544740 10.1016/j.jot.2024.01.001 PMC10966453

[18] Zeng ZL, Xie H. Mesenchymal stem cell-derived extracellular vesicles: A possible therapeutic strategy for orthopaedic diseases: A narrative review. Biomater Transl. 2022;3(3):175–187. 36654775 10.12336/biomatertransl.2022.03.002 PMC9840092

[19] Dixson AC, Dawson TR, Di Vizio D, Weaver AM. Context-specific regulation of extracellular vesicle biogenesis and cargo selection. Nat Rev Mol Cell Biol. 2023;24(7):454–476. 36765164 10.1038/s41580-023-00576-0 PMC10330318

[20] Radler J, Gupta D, Zickler A, Andaloussi SE. Exploiting the biogenesis of extracellular vesicles for bioengineering and therapeutic cargo loading. Mol Ther. 2023;31(5):1231–1250. 36805147 10.1016/j.ymthe.2023.02.013 PMC10188647

[21] Qin L, Yang J, Su X, Xilan L, Lei Y, Dong L, et al. The miR-21-5p enriched in the apoptotic bodies of M2 macrophage-derived extracellular vesicles alleviates osteoarthritis by changing macrophage phenotype. Genes Dis. 2023;10(3):1114–1129. 37396516 10.1016/j.gendis.2022.09.010 PMC10308169

[22] Welsh JA, Goberdhan DCI, O’Driscoll L, Buzas EI, Blenkiron C, Bussolati B, Cai H, di Vizio D, Driedonks TAP, Erdbrügger U, et al. Minimal information for studies of extracellular vesicles (MISEV2023): From basic to advanced approaches. J Extracell Vesicles. 2024;13(2): Article e12404. 38326288 10.1002/jev2.12404 PMC10850029

[23] Thery C, Witwer KW, Aikawa E, Alcaraz MJ, Anderson JD, Andriantsitohaina R, Antoniou A, Arab T, Archer F, Atkin-Smith GK, et al. Minimal information for studies of extracellular vesicles 2018 (MISEV2018): A position statement of the International Society for Extracellular Vesicles and update of the MISEV2014 guidelines. J Extracell Vesicles. 2018;7(1):1535750. 30637094 10.1080/20013078.2018.1535750 PMC6322352

[24] He Y, Xing Y, Jiang T, Wang J, Sang S, Rong H, Yu F. Fluorescence labeling of extracellular vesicles for diverse bio-applications in vitro and in vivo. Chem Commun (Camb). 2023;59(44):6609–6626. 37161668 10.1039/d3cc00998j

[25] Chen X, Tang J, Zhao Y, Wang R, Sang S, Yu F, Xing Y. Sensitive phenotyping of serum extracellular vesicles on a SERS-microfluidic platform for early-stage clinical diagnosis of ovarian carcinoma. Biosens Bioelectron. 2025;267:116724. 39260102 10.1016/j.bios.2024.116724

[26] Cheng L, Hill AF. Therapeutically harnessing extracellular vesicles. Nat Rev Drug Discov. 2022;21(5):379–399. 35236964 10.1038/s41573-022-00410-w

[27] Kisielewska M, Rakoczy K, Skowron I, Gorczynska J, Kacer J, Bochenska A, Choromańska A. Utilizing extracellular vesicles for eliminating ‘unwanted molecules’: Harnessing nature’s structures in modern therapeutic strategies. Molecules. 2024;29(5):948. 38474460 10.3390/molecules29050948 PMC10935043

[28] Li Y, Zhang H, Jiang Y, Yang J, Cai D, Bai X. The application of extracellular vesicles in orthopedic diseases. Interdiscip Med. 2024;2(3): Article e20230055.

[29] Hu H, Dong L, Bu Z, Shen Y, Luo J, Zhang H, Zhao S, Lv F, Liu Z. miR-23a-3p-abundant small extracellular vesicles released from Gelma/nanoclay hydrogel for cartilage regeneration. J Extracell Vesicles. 2020;9(1):1778883. 32939233 10.1080/20013078.2020.1778883 PMC7480606

[30] Sun T, Feng Z, He W, Li C, Han S, Li Z, Guo R Novel 3D-printing bilayer GelMA-based hydrogel containing BP, β -TCP and exosomes for cartilage-bone integrated repair. Biofabrication 2023;16(1):015008. 10.1088/1758-5090/ad04fe 37857284

[31] Guo J, Wang F, Hu Y, Luo Y, Wei Y, Xu K, Zhang H, Liu H, Bo L, Lv S, et al. Exosome-based bone-targeting drug delivery alleviates impaired osteoblastic bone formation and bone loss in inflammatory bowel diseases. Cell Rep Med. 2023;4(1): Article 100881. 36603578 10.1016/j.xcrm.2022.100881 PMC9873828

[32] Wang J, Li X, Wang S, Cui J, Ren X, Su J. Bone-targeted exosomes: Strategies and applications. Adv Healthc Mater. 2023;12(18): Article e2203361. 36881547 10.1002/adhm.202203361

[33] Bertolino GM, Maumus M, Jorgensen C, Noel D. Therapeutic potential in rheumatic diseases of extracellular vesicles derived from mesenchymal stromal cells. Nat Rev Rheumatol. 2023;19(11):682–694. 37666995 10.1038/s41584-023-01010-7

[34] Zhao S, Xiu G, Wang J, Wen Y, Lu J, Wu B, Wang G, Yang D, Ling B, du D, et al. Engineering exosomes derived from subcutaneous fat MSCs specially promote cartilage repair as miR-199a-3p delivery vehicles in osteoarthritis. J Nanobiotechnology. 2023;21(1):341. 37736726 10.1186/s12951-023-02086-9 PMC10515007

[35] Roszkowski S. Therapeutic potential of mesenchymal stem cell-derived exosomes for regenerative medicine applications. Clin Exp Med. 2024;24(1):46. 38427086 10.1007/s10238-023-01282-z PMC10907468

[36] Li Q, Li B, Ye T, Xu W, Yin H, Deng Z, Li H, Yan X, Hao X, Li L, et al. Requirements for human mesenchymal stem cell-derived small extracellular vesicles. Interdiscip Med. 2023;1(1): Article e20220015.

[37] Arutyunyan I, Elchaninov A, Makarov A, Fatkhudinov T. Umbilical cord as prospective source for mesenchymal stem cell-based therapy. Stem Cells Int. 2016;2016:6901286. 27651799 10.1155/2016/6901286 PMC5019943

[38] Zhou H, Shen X, Yan C, Xiong W, Ma Z, Tan Z, Wang J, Li Y, Liu J, Duan A, et al. Extracellular vesicles derived from human umbilical cord mesenchymal stem cells alleviate osteoarthritis of the knee in mice model by interacting with METTL3 to reduce m6A of NLRP3 in macrophage. Stem Cell Res Ther. 2022;13(1):322. 35842714 10.1186/s13287-022-03005-9 PMC9288728

[39] Li P, Lv S, Jiang W, Si L, Liao B, Zhao G, Xu Z, Wang L, Zhang J, Wu H, et al. Exosomes derived from umbilical cord mesenchymal stem cells protect cartilage and regulate the polarization of macrophages in osteoarthritis. Ann Transl Med. 2022;10(18):976. 36267713 10.21037/atm-22-3912 PMC9577719

[40] Li K, Yan G, Huang H, Zheng M, Ma K, Cui X, Lu D, Zheng L, Zhu B, Cheng J, et al. Anti-inflammatory and immunomodulatory effects of the extracellular vesicles derived from human umbilical cord mesenchymal stem cells on osteoarthritis via M2 macrophages. J Nanobiotechnology. 2022;20(1):38. 35057811 10.1186/s12951-021-01236-1 PMC8771624

[41] Cao H, Chen M, Cui X, Liu Y, Liu Y, Deng S, Yuan T, Fan Y, Wang Q, Zhang X. Cell-free osteoarthritis treatment with sustained-release of chondrocyte-targeting exosomes from umbilical cord-derived mesenchymal stem cells to rejuvenate aging chondrocytes. ACS Nano. 2023;17(14):13358–13376. 37439514 10.1021/acsnano.3c01612

[42] Wang S, Jiang W, Lv S, Sun Z, Si L, Hu J, Yang Y, Qiu D, Liu X, Zhu S, et al. Human umbilical cord mesenchymal stem cells-derived exosomes exert anti-inflammatory effects on osteoarthritis chondrocytes. Aging (Albany NY). 2023;15(18):9544–9560. 37724890 10.18632/aging.205034 PMC10564422

[43] Liu W, Liu A, Li X, Sun Z, Sun Z, Liu Y, Wang G, Huang D, Xiong H, Yu S, et al. Dual-engineered cartilage-targeting extracellular vesicles derived from mesenchymal stem cells enhance osteoarthritis treatment via miR-223/NLRP3/pyroptosis axis: Toward a precision therapy. Bioact Mater. 2023;30:169–183. 37593145 10.1016/j.bioactmat.2023.06.012 PMC10429745

[44] Zhang S, Chuah SJ, Lai RC, Hui JHP, Lim SK, Toh WS. MSC exosomes mediate cartilage repair by enhancing proliferation, attenuating apoptosis and modulating immune reactivity. Biomaterials. 2018;156:16–27. 29182933 10.1016/j.biomaterials.2017.11.028

[45] Zhang S, Chu WC, Lai RC, Lim SK, Hui JH, Toh WS. Exosomes derived from human embryonic mesenchymal stem cells promote osteochondral regeneration. Osteoarthr Cartil. 2016;24(12):2135–2140. 10.1016/j.joca.2016.06.022 27390028

[46] Wang Y, Yu D, Liu Z, Zhou F, Dai J, Wu B, Zhou J, Heng BC, Zou XH, Ouyang H, et al. Exosomes from embryonic mesenchymal stem cells alleviate osteoarthritis through balancing synthesis and degradation of cartilage extracellular matrix. Stem Cell Res Ther. 2017;8(1):189. 28807034 10.1186/s13287-017-0632-0 PMC5556343

[47] Zhang S, Teo KYW, Chuah SJ, Lai RC, Lim SK, Toh WS. MSC exosomes alleviate temporomandibular joint osteoarthritis by attenuating inflammation and restoring matrix homeostasis. Biomaterials. 2019;200:35–47. 30771585 10.1016/j.biomaterials.2019.02.006

[48] Huang C, Zhao Y, Lin S, Li L, Guo X, Yumiseba S, Yang JD, Hariri R, Ye Q, He S, et al. Characterization of human placenta-derived exosome (pExo) as a potential osteoarthritis disease modifying therapeutic. Arthritis Res Ther. 2023;25(1):229. 38017556 10.1186/s13075-023-03219-z PMC10683254

[49] Wong KL, Zhang S, Wang M, Ren X, Afizah H, Lai RC, Lim SK, Lee EH, Hui JHP, Toh WS. Intra-articular injections of mesenchymal stem cell exosomes and hyaluronic acid improve structural and mechanical properties of repaired cartilage in a rabbit model. Arthroscopy. 2020;36(8):2215–2228.e2. 32302651 10.1016/j.arthro.2020.03.031

[50] Zhang S, Wong KL, Ren X, Teo KYW, Afizah H, Choo ABH, Lai RC, Lim SK, Hui JHP, Toh WS. Mesenchymal stem cell exosomes promote functional osteochondral repair in a clinically relevant porcine model. Am J Sports Med. 2022;50(3):788–800. 35099327 10.1177/03635465211068129

[51] Hede KTC, Christensen BB, Olesen ML, Thomsen JS, Foldager CB, Toh WS, Lim SK, Lind MC. Mesenchymal stem cell extracellular vesicles as adjuvant to bone marrow stimulation in chondral defect repair in a minipig model. Cartilage. 2021;13(2_suppl):254S–266S. 34308681 10.1177/19476035211029707 PMC8804773

[52] Li N, Gao J, Mi L, Zhang G, Zhang L, Zhang N, Huo R, Hu J, Xu K. Synovial membrane mesenchymal stem cells: Past life, current situation, and application in bone and joint diseases. Stem Cell Res Ther. 2020;11(1):381. 32894205 10.1186/s13287-020-01885-3 PMC7487958

[53] Duan A, Shen K, Li B, Li C, Zhou H, Kong R, Shao Y, Qin J, Yuan T, Ji J, et al. Extracellular vesicles derived from LPS-preconditioned human synovial mesenchymal stem cells inhibit extracellular matrix degradation and prevent osteoarthritis of the knee in a mouse model. Stem Cell Res Ther. 2021;12(1):427. 34321073 10.1186/s13287-021-02507-2 PMC8317426

[54] Tao SC, Yuan T, Zhang YL, Yin WJ, Guo SC, Zhang CQ. Exosomes derived from miR-140-5p-overexpressing human synovial mesenchymal stem cells enhance cartilage tissue regeneration and prevent osteoarthritis of the knee in a rat model. Theranostics. 2017;7(1):180–195. 28042326 10.7150/thno.17133 PMC5196895

[55] Wang Z, Yan K, Ge G, Zhang D, Bai J, Guo X, Zhou J, Xu T, Xu M, Long X, et al. Exosomes derived from miR-155-5p-overexpressing synovial mesenchymal stem cells prevent osteoarthritis via enhancing proliferation and migration, attenuating apoptosis, and modulating extracellular matrix secretion in chondrocytes. Cell Biol Toxicol. 2021;37(1):85–96. 33099657 10.1007/s10565-020-09559-9

[56] Qiu M, Liu D, Fu Q. MiR-129-5p shuttled by human synovial mesenchymal stem cell-derived exosomes relieves IL-1β induced osteoarthritis via targeting HMGB1. Life Sci. 2021;269: Article 118987. 33417958 10.1016/j.lfs.2020.118987

[57] Zheng T, Li Y, Zhang X, Xu J, Luo M. Exosomes derived from miR-212-5p overexpressed human synovial mesenchymal stem cells suppress chondrocyte degeneration and inflammation by targeting ELF3. Front Bioeng Biotechnol. 2022;10: Article 816209. 35284413 10.3389/fbioe.2022.816209 PMC8908902

[58] Kong R, Zhang J, Ji L, Yu Y, Gao J, Zhao D. Synovial mesenchymal stem cell-derived exosomal microRNA-320c facilitates cartilage damage repair by targeting ADAM19-dependent Wnt signalling in osteoarthritis rats. Inflammopharmacology. 2023;31(2):915–926. 36862227 10.1007/s10787-023-01142-y

[59] Han B, Fang W, Yang Z, Wang Y, Zhao S, Hoang BX, Vangsness CT Jr. Enhancement of chondrogenic markers by exosomes derived from cultured human synovial fluid-derived cells: A comparative analysis of 2D and 3D conditions. Biomedicines. 2023;11(12):3145. 10.3390/biomedicines11123145 PMC10740632 38137366

[60] Vonk LA, van Dooremalen SFJ, Liv N, Klumperman J, Coffer PJ, Saris DBF, Lorenowicz MJ. Mesenchymal stromal/stem cell-derived extracellular vesicles promote human cartilage regeneration in vitro. Theranostics. 2018;8(4):906–920. 29463990 10.7150/thno.20746 PMC5817101

[61] Mao G, Zhang Z, Hu S, Zhang Z, Chang Z, Huang Z, Liao W, Kang Y. Exosomes derived from miR-92a-3p-overexpressing human mesenchymal stem cells enhance chondrogenesis and suppress cartilage degradation via targeting WNT5A. Stem Cell Res Ther. 2018;9(1):247. 30257711 10.1186/s13287-018-1004-0 PMC6158854

[62] Zhou X, Liang H, Hu X, An J, Ding S, Yu S, Liu C, Li F, Xu Y. BMSC-derived exosomes from congenital polydactyly tissue alleviate osteoarthritis by promoting chondrocyte proliferation. Cell Death Discov. 2020;6(1):142. 33303743 10.1038/s41420-020-00374-z PMC7730395

[63] Li S, Stockl S, Lukas C, Gotz J, Herrmann M, Federlin M, Grässel S. hBMSC-derived extracellular vesicles attenuate IL-1β -induced catabolic effects on OA-chondrocytes by regulating pro-inflammatory signaling pathways. Front Bioeng Biotechnol. 2020;8: Article 603598. 33425869 10.3389/fbioe.2020.603598 PMC7793861

[64] Wang X, Li Z, Cui Y, Cui X, Chen C, Wang Z. Exosomes isolated from bone marrow mesenchymal stem cells exert a protective effect on osteoarthritis via lncRNA LYRM4-AS1-GRPR-miR-6515-5p. Front Cell Dev Biol. 2021;9: Article 644380. 34124036 10.3389/fcell.2021.644380 PMC8193855

[65] Liao Q, Li BJ, Li Y, Xiao Y, Zeng H, Liu JM, Yuan LX, Liu G. Low-intensity pulsed ultrasound promotes osteoarthritic cartilage regeneration by BMSC-derived exosomes via modulating the NF-κ B signaling pathway. Int Immunopharmacol. 2021;97: Article 107824. 34102487 10.1016/j.intimp.2021.107824

[66] Yang H, Cong M, Huang W, Chen J, Zhang M, Gu X, Sun C, Yang H. The effect of human bone marrow mesenchymal stem cell-derived exosomes on cartilage repair in rabbits. Stem Cells Int. 2022;2022:5760107. 36117721 10.1155/2022/5760107 PMC9477595

[67] Shen K, Duan A, Cheng J, Yuan T, Zhou J, Song H, Chen Z, Wan B, Liu J, Zhang X, et al. Exosomes derived from hypoxia preconditioned mesenchymal stem cells laden in a silk hydrogel promote cartilage regeneration via the miR-205-5p/PTEN/AKT pathway. Acta Biomater. 2022;143:173–188. 35202856 10.1016/j.actbio.2022.02.026

[68] Deng S, Zhu F, Dai K, Wang J, Liu C. Harvest of functional mesenchymal stem cells derived from in vivo osteo-organoids. Biomater Transl. 2023;4(4):270–279. 38282704 10.12336/biomatertransl.2023.04.006 PMC10817801

[69] Brett E, Tevlin R, McArdle A, Seo EY, Chan CKF, Wan DC, Longaker MT. Human adipose-derived stromal cell isolation methods and use in osteogenic and adipogenic in vivo applications. Curr Protoc Stem Cell Biol. 2017;43:2H.1.1–2H.1.15. 10.1002/cpsc.41 PMC6487849 29140567

[70] Tevlin R, desJardins-Park H, Huber J, DiIorio SE, Longaker MT, Wan DC. Musculoskeletal tissue engineering: Adipose derived stromal cell implementation for the treatment of osteoarthritis. Biomaterials. 2022;286: Article 121544. 35633592 10.1016/j.biomaterials.2022.121544 PMC9267037

[71] Guillen MI, Tofino-Vian M, Silvestre A, Castejon MA, Alcaraz MJ. Role of peroxiredoxin 6 in the chondroprotective effects of microvesicles from human adipose tissue-derived mesenchymal stem cells. J Orthop Translat. 2021;30:61–69. 34611515 10.1016/j.jot.2021.08.003 PMC8458778

[72] Wu J, Kuang L, Chen C, Yang J, Zeng WN, Li T, Chen H, Huang S, Fu Z, Li J, et al. miR-100-5p-abundant exosomes derived from infrapatellar fat pad MSCs protect articular cartilage and ameliorate gait abnormalities via inhibition of mTOR in osteoarthritis. Biomaterials. 2019;206:87–100. 30927715 10.1016/j.biomaterials.2019.03.022

[73] Tofino-Vian M, Guillen MI, Perez Del Caz MD, Silvestre A, Alcaraz MJ. Microvesicles from human adipose tissue-derived mesenchymal stem cells as a new protective strategy in osteoarthritic chondrocytes. Cell Physiol Biochem. 2018;47(1):11–25. 29763932 10.1159/000489739

[74] Woo CH, Kim HK, Jung GY, Jung YJ, Lee KS, Yun YE, Han J, Lee J, Kim WS, Choi JS, et al. Small extracellular vesicles from human adipose-derived stem cells attenuate cartilage degeneration. J Extracell Vesicles. 2020;9(1):1735249. 32284824 10.1080/20013078.2020.1735249 PMC7144299

[75] Cavallo C, Merli G, Borzi RM, Zini N, D’Adamo S, Guescini M, Grigolo B, Di Martino A, Santi S, Filardo G. Small extracellular vesicles from adipose derived stromal cells significantly attenuate in vitro the NF-κB dependent inflammatory/catabolic environment of osteoarthritis. Sci Rep. 2021;11(1):1053. 33441764 10.1038/s41598-020-80032-7 PMC7806716

[76] Li F, Xu Z, Xie Z, Sun X, Li C, Chen Y, Xu J, Pi G. Adipose mesenchymal stem cells-derived exosomes alleviate osteoarthritis by transporting microRNA -376c-3p and targeting the WNT-beta-catenin signaling axis. Apoptosis. 2023;28(3-4):362–378. 36396777 10.1007/s10495-022-01787-0

[77] Li Q, Yu H, Zhao F, Cao C, Wu T, Fan Y, Ao Y, Hu X. 3D printing of microenvironment-specific bioinspired and exosome-reinforced hydrogel scaffolds for efficient cartilage and subchondral bone regeneration. Adv Sci (Weinh). 2023;10(26): Article e2303650. 37424038 10.1002/advs.202303650 PMC10502685

[78] Liu Y, Zeng Y, Si HB, Tang L, Xie HQ, Shen B. Exosomes derived from human urine-derived stem cells overexpressing miR-140-5p alleviate knee osteoarthritis through downregulation of VEGFA in a rat model. Am J Sports Med. 2022;50(4):1088–1105. 35179989 10.1177/03635465221073991

[79] Liu X, Yang Y, Li Y, Niu X, Zhao B, Wang Y, Bao C, Xie Z, Lin Q, Zhu L. Integration of stem cell-derived exosomes with in situ hydrogel glue as a promising tissue patch for articular cartilage regeneration. Nanoscale. 2017;9(13):4430–4438. 28300264 10.1039/c7nr00352h

[80] Yang Y, Zhu Z, Gao R, Yuan J, Zhang J, Li H, Xie Z, Wang Y. Controlled release of MSC-derived small extracellular vesicles by an injectable Diels-Alder crosslinked hyaluronic acid/PEG hydrogel for osteoarthritis improvement. Acta Biomater. 2021;128:163–174. 33862283 10.1016/j.actbio.2021.04.003

[81] Liang Y, Xu X, Li X, Xiong J, Li B, Duan L, Wang D, Xia J. Chondrocyte-targeted microRNA delivery by engineered exosomes toward a cell-free osteoarthritis therapy. ACS Appl Mater Interfaces. 2020;12(33):36938–36947. 32814390 10.1021/acsami.0c10458

[82] Fang J, Wang X, Jiang W, Zhu Y, Hu Y, Zhao Y, Song X, Zhao J, Zhang W, Peng J, et al. Platelet-rich plasma therapy in the treatment of diseases associated with orthopedic injuries. Tissue Eng Part B Rev. 2020;26(6):571–585. 32380937 10.1089/ten.teb.2019.0292 PMC9208862

[83] Zhang Y, Wang X, Chen J, Qian D, Gao P, Qin T, Jiang T, Yi J, Xu T, Huang Y, et al. Exosomes derived from platelet-rich plasma administration in site mediate cartilage protection in subtalar osteoarthritis. J Nanobiotechnology. 2022;20(1):56. 35093078 10.1186/s12951-022-01245-8 PMC8801111

[84] Cosenza S, Toupet K, Maumus M, Luz-Crawford P, Blanc-Brude O, Jorgensen C, Noël D. Mesenchymal stem cells-derived exosomes are more immunosuppressive than microparticles in inflammatory arthritis. Theranostics. 2018;8(5):1399–1410. 29507629 10.7150/thno.21072 PMC5835945

[85] Huang Y, Zhang X, Zhan J, Yan Z, Chen D, Xue X, Pan X. Bone marrow mesenchymal stem cell-derived exosomal miR-206 promotes osteoblast proliferation and differentiation in osteoarthritis by reducing Elf3. J Cell Mol Med. 2021;25(16):7734–7745. 34160894 10.1111/jcmm.16654 PMC8358849

[86] Shen X, Qin J, Wei Z, Liu F. Bone marrow mesenchymal stem cell exosome-derived lncRNA TUC339 influences the progression of osteoarthritis by regulating synovial macrophage polarization and chondrocyte apoptosis. Biomed Pharmacother. 2023;167: Article 115488. 37729727 10.1016/j.biopha.2023.115488

[87] Pang L, Jin H, Lu Z, Xie F, Shen H, Li X, Zhang X, Jiang X, Wu L, Zhang M, et al. Treatment with mesenchymal stem cell-derived nanovesicle-containing gelatin methacryloyl hydrogels alleviates osteoarthritis by modulating chondrogenesis and macrophage polarization. Adv Healthc Mater. 2023;12(17): Article e2300315. 36848378 10.1002/adhm.202300315

[88] Zeng J, Sun P, Zhao Y, Fang X, Wu Z, Qi X. Bone mesenchymal stem cell-derived exosomes involved co-delivery and synergism effect with icariin via mussel-inspired multifunctional hydrogel for cartilage protection. Asian J Pharm Sci. 2023;18(3): Article 100799. 37274922 10.1016/j.ajps.2023.100799 PMC10238841

[89] Yin Z, Qin C, Pan S, Shi C, Wu G, Feng Y, Zhang J, Yu Z, Liang B, Gui J. Injectable hyperbranched PEG crosslinked hyaluronan hydrogel microparticles containing mir-99a-3p modified subcutaneous ADSCs-derived exosomes was beneficial for long-term treatment of osteoarthritis. Mater Today Bio. 2023;23: Article 100813. 10.1016/j.mtbio.2023.100813 PMC10562164 37822452

[90] Sun W, Qu S, Ji M, Sun Y, Hu B. BMP-7 modified exosomes derived from synovial mesenchymal stem cells attenuate osteoarthritis by M2 polarization of macrophages. Heliyon. 2023;9(9): Article e19934. 37809369 10.1016/j.heliyon.2023.e19934 PMC10559348

[91] Long L, Zou G, Cheng Y, Li F, Wu H, Shen Y. MATN3 delivered by exosome from synovial mesenchymal stem cells relieves knee osteoarthritis: Evidence from in vitro and in vivo studies. J Orthop Translat. 2023;41:20–32. 37635810 10.1016/j.jot.2023.06.003 PMC10448336

[92] Qiu M, Xie Y, Tan G, Wang X, Huang P, Hong L. Synovial mesenchymal stem cell-derived exosomal miR-485-3p relieves cartilage damage in osteoarthritis by targeting the NRP1-mediated PI3K/Akt pathway: Exosomal miR-485-3p relieves cartilage damage. Heliyon. 2024;10(2): Article e24042. 38293485 10.1016/j.heliyon.2024.e24042 PMC10826677

[93] Yang RZ, Zheng HL, Xu WN, Zheng XF, Li B, Jiang LS, Jiang SD. Vascular endothelial cell-secreted exosomes facilitate osteoarthritis pathogenesis by promoting chondrocyte apoptosis. Aging (Albany NY). 2021;13(3):4647–4662. 33526719 10.18632/aging.202506 PMC7906201

[94] Ebata T, Terkawi MA, Kitahara K, Yokota S, Shiota J, Nishida Y, Matsumae G, Alhasan H, Hamasaki M, Hontani K, et al. Noncanonical pyroptosis triggered by macrophage-derived extracellular vesicles in chondrocytes leading to cartilage catabolism in osteoarthritis. Arthritis Rheumatol. 2023;75(8):1358–1369. 36924130 10.1002/art.42505

[95] Wang R, Xu B, Xu H. TGF-β 1 promoted chondrocyte proliferation by regulating Sp1 through MSC-exosomes derived miR-135b. Cell Cycle. 2018;17(24):2756–2765. 30526325 10.1080/15384101.2018.1556063 PMC6343719

[96] Wang R, Xu B. TGF-beta1-modified MSC-derived exosomal miR-135b attenuates cartilage injury via promoting M2 synovial macrophage polarization by targeting MAPK6. Cell Tissue Res. 2021;384(1):113–127. 33404840 10.1007/s00441-020-03319-1

[97] Dong J, Li L, Fang X, Zang M. Exosome-encapsulated microRNA-127-3p released from bone marrow-derived mesenchymal stem cells alleviates osteoarthritis through regulating CDH11-mediated Wnt/β -catenin pathway. J Pain Res. 2021;14:297–310. 33574696 10.2147/JPR.S291472 PMC7871222

[98] He L, He T, Xing J, Zhou Q, Fan L, Liu C, Chen Y, Wu D, Tian Z, Liu B, et al. Bone marrow mesenchymal stem cell-derived exosomes protect cartilage damage and relieve knee osteoarthritis pain in a rat model of osteoarthritis. Stem Cell Res Ther. 2020;11(1):276. 32650828 10.1186/s13287-020-01781-w PMC7350730

[99] Zhang J, Rong Y, Luo C, Cui W. Bone marrow mesenchymal stem cell-derived exosomes prevent osteoarthritis by regulating synovial macrophage polarization. Aging (Albany NY). 2020;12(24):25138–25152. 33350983 10.18632/aging.104110 PMC7803581

[100] Jin Z, Ren J, Qi S. Exosomal miR-9-5p secreted by bone marrow-derived mesenchymal stem cells alleviates osteoarthritis by inhibiting syndecan-1. Cell Tissue Res. 2020;381(1):99–114. 32377874 10.1007/s00441-020-03193-x

[101] Wan J, He Z, Peng R, Wu X, Zhu Z, Cui J, Hao X, Chen A, Zhang J, Cheng P. Injectable photocrosslinking spherical hydrogel-encapsulated targeting peptide-modified engineered exosomes for osteoarthritis therapy. J Nanobiotechnology. 2023;21(1):284. 37605203 10.1186/s12951-023-02050-7 PMC10440922

[102] Guan P, Liu C, Xie D, Mao S, Ji Y, Lin Y, Chen Z, Wang Q, Fan L, Sun Y. Exosome-loaded extracellular matrix-mimic hydrogel with anti-inflammatory property facilitates/promotes growth plate injury repair. Bioact Mater. 2022;10:145–158. 34901536 10.1016/j.bioactmat.2021.09.010 PMC8637006

[103] Zhang FX, Liu P, Ding W, Meng QB, Su DH, Zhang QC, Lian RX, Yu BQ, Zhao MD, Dong J, et al. Injectable mussel-inspired highly adhesive hydrogel with exosomes for endogenous cell recruitment and cartilage defect regeneration. Biomaterials. 2021;278: Article 121169. 34626937 10.1016/j.biomaterials.2021.121169

[104] Zhou Y, Ming J, Li Y, Li B, Deng M, Ma Y, Chen Z, Zhang Y, Li J, Liu S. Exosomes derived from miR-126-3p-overexpressing synovial fibroblasts suppress chondrocyte inflammation and cartilage degradation in a rat model of osteoarthritis. Cell Death Discov. 2021;7(1):37. 33627637 10.1038/s41420-021-00418-y PMC7904758

[105] Sang X, Zhao X, Yan L, Jin X, Wang X, Wang J, Yin Z, Zhang Y, Meng Z. Thermosensitive hydrogel loaded with primary chondrocyte-derived exosomes promotes cartilage repair by regulating macrophage polarization in osteoarthritis. Tissue Eng Regen Med. 2022;19(3):629–642. 35435577 10.1007/s13770-022-00437-5 PMC9130414

[106] Jiang Y, Li J, Xue X, Yin Z, Xu K, Su J. Engineered extracellular vesicles for bone therapy. Nano Today. 2022;101487.

[107] Xu X, Li J, Lu Y, Shan Y, Shen Z, Sun F, Zhu J, Chen W, Shi H. Extracellular vesicles in the repair of bone and cartilage injury: From macro-delivery to micro-modification. Adv Ther. 2024;7(6): Article 2300428.

[108] Thomas BL, Eldridge SE, Nosrati B, Alvarez M, Thorup AS, Nalesso G, Caxaria S, Barawi A, Nicholson JG, Perretti M, et al. WNT3A-loaded exosomes enable cartilage repair. J Extracell Vesicles. 2021;10(7): Article e12088. 34025953 10.1002/jev2.12088 PMC8134720

[109] Yan W, Li Y, Xie S, Tao WA, Hu J, Liu H, Zhang G, Liu F, Nie Y, Chen X, et al. Chondrocyte-targeted delivery system of Sortase A-engineered extracellular vesicles silencing MMP13 for osteoarthritis therapy. Adv Healthc Mater. 2024;13(16): Article e2303510. 38545904 10.1002/adhm.202303510

[110] Feng K, Xie X, Yuan J, Gong L, Zhu Z, Zhang J, Li H, Yang Y, Wang Y. Reversing the surface charge of MSC-derived small extracellular vesicles by epsilonPL-PEG-DSPE for enhanced osteoarthritis treatment. J Extracell Vesicles. 2021;10(13): Article e12160. 34724347 10.1002/jev2.12160 PMC8559985

[111] Chen Y, Xue K, Zhang X, Zheng Z, Liu K. Exosomes derived from mature chondrocytes facilitate subcutaneous stable ectopic chondrogenesis of cartilage progenitor cells. Stem Cell Res Ther. 2018;9(1):318. 30463592 10.1186/s13287-018-1047-2 PMC6249792

[112] Qi H, Liu DP, Xiao DW, Tian DC, Su YW, Jin SF. Exosomes derived from mesenchymal stem cells inhibit mitochondrial dysfunction-induced apoptosis of chondrocytes via p38, ERK, and Akt pathways. In Vitro Cell Dev Biol Anim. 2019;55(3):203–210. 30783864 10.1007/s11626-019-00330-x

[113] Chen P, Zheng L, Wang Y, Tao M, Xie Z, Xia C, Gu C, Chen J, Qiu P, Mei S, et al. Desktop-stereolithography 3D printing of a radially oriented extracellular matrix/mesenchymal stem cell exosome bioink for osteochondral defect regeneration. Theranostics. 2019;9(9):2439–2459. 31131046 10.7150/thno.31017 PMC6525998

[114] Won Lee G, Thangavelu M, Joung Choi M, Yeong Shin E, Sol Kim H, Seon Baek J, Woon Jeong Y, Eun Song J, Carlomagno C, Miguel Oliveira J, et al. Exosome mediated transfer of miRNA-140 promotes enhanced chondrogenic differentiation of bone marrow stem cells for enhanced cartilage repair and regeneration. J Cell Biochem. 2020;121(7):3642–3652. 32091634 10.1002/jcb.29657

[115] Esmaeili A, Hosseini S, Kamali A, Hosseinzadeh M, Shekari F, Baghaban EM. Co-aggregation of MSC/chondrocyte in a dynamic 3D culture elevates the therapeutic effect of secreted extracellular vesicles on osteoarthritis in a rat model. Sci Rep. 2022;12(1):19827. 36400827 10.1038/s41598-022-22592-4 PMC9674636

[116] Wang D, Berg D, Ba H, Sun H, Wang Z, Li C. Deer antler stem cells are a novel type of cells that sustain full regeneration of a mammalian organ-deer antler. Cell Death Dis. 2019;10(6):443. 31165741 10.1038/s41419-019-1686-y PMC6549167

[117] Feleke M, Bennett S, Chen J, Hu X, Williams D, Xu J. New physiological insights into the phenomena of deer antler: A unique model for skeletal tissue regeneration. J Orthop Translat. 2021;27:57–66. 33437638 10.1016/j.jot.2020.10.012 PMC7773678

[118] Lei J, Jiang X, Li W, Ren J, Wang D, Ji Z, Wu Z, Cheng F, Cai Y, Yu ZR, et al. Exosomes from antler stem cells alleviate mesenchymal stem cell senescence and osteoarthritis. Protein Cell. 2022;13(3):220–226. 34342820 10.1007/s13238-021-00860-9 PMC8901817

[119] Zhou J, Zhao J, Wang Y, Jiang Y, Li X, Wang D, Yue Z, Lv J, Sun H. Repair of mechanical cartilage damage using exosomes derived from deer antler stem cells. Front Biosci (Landmark Ed). 2024;29(8):309. 39206920 10.31083/j.fbl2908309

[120] Guo R, Fan J. Extracellular vesicles derived from auricular chondrocytes facilitate cartilage differentiation of adipose-derived mesenchymal stem cells. Aesth Plast Surg. 2023;47(6):2823–2832. 10.1007/s00266-023-03292-4 36849663

[121] Chen X, Xing X, Lin S, Huang L, He L, Zou Y, Zhang X, Su B, Lu Y, Zheng D. Plant-derived nanovesicles: Harnessing nature’s power for tissue protection and repair. J Nanobiotechnology. 2023;21(1):445. 38001440 10.1186/s12951-023-02193-7 PMC10668476

[122] Xu Z, Xu Y, Zhang K, Liu Y, Liang Q, Thakur A, Liu W, Yan Y. Plant-derived extracellular vesicles (PDEVs) in nanomedicine for human disease and therapeutic modalities. J Nanobiotechnology. 2023;21(1):114. 36978093 10.1186/s12951-023-01858-7 PMC10049910

[123] Jensen WA. The composition and ultrastructure of the nucellus in cotton. J Ultrastruct Res. 1965;13(1-2):112–128.

[124] Halperin W, Jensen WA. Ultrastructural changes during growth and embryogenesis in carrot cell cultures. J Ultrastruct Res. 1967;18(3):428–443. 6025110 10.1016/s0022-5320(67)80128-x

[125] Lian MQ, Chng WH, Liang J, Yeo HQ, Lee CK, Belaid M, Tollemeto M, Wacker MG, Czarny B, Pastorin G. Plant-derived extracellular vesicles: Recent advancements and current challenges on their use for biomedical applications. J Extracell Vesicles. 2022;11(12): Article e12283. 36519808 10.1002/jev2.12283 PMC9753580

[126] Feng J, Xiu Q, Huang Y, Troyer Z, Li B, Zheng L. Plant-derived vesicle-like nanoparticles as promising biotherapeutic tools: Present and future. Adv Mater. 2023;35(24): Article e2207826. 36592157 10.1002/adma.202207826

[127] Hwang JH, Park YS, Kim HS, Kim DH, Lee SH, Lee CH, Lee SH, Kim JE, Lee S, Kim HM, et al. Yam-derived exosome-like nanovesicles stimulate osteoblast formation and prevent osteoporosis in mice. J Control Release. 2023;355:184–198. 36736431 10.1016/j.jconrel.2023.01.071

[128] Lo KJ, Wang MH, Ho CT, Pan MH. Plant-derived extracellular vesicles: A new Revolutionization of modern healthy diets and biomedical applications. J Agric Food Chem. 2024;72(6):2853–2878. 38300835 10.1021/acs.jafc.3c06867

[129] Urzi O, Gasparro R, Ganji NR, Alessandro R, Raimondo S. Plant-RNA in extracellular vesicles: The secret of cross-kingdom communication. Membranes (Basel). 2022;12(4):352. 35448322 10.3390/membranes12040352 PMC9028404

[130] Dad HA, Gu TW, Zhu AQ, Huang LQ, Peng LH. Plant exosome-like nanovesicles: Emerging therapeutics and drug delivery nanoplatforms. Mol Ther. 2021;29(1):13–31. 33278566 10.1016/j.ymthe.2020.11.030 PMC7791080

[131] Yıldırım M, Unsal N, Kabatas B, Eren O, Sahin F. Effect of Solanum lycopersicum and Citrus limon-derived exosome-like vesicles on chondrogenic differentiation of adipose-derived stem cells. Appl Biochem Biotechnol. 2024;196(1):203–219. 37103740 10.1007/s12010-023-04491-0

[132] Toyofuku M, Schild S, Kaparakis-Liaskos M, Eberl L. Composition and functions of bacterial membrane vesicles. Nat Rev Microbiol. 2023;21(7):415–430. 36932221 10.1038/s41579-023-00875-5

[133] Nandakumar KS, Fang Q, Wingbro Agren I, Bejmo ZF. Aberrant activation of immune and non-immune cells contributes to joint inflammation and bone degradation in rheumatoid arthritis. Int J Mol Sci. 2023;24(21):15883. 37958864 10.3390/ijms242115883 PMC10648236

[134] Liu Q, Hao H, Li J, Zheng T, Yao Y, Tian X, Zhang Z, Yi H. Oral administration of bovine milk-derived extracellular vesicles attenuates cartilage degeneration via modulating gut microbiota in DMM-induced mice. Nutrients. 2023;15(3):747. 36771453 10.3390/nu15030747 PMC9920331

[135] Dong X, Liu Y, Yang X, Li T. Extracellular vesicle miRNAs as key mediators in diet-gut microbiome-host interplay. Trends Food Sci Technol. 2023;136:268–281.

[136] Wang T, Mo L, Ou J, Fang Q, Wu H, Wu Y, Nandakumar KS. Proteus mirabilis vesicles induce mitochondrial apoptosis by regulating miR96-5p/Abca1 to inhibit osteoclastogenesis and bone loss. Front Immunol. 2022;13: Article 833040. 35242136 10.3389/fimmu.2022.833040 PMC8885728

[137] Liu J, Wu X, Lu J, Huang G, Dang L, Zhang H, Zhong C, Zhang Z, Li D, Li F, et al. Exosomal transfer of osteoclast-derived miRNAs to chondrocytes contributes to osteoarthritis progression. Nat Aging. 2021;1(4):368–384. 37117596 10.1038/s43587-021-00050-6

[138] Cheung KCP, Jiao M, Xingxuan C, Wei J. Extracellular vesicles derived from host and gut microbiota as promising nanocarriers for targeted therapy in osteoporosis and osteoarthritis. Front Pharmacol. 2022;13:1051134. 36686680 10.3389/fphar.2022.1051134 PMC9859449

[139] Li Z, Huang Z, Bai L. Cell interplay in osteoarthritis. Front Cell Dev Biol. 2021;9: Article 720477. 34414194 10.3389/fcell.2021.720477 PMC8369508

[140] He L, Chen Y, Ke Z, Pang M, Yang B, Feng F, Wu Z, Liu C, Liu B, Zheng X, et al. Exosomes derived from miRNA-210 overexpressing bone marrow mesenchymal stem cells protect lipopolysaccharide induced chondrocytes injury via the NF-κ B pathway. Gene. 2020;751: Article 144764. 32428694 10.1016/j.gene.2020.144764

[141] Saari H, Lisitsyna E, Rautaniemi K, Rojalin T, Niemi L, Nivaro O, Laaksonen T, Yliperttula M, Vuorimaa-Laukkanen E. FLIM reveals alternative EV-mediated cellular up-take pathways of paclitaxel. J Control Release. 2018;284:133–143. 29906554 10.1016/j.jconrel.2018.06.015

[142] Witwer KW, Van Balkom BWM, Bruno S, Choo A, Dominici M, Gimona M, Hill AF, Kleijn D, Koh M, Lai RC, et al. Defining mesenchymal stromal cell (MSC)-derived small extracellular vesicles for therapeutic applications. J Extracell Vesicles. 2019;8(1):1609206. 31069028 10.1080/20013078.2019.1609206 PMC6493293

[143] Gimona M, Brizzi MF, Choo ABH, Dominici M, Davidson SM, Grillari J, Hermann DM, Hill AF, de Kleijn D, Lai RC, et al. Critical considerations for the development of potency tests for therapeutic applications of mesenchymal stromal cell-derived small extracellular vesicles. Cytotherapy. 2021;23(5):373–380. 33934807 10.1016/j.jcyt.2021.01.001

[144] Li Q, Yu H, Sun M, Yang P, Hu X, Ao Y, Cheng J. The tissue origin effect of extracellular vesicles on cartilage and bone regeneration. Acta Biomater. 2021;125:253–266. 33657452 10.1016/j.actbio.2021.02.039

[145] Semerci Sevimli T, Sevimli M, Qomi Ekenel E, Altug Tasa B, Nur Soykan M, Demir Gucluer Z, İnan U, Uysal O, Bağış SG, Çemrek F, et al. Comparison of exosomes secreted by synovial fluid-derived mesenchymal stem cells and adipose tissue-derived mesenchymal stem cells in culture for microRNA-127-5p expression during chondrogenesis. Gene. 2023;865: Article 147337. 36878417 10.1016/j.gene.2023.147337

[146] Dordevic M, Dc TA, Do JE. IRB approved pilot safety study of an extracellular vesicle isolate product evaluating the treatment of osteoarthritis in combat-related injuries. J Stem Cell Res. 2020;1(2):1–10.

