# Supplementary material for: From Bench to Bedside: The Role of Extracellular Vesicles in Cartilage Injury Treatment
Source: Biomater Res. 2024 Nov 22;28:0110. doi: 10.34133/bmr.0110 (PMC11582190; doi:10.34133/bmr.0110)
Supplement: Supplementary 1 — Figs. S1 to S4 Tables S1 and S2 [file bmr.0110.f1.zip › BMR_COI_Form-all.pdf]

Biomaterials Research
A SCIENCE PARTNER JOURNAL

Authorship Form and Statement of Conflicts of Interest

Author Name: Pan Jin

Manuscript Number: BMR-D-24-00251

Manuscript Title: From Bench to Bedside: The Role of Extracellular Vesicles in Cartilage Injury Treatment

Each author must complete the following form prior to acceptance of their paper:

I. Authorship:

The authorship policies of *Biomaterials Research* follow those recommended by the report "On Being a Scientist", 3rd Edition, published by the US National Academy of Sciences (http://www.nap.edu/catalog/12192.html). In particular, we note that "just providing laboratory space for a project or furnishing a sample used in the research is not sufficient to be included as an author, although such contributions... may be recognized in a separate acknowledgment section."

In order to meet our requirements for authorship of a paper, you must have participated significantly in the reported research or writing of the paper. Please affirm that you meet these criteria by indicating your contribution to all of the following descriptions (from 0 to 100%):

**Authorship Activity**	Level of participation					
	0%	20%	40%	60%	80%	100%
Participated in the design and/or interpretation of the reported experiments or results.	○	○	○	○	○	◉
Participated in the acquisition and/or analysis of data.<br>Indicate which data: ______	◉	○	○	○	○	○
Participated in drafting and/or revising the manuscript.	○	○	○	○	○	◉
Primarily responsible for a particular, specialized role in the research (e.g. statistical analysis, crystallography, preparation of cell lines, etc)<br>Briefly state roles: ______	◉	○	○	○	○	○
Provided administrative, technical or supervisory support.	○	○	○	○	◉	○

The senior author from each lab or group must answer this question: I have personally checked all the original data that was generated by my lab or group:

☑ Yes ☐ Not applicable; I am not the senior author or lab head.

If yes, these data are presented in these figures and tables (including the Supporting Online Material):

I have reviewed, or will review, the revised manuscript and approve of its submission to *Biomaterials Research* for publication.

☑ Yes ☐ No

If my university or institution has a separate publication license that applies to me (as required by Harvard, MIT, Open University, *et al.*) I have applied for a waiver.

☐ Yes ☑ There are no publication policies or restrictions limiting my ability to grant exclusive publication rights to The Korean Society for Biomaterials ("KSBM").

II. Data sharing:

I affirm that all data necessary for a reader of *Biomaterials Research* to understand and evaluate the conclusions of the paper will be archived in an approved database and made available to any reader.

☑ Yes ☐ No

III. Materials sharing:

I understand that after publication, all reasonable requests for materials and data must be fulfilled.

☐ Yes ☑ No

Biomaterials Research must be informed of any restrictions on sharing of materials [Materials Transfer Agreements (MTAs) or patents, for example] applying to materials used in the reported research.

☑ No, there are no MTAs

☐ Yes. Information on MTAs is described below.

IV. Conflict of Interest:

Biomaterials Research has a primary responsibility to its readers and to the public to provide in its pages clear and unbiased scientific results and analyses. We think that our readers should be informed of additional relationships of our authors that could pose a conflict of interest. Thus, for readers to evaluate the data and opinions presented in this journal, they must be informed of financial and other interests of our authors that may be at odds with unbiased presentation of data or analysis.

Therefore, *Biomaterials Research* believes that manuscripts should be accompanied by clear disclosures from all authors of their affiliations, funding sources, or financial holdings that might raise questions about possible sources of bias.
Disclosure is accomplished in three ways:

First, by a complete listing of the current institutional affiliations of the authors.

This list must include academic as well as corporate and other industrial affiliations. As the editors deem appropriate, items in this list will be included in the author affiliations printed in the manuscript. Please indicate below:
☑ Yes, all my affiliations are listed on the title page of the paper.

Additional affiliations not on the title page are:

Second, through the acknowledgment of all financial contributions to the work being reported, including contributions "in kind." All funding sources will be listed in the published manuscript. Please indicate below:

☑ All my funding sources for this study are listed in the acknowledgement section of the paper.
☐ Additional funding sources not noted in the manuscript are:

Third, through the execution of a statement disclosing to the Editors all financial holdings, professional affiliations, advisory positions, board memberships, patent holdings and the like that might bear a relationship to the subject matter of the contribution. The Editors will determine whether the material disclosed to them should be published as part of the article.

The following are declarable relationships: **Financial**: Significant financial interest (equity holdings or stock options) in any corporate entity dealing with the material or the subject matter of this contribution. Please disclose the entity and the nature and amount of the holding:

☑ None

☐ I have a financial relationship, as described below.

Management/Advisory affiliations: Within the last 3 years, status as an officer, a member of the Board, or a member of an Advisory Committee of any entity engaged in activity related to the subject matter of this contribution. Please disclose the nature of these relationships and the financial arrangements.

☑ None

☐ I have a management/advisory relationship, as described below:

Paid Consulting: Within the last 3 years, receipt of consulting fees, honoraria, speaking fees, or expert testimony fees from entities that have a financial interest in the results and materials of this study. Please enumerate.

☑ None

☐ I have a consulting relationship, as described below:

Patents: A planned, pending, or awarded patent on this work by any of the authors or their institutions. Please explain.

☑ None

☐ I or my institution has a patent related to this work, as described below

Declaration: I declare that I have read *Biomaterials Research's* full Conflict of Interest Policy and have disclosed all declarable relationships as defined therein, if any.

This form was submitted on 09/20/2024

Name Pan Jin

Signature Pan Jin

Biomaterials Research
A SCIENCE PARTNER JOURNAL

Authorship Form and Statement of Conflicts of Interest

Author Name: Huan Liu

Manuscript Number: BMR-D-24-00251

Manuscript Title: From Bench to Bedside: The Role of Extracellular Vesicles in Cartilage Injury Treatment

Each author must complete the following form prior to acceptance of their paper:

I. Authorship:

The authorship policies of *Biomaterials Research* follow those recommended by the report "On Being a Scientist", 3rd Edition, published by the US National Academy of Sciences (http://www.nap.edu/catalog/12192.html). In particular, we note that "just providing laboratory space for a project or furnishing a sample used in the research is not sufficient to be included as an author, although such contributions... may be recognized in a separate acknowledgment section."

In order to meet our requirements for authorship of a paper, you must have participated significantly in the reported research or writing of the paper. Please affirm that you meet these criteria by indicating your contribution to all of the following descriptions (from 0 to 100%):

Authorship Activity	Level of participation					
	0%	20%	40%	60%	80%	100%
Participated in the design and/or interpretation of the reported experiments or results.	○	○	○	●	○	○
Participated in the acquisition and/or analysis of data.<br>Indicate which data: ______	●	○	○	○	○	○
Participated in drafting and/or revising the manuscript.	○	○	○	○	●	○
Primarily responsible for a particular, specialized role in the research (e.g. statistical analysis, crystallography, preparation of cell lines, etc)<br>Briefly state roles: ______	●	○	○	○	○	○
Provided administrative, technical or supervisory support.	●	○	○	○	○	○

The senior author from each lab or group must answer this question: I have personally checked all the original data that was generated by my lab or group:

☑ Yes ☐ Not applicable; I am not the senior author or lab head.

If yes, these data are presented in these figures and tables (including the Supporting Online Material):

I have reviewed, or will review, the revised manuscript and approve of its submission to *Biomaterials Research* for publication.

☑ Yes ☐ No

If my university or institution has a separate publication license that applies to me (as required by Harvard, MIT, Open University, *et al.*) I have applied for a waiver.

☐ Yes ☑ There are no publication policies or restrictions limiting my ability to grant exclusive publication rights to The Korean Society for Biomaterials ("KSBM").

II. Data sharing:

I affirm that all data necessary for a reader of *Biomaterials Research* to understand and evaluate the conclusions of the paper will be archived in an approved database and made available to any reader.

☑ Yes ☐ No

III. Materials sharing:

I understand that after publication, all reasonable requests for materials and data must be fulfilled.

☐ Yes ☑ No

Biomaterials Research must be informed of any restrictions on sharing of materials [Materials Transfer Agreements (MTAs) or patents, for example] applying to materials used in the reported research.

☑ No, there are no MTAs

☐ Yes. Information on MTAs is described below.

IV. Conflict of Interest:

Biomaterials Research has a primary responsibility to its readers and to the public to provide in its pages clear and unbiased scientific results and analyses. We think that our readers should be informed of additional relationships of our authors that could pose a conflict of interest. Thus, for readers to evaluate the data and opinions presented in this journal, they must be informed of financial and other interests of our authors that may be at odds with unbiased presentation of data or analysis.

Therefore, *Biomaterials Research* believes that manuscripts should be accompanied by clear disclosures from all authors of their affiliations, funding sources, or financial holdings that might raise questions about possible sources of bias.
Disclosure is accomplished in three ways:

First, by a complete listing of the current institutional affiliations of the authors.

This list must include academic as well as corporate and other industrial affiliations. As the editors deem appropriate, items in this list will be included in the author affiliations printed in the manuscript. Please indicate below:
☑ Yes, all my affiliations are listed on the title page of the paper.

Additional affiliations not on the title page are:

Second, through the acknowledgment of all financial contributions to the work being reported, including contributions "in kind." All funding sources will be listed in the published manuscript. Please indicate below:

☑ All my funding sources for this study are listed in the acknowledgement section of the paper.
☐ Additional funding sources not noted in the manuscript are:

Third, through the execution of a statement disclosing to the Editors all financial holdings, professional affiliations, advisory positions, board memberships, patent holdings and the like that might bear a relationship to the subject matter of the contribution. The Editors will determine whether the material disclosed to them should be published as part of the article.

The following are declarable relationships: **Financial**: Significant financial interest (equity holdings or stock options) in any corporate entity dealing with the material or the subject matter of this contribution. Please disclose the entity and the nature and amount of the holding:

☑ None

☐ I have a financial relationship, as described below.

Management/Advisory affiliations: Within the last 3 years, status as an officer, a member of the Board, or a member of an Advisory Committee of any entity engaged in activity related to the subject matter of this contribution. Please disclose the nature of these relationships and the financial arrangements.

☑ None

☐ I have a management/advisory relationship, as described below:

Paid Consulting: Within the last 3 years, receipt of consulting fees, honoraria, speaking fees, or expert testimony fees from entities that have a financial interest in the results and materials of this study. Please enumerate.

☑ None

☐ I have a consulting relationship, as described below:

Patents: A planned, pending, or awarded patent on this work by any of the authors or their institutions. Please explain.

☑ None

☐ I or my institution has a patent related to this work, as described below

Declaration: I declare that I have read *Biomaterials Research's* full Conflict of Interest Policy and have disclosed all declarable relationships as defined therein, if any.

This form was submitted on 09/20/2024

Name Huan Liu

Signature Huan Liu

Biomaterials Research
A SCIENCE PARTNER JOURNAL

Authorship Form and Statement of Conflicts of Interest

Author Name: Xichi chen

Manuscript Number: BMR-D-24-00251

Manuscript Title: From Bench to Bedside: The Role of Extracellular Vesicles in Cartilage Injury Treatment

Each author must complete the following form prior to acceptance of their paper:

I. Authorship:

The authorship policies of *Biomaterials Research* follow those recommended by the report "On Being a Scientist", 3rd Edition, published by the US National Academy of Sciences (http://www.nap.edu/catalog/12192.html). In particular, we note that "just providing laboratory space for a project or furnishing a sample used in the research is not sufficient to be included as an author, although such contributions... may be recognized in a separate acknowledgment section."

In order to meet our requirements for authorship of a paper, you must have participated significantly in the reported research or writing of the paper. Please affirm that you meet these criteria by indicating your contribution to all of the following descriptions (from 0 to 100%):

Authorship Activity	Level of participation					
	0%	20%	40%	60%	80%	100%
Participated in the design and/or interpretation of the reported experiments or results.	○	●	○	○	○	○
Participated in the acquisition and/or analysis of data.<br>Indicate which data: ____	●	○	○	○	○	○
Participated in drafting and/or revising the manuscript.	○	●	○	○	○	○
Primarily responsible for a particular, specialized role in the research (e.g. statistical analysis, crystallography, preparation of cell lines, etc)<br>Briefly state roles: ____	●	○	○	○	○	○
Provided administrative, technical or supervisory support.	●	○	○	○	○	○

The senior author from each lab or group must answer this question: I have personally checked all the original data that was generated by my lab or group:

☑ Yes ☐ Not applicable; I am not the senior author or lab head.

If yes, these data are presented in these figures and tables (including the Supporting Online Material):

I have reviewed, or will review, the revised manuscript and approve of its submission to *Biomaterials Research* for publication.

☑ Yes ☐ No

If my university or institution has a separate publication license that applies to me (as required by Harvard, MIT, Open University, *et al.*) I have applied for a waiver.

☐ Yes ☑ There are no publication policies or restrictions limiting my ability to grant exclusive publication rights to The Korean Society for Biomaterials ("KSBM").

II. Data sharing:

I affirm that all data necessary for a reader of *Biomaterials Research* to understand and evaluate the conclusions of the paper will be archived in an approved database and made available to any reader.

☑ Yes ☐ No

III. Materials sharing:

I understand that after publication, all reasonable requests for materials and data must be fulfilled.

☐ Yes ☑ No

Biomaterials Research must be informed of any restrictions on sharing of materials [Materials Transfer Agreements (MTAs) or patents, for example] applying to materials used in the reported research.

☑ No, there are no MTAs

☐ Yes. Information on MTAs is described below.

IV. Conflict of Interest:

Biomaterials Research has a primary responsibility to its readers and to the public to provide in its pages clear and unbiased scientific results and analyses. We think that our readers should be informed of additional relationships of our authors that could pose a conflict of interest. Thus, for readers to evaluate the data and opinions presented in this journal, they must be informed of financial and other interests of our authors that may be at odds with unbiased presentation of data or analysis.

Therefore, *Biomaterials Research* believes that manuscripts should be accompanied by clear disclosures from all authors of their affiliations, funding sources, or financial holdings that might raise questions about possible sources of bias.
Disclosure is accomplished in three ways:

First, by a complete listing of the current institutional affiliations of the authors.

This list must include academic as well as corporate and other industrial affiliations. As the editors deem appropriate, items in this list will be included in the author affiliations printed in the manuscript. Please indicate below:
☑ Yes, all my affiliations are listed on the title page of the paper.

Additional affiliations not on the title page are:

Second, through the acknowledgment of all financial contributions to the work being reported, including contributions "in kind." All funding sources will be listed in the published manuscript. Please indicate below:

☑ All my funding sources for this study are listed in the acknowledgement section of the paper.
☐ Additional funding sources not noted in the manuscript are:

Third, through the execution of a statement disclosing to the Editors all financial holdings, professional affiliations, advisory positions, board memberships, patent holdings and the like that might bear a relationship to the subject matter of the contribution. The Editors will determine whether the material disclosed to them should be published as part of the article.

The following are declarable relationships: **Financial**: Significant financial interest (equity holdings or stock options) in any corporate entity dealing with the material or the subject matter of this contribution. Please disclose the entity and the nature and amount of the holding:

☑ None

☐ I have a financial relationship, as described below.

Management/Advisory affiliations: Within the last 3 years, status as an officer, a member of the Board, or a member of an Advisory Committee of any entity engaged in activity related to the subject matter of this contribution. Please disclose the nature of these relationships and the financial arrangements.

☑ None

☐ I have a management/advisory relationship, as described below:

Paid Consulting: Within the last 3 years, receipt of consulting fees, honoraria, speaking fees, or expert testimony fees from entities that have a financial interest in the results and materials of this study. Please enumerate.

☑ None

☐ I have a consulting relationship, as described below:

Patents: A planned, pending, or awarded patent on this work by any of the authors or their institutions. Please explain.

☑ None

☐ I or my institution has a patent related to this work, as described below

Declaration: I declare that I have read *Biomaterials Research's* full Conflict of Interest Policy and have disclosed all declarable relationships as defined therein, if any.

This form was submitted on 09/20/2024

Name Xichi chen

Signature Xi chi chen,

Biomaterials Research
A SCIENCE PARTNER JOURNAL

Authorship Form and Statement of Conflicts of Interest

Author Name: Wei Liu

Manuscript Number: BMR-D-24-00251

Manuscript Title: From Bench to Bedside: The Role of Extracellular Vesicles in Cartilage Injury Treatment

Each author must complete the following form prior to acceptance of their paper:

I. Authorship:

The authorship policies of *Biomaterials Research* follow those recommended by the report "On Being a Scientist", 3rd Edition, published by the US National Academy of Sciences (http://www.nap.edu/catalog/12192.html). In particular, we note that "just providing laboratory space for a project or furnishing a sample used in the research is not sufficient to be included as an author, although such contributions... may be recognized in a separate acknowledgment section."

In order to meet our requirements for authorship of a paper, you must have participated significantly in the reported research or writing of the paper. Please affirm that you meet these criteria by indicating your contribution to all of the following descriptions (from 0 to 100%):

**Authorship Activity**	**Level of participation**					
	0%	20%	40%	60%	80%	100%
Participated in the design and/or interpretation of the reported experiments or results.	○	●	○	○	○	○
Participated in the acquisition and/or analysis of data.<br>Indicate which data: ____	●	○	○	○	○	○
Participated in drafting and/or revising the manuscript.	○	○	●	○	○	○
Primarily responsible for a particular, specialized role in the research (e.g. statistical analysis, crystallography, preparation of cell lines, etc)<br>Briefly state roles: ____	●	○	○	○	○	○
Provided administrative, technical or supervisory support.	●	○	○	○	○	○

The senior author from each lab or group must answer this question: I have personally checked all the original data that was generated by my lab or group:

☑ Yes ☐ Not applicable; I am not the senior author or lab head.

If yes, these data are presented in these figures and tables (including the Supporting Online Material):

I have reviewed, or will review, the revised manuscript and approve of its submission to *Biomaterials Research* for publication.

☑ Yes ☐ No

If my university or institution has a separate publication license that applies to me (as required by Harvard, MIT, Open University, *et al.*) I have applied for a waiver.

☐ Yes ☑ There are no publication policies or restrictions limiting my ability to grant exclusive publication rights to The Korean Society for Biomaterials ("KSBM").

II. Data sharing:

I affirm that all data necessary for a reader of *Biomaterials Research* to understand and evaluate the conclusions of the paper will be archived in an approved database and made available to any reader.

☑ Yes ☐ No

III. Materials sharing:

I understand that after publication, all reasonable requests for materials and data must be fulfilled.

☐ Yes ☑ No

Biomaterials Research must be informed of any restrictions on sharing of materials [Materials Transfer Agreements (MTAs) or patents, for example] applying to materials used in the reported research.

☑ No, there are no MTAs

☐ Yes. Information on MTAs is described below.

IV. Conflict of Interest:

Biomaterials Research has a primary responsibility to its readers and to the public to provide in its pages clear and unbiased scientific results and analyses. We think that our readers should be informed of additional relationships of our authors that could pose a conflict of interest. Thus, for readers to evaluate the data and opinions presented in this journal, they must be informed of financial and other interests of our authors that may be at odds with unbiased presentation of data or analysis.

Therefore, *Biomaterials Research* believes that manuscripts should be accompanied by clear disclosures from all authors of their affiliations, funding sources, or financial holdings that might raise questions about possible sources of bias.
Disclosure is accomplished in three ways:

First, by a complete listing of the current institutional affiliations of the authors.

This list must include academic as well as corporate and other industrial affiliations. As the editors deem appropriate, items in this list will be included in the author affiliations printed in the manuscript. Please indicate below:
[✔] Yes, all my affiliations are listed on the title page of the paper.

Additional affiliations not on the title page are:

Second, through the acknowledgment of all financial contributions to the work being reported, including contributions "in kind." All funding sources will be listed in the published manuscript. Please indicate below:

[✔] All my funding sources for this study are listed in the acknowledgement section of the paper.
[ ] Additional funding sources not noted in the manuscript are:

Third, through the execution of a statement disclosing to the Editors all financial holdings, professional affiliations, advisory positions, board memberships, patent holdings and the like that might bear a relationship to the subject matter of the contribution. The Editors will determine whether the material disclosed to them should be published as part of the article.

The following are declarable relationships: **Financial**: Significant financial interest (equity holdings or stock options) in any corporate entity dealing with the material or the subject matter of this contribution. Please disclose the entity and the nature and amount of the holding:

☑ None

☐ I have a financial relationship, as described below.

Management/Advisory affiliations: Within the last 3 years, status as an officer, a member of the Board, or a member of an Advisory Committee of any entity engaged in activity related to the subject matter of this contribution. Please disclose the nature of these relationships and the financial arrangements.

☑ None

☐ I have a management/advisory relationship, as described below:

Paid Consulting: Within the last 3 years, receipt of consulting fees, honoraria, speaking fees, or expert testimony fees from entities that have a financial interest in the results and materials of this study. Please enumerate.

☑ None

☐ I have a consulting relationship, as described below:

Patents: A planned, pending, or awarded patent on this work by any of the authors or their institutions. Please explain.

☑ None

☐ I or my institution has a patent related to this work, as described below

Declaration: I declare that I have read *Biomaterials Research's* full Conflict of Interest Policy and have disclosed all declarable relationships as defined therein, if any.

This form was submitted on 09/20/2024

Name Wei Liu

Signature Wei Liu

Biomaterials Research
A SCIENCE PARTNER JOURNAL

Authorship Form and Statement of Conflicts of Interest

Author Name: Tongmeng Jiang

Manuscript Number: BMR-D-24-00251

Manuscript Title: From Bench to Bedside: The Role of Extracellular Vesicles in Cartilage Injury Treatment

Each author must complete the following form prior to acceptance of their paper:

I. Authorship:

The authorship policies of *Biomaterials Research* follow those recommended by the report "On Being a Scientist", 3rd Edition, published by the US National Academy of Sciences (http://www.nap.edu/catalog/12192.html). In particular, we note that "just providing laboratory space for a project or furnishing a sample used in the research is not sufficient to be included as an author, although such contributions... may be recognized in a separate acknowledgment section."

In order to meet our requirements for authorship of a paper, you must have participated significantly in the reported research or writing of the paper. Please affirm that you meet these criteria by indicating your contribution to all of the following descriptions (from 0 to 100%):

Authorship Activity	Level of participation					
	0%	20%	40%	60%	80%	100%
Participated in the design and/or interpretation of the reported experiments or results.	○	○	○	○	○	●
Participated in the acquisition and/or analysis of data.<br><br>Indicate which data: ______	●	○	○	○	○	○
Participated in drafting and/or revising the manuscript.	○	○	○	○	○	●
Primarily responsible for a particular, specialized role in the research (e.g. statistical analysis, crystallography, preparation of cell lines, etc)<br><br>Briefly state roles: ______	●	○	○	○	○	○
Provided administrative, technical or supervisory support.	○	○	○	○	○	●

The senior author from each lab or group must answer this question: I have personally checked all the original data that was generated by my lab or group:

☑ Yes ☐ Not applicable; I am not the senior author or lab head.

If yes, these data are presented in these figures and tables (including the Supporting Online Material):

This is a review article.

I have reviewed, or will review, the revised manuscript and approve of its submission to *Biomaterials Research* for publication.

☑ Yes ☐ No

If my university or institution has a separate publication license that applies to me (as required by Harvard, MIT, Open University, *et al.*) I have applied for a waiver.

☐ Yes ☑ There are no publication policies or restrictions limiting my ability to grant exclusive publication rights to The Korean Society for Biomaterials ("KSBM").

II. Data sharing:

I affirm that all data necessary for a reader of *Biomaterials Research* to understand and evaluate the conclusions of the paper will be archived in an approved database and made available to any reader.

☑ Yes ☐ No

III. Materials sharing:

I understand that after publication, all reasonable requests for materials and data must be fulfilled.

☐ Yes ☑ No

Biomaterials Research must be informed of any restrictions on sharing of materials [Materials Transfer Agreements (MTAs) or patents, for example] applying to materials used in the reported research.

☑ No, there are no MTAs

☐ Yes. Information on MTAs is described below.

IV. Conflict of Interest:

Biomaterials Research has a primary responsibility to its readers and to the public to provide in its pages clear and unbiased scientific results and analyses. We think that our readers should be informed of additional relationships of our authors that could pose a conflict of interest. Thus, for readers to evaluate the data and opinions presented in this journal, they must be informed of financial and other interests of our authors that may be at odds with unbiased presentation of data or analysis.

Therefore, *Biomaterials Research* believes that manuscripts should be accompanied by clear disclosures from all authors of their affiliations, funding sources, or financial holdings that might raise questions about possible sources of bias.
Disclosure is accomplished in three ways:

First, by a complete listing of the current institutional affiliations of the authors.

This list must include academic as well as corporate and other industrial affiliations. As the editors deem appropriate, items in this list will be included in the author affiliations printed in the manuscript. Please indicate below:
☑ Yes, all my affiliations are listed on the title page of the paper.

Additional affiliations not on the title page are:

Second, through the acknowledgment of all financial contributions to the work being reported, including contributions "in kind." All funding sources will be listed in the published manuscript. Please indicate below:

☑ All my funding sources for this study are listed in the acknowledgement section of the paper.
☐ Additional funding sources not noted in the manuscript are:

Third, through the execution of a statement disclosing to the Editors all financial holdings, professional affiliations, advisory positions, board memberships, patent holdings and the like that might bear a relationship to the subject matter of the contribution. The Editors will determine whether the material disclosed to them should be published as part of the article.

The following are declarable relationships: **Financial**: Significant financial interest (equity holdings or stock options) in any corporate entity dealing with the material or the subject matter of this contribution. Please disclose the entity and the nature and amount of the holding:

☑ None

☐ I have a financial relationship, as described below.

Management/Advisory affiliations: Within the last 3 years, status as an officer, a member of the Board, or a member of an Advisory Committee of any entity engaged in activity related to the subject matter of this contribution. Please disclose the nature of these relationships and the financial arrangements.

☑ None

☐ I have a management/advisory relationship, as described below:

Paid Consulting: Within the last 3 years, receipt of consulting fees, honoraria, speaking fees, or expert testimony fees from entities that have a financial interest in the results and materials of this study. Please enumerate.

☑ None

☐ I have a consulting relationship, as described below:

Patents: A planned, pending, or awarded patent on this work by any of the authors or their institutions. Please explain.

☑ None

☐ I or my institution has a patent related to this work, as described below

Declaration: I declare that I have read *Biomaterials Research's* full Conflict of Interest Policy and have disclosed all declarable relationships as defined therein, if any.

This form was submitted on 09/19/2024

Name Tongmeng Jiang

Signature Tongmeng Jiang 数字签名者：Tongmeng Jiang 日期：2024.09.19 07:53:14 +08'00'
