# Supplementary material for: From Bench to Bedside: The Role of Extracellular Vesicles in Cartilage Injury Treatment
Source: Biomater Res. 2024 Nov 22;28:0110. doi: 10.34133/bmr.0110 (PMC11582190; doi:10.34133/bmr.0110)
Supplement: Supplementary 1 — Figs. S1 to S4 Tables S1 and S2 [file bmr.0110.f1.zip › Supplementary figures.doc]

**Front Matter**

Full title：

From Bench to Bedside: The Role of Extracellular Vesicles in Cartilage Injury Treatment

Short title:

Extracellular Vesicles for Cartilage

**Authors**

Pan Jin^1†^, Huan Liu^1†^, Xichi Chen^1^, Wei Liu^1^, Tongmeng Jiang^2,3*^

**Affiliations**

1 Health Science Center, Yangtze University, Jingzhou 434023, Hubei, China

2 Key Laboratory of Emergency and Trauma of Ministry of Education, Key Laboratory of Haikou Trauma, Key Laboratory of Hainan Trauma and Disaster Rescue, The First Affiliated Hospital, Hainan Medical University, Haikou 571199, China

3 Engineering Research Center for Hainan Bio-Smart Materials and Bio-Medical Devices, Key Laboratory of Hainan Functional Materials and Molecular Imaging, College of Emergency and Trauma; Hainan Provincial Stem Cell Research Institute; Hainan Academy of Medical Sciences, Hainan Medical University, Haikou 571199, China

† These authors contributed equally to this work

^*^Address correspondence to: jiangtongmeng@muhn.edu.cn

Supplementary Figures

**Fig. S1**. Heterogeneity and separation methods of extracellular particles (EPs). (A) Classification of EPs. EV: extracellular vesicle; SV: synthetic vesicle; ACDV: artificial cell-derived vesicle; NVEP: non-vesicular extracellular particle. (B) Different separation methods affect the recovery (yield) and specificity of EPs. Reproduced from ref. [22] with permission. Copyright 2024 The Authors. Journal of Extracellular Vesicles published by Wiley Periodicals, LLC on behalf of the International Society for Extracellular Vesicles.

**Fig. S2**. Infrapatellar fat pad (IPFP) MSCs-derived exosomes (MSC^IPFP^-Exos) inhibit mTOR signaling pathway therefore promote autophagy in IL-1β treated chondrocytes. Reproduced from ref. [72] with permission. Copyright 2019 Elsevier Ltd. All rights reserved.

**Fig. S3**. Effect of macrophage-derived EVs on cartilage. (A) M2 macrophages-derived apoptotic bodies (M2-ABs) alleviate the progression of osteoarthritis (OA) via triggering the switch of M1 to M2 transformation. Reproduced from ref. [21] with permission. Copyright 2022 The Authors. Publishing services by Elsevier B.V. on behalf of KeAi Communications Co., Ltd. (B) Macrophage-derived EVs initiate noncanonical pyroptosis in chondrocytes leading to cartilage catabolism in OA. Reproduced from ref. [94] with permission. Copyright 2023 American College of Rheumatology.

**Fig. S4**. Extraction and application of plant exosome-like nanovesicles (PELNVs). (A) Extraction of PELNVs. Reproduced from ref. [130] with permission. Copyright 2020 The American Society of Gene and Cell Therapy. (B) Multiple applications of PELNVs. Reproduced from ref. [122] with permission. Copyright 2023, The Author(s). Distributed under CC BY 4.0, published by Springer Nature.
