# Supplementary material for: From Bench to Bedside: The Role of Extracellular Vesicles in Cartilage Injury Treatment
Source: Biomater Res. 2024 Nov 22;28:0110. doi: 10.34133/bmr.0110 (PMC11582190; doi:10.34133/bmr.0110)
Supplement: Supplementary 1 — Figs. S1 to S4 Tables S1 and S2 [file bmr.0110.f1.zip › Supplementary tables.doc]

**Supplementary tables**

Full title：

From Bench to Bedside: The Role of Extracellular Vesicles in Cartilage Injury Treatment

Short title:

Extracellular Vesicles for Cartilage

**Authors**

Pan Jin^1†^, Huan Liu^1†^, Xichi Chen^1^, Wei Liu^1^, Tongmeng Jiang^2,3*^

**Affiliations**

1 Health Science Center, Yangtze University, Jingzhou 434023, Hubei, China

2 Key Laboratory of Emergency and Trauma of Ministry of Education, Key Laboratory of Haikou Trauma, Key Laboratory of Hainan Trauma and Disaster Rescue, The First Affiliated Hospital, Hainan Medical University, Haikou 571199, China

3 Engineering Research Center for Hainan Bio-Smart Materials and Bio-Medical Devices, Key Laboratory of Hainan Functional Materials and Molecular Imaging, College of Emergency and Trauma; Hainan Provincial Stem Cell Research Institute; Hainan Academy of Medical Sciences, Hainan Medical University, Haikou 571199, China

† These authors contributed equally to this work

^*^Address correspondence to: jiangtongmeng@muhn.edu.cn

**Table S1** shows the sources, characteristics and effects of extracellular vesicles (EVs) used in cartilage repair reported in the literature.

| Sources | Spices | Extracts of EVs | Engineering modification methods | Active components of EVs | Identification method | Dosage | Delivery method | Route of administration | Type of animal model | Safety and efficacy | Mechanical loading change | Reference |
| --- | --- | --- | --- | --- | --- | --- | --- | --- | --- | --- | --- | --- |
| Mammals | Human | Umbilical cord mesenchymal stem cells (UCMSCs) | None | Exosomes | Flow cytometry (CD90^+^, CD105^+^; CD34^-^, CD45^-^), TEM (an average diameter of about 100 nm), NTA (50 - 200 nm), WB (CD9+, CD63+, Alix+, TSG101+; Calnexin-) | 10 μL PBS or hucMSCs-EVs (10^11^ particles /mL) | Intra-articular injection | hucMSCs-EVs (10^11^ particles /mL) were injected intra-articularly twice a week to the mice | Mice knee OA model (ACLT + pMMx) | Anti-inflammation; Protecting cartilage by interacting with METTL3 to reduce m6A levels of NLRP3 in macrophages, thereby up-regulating COL2A1 and Aggrecan, and down-regulating ADAMTS5 and MMP13 | No relevant tests were performed | [38] |
| Mammals | Human | UCMSCs | None | Exosomes | TEM (30–200 nm), DLS (30–200 nm), Flow cytometry assay (CD63: 78.1%, CD81: 79%), WB (CD63^+^, CD81^+^) | 100 μL hUC-MSCs-Exos in PBS (10^11^ particles/mL of exosomes) | Intra-articular injection | 10^11^ particles/mL of exosomes on day 1 and 4 of every week from the 5th to 8th week post-surgery | Rat knee OA model (ACLT + pMMx) | Regulating the polarization of macrophages; reverse the damage of IL-1β to OA-like chondrocytes *in vitro* | No relevant tests were performed | [39] |
| Mammals | Human | UCMSCs | None | EVs (not clearly marked in the paper) | TEM (a spherical shape with a hypodense center), NTA (30-150nm), Flow cytometry (CD63: 52.5%. CD81: 84.9%), WB (CD63^+^, CD81^+^, TSG101^+^; CALNXIN^-^) | Not explicitly state | Intra-articular injection | Intra-articular injection with hUCMSCs-EVs once every week | Rat knee OA model (ACLT) | Promoted the polarization of M2-type macrophages and the expression of anti-inflammatory factors, and inhibit the level of inflammation-related factors in OA chondrocytes caused by IL-1β, thus exerting the immunomodulatory potential and alleviating the cartilage degeneration of OA | No relevant tests were performed | [40] |
| Mammals | Human | UCMSCs | The exosomes (EXOs) were engineered on membranes with a designed chondrocyte-targeting polymers, and encapsulated within thiolated hyaluronic acid microgels to form a “two-phase” releasing system | Exosomes | NTA (mostly 143.1 nm), WB (CD63 ^+^, TSG101^+^; TOMM20^-^), TEM (typical exosome morphology) | Not explicitly state | Intra-articular injection/microgel | Eighth week after the OA model was established. UCMSC-EXOs; chondrocyte-targeting UCMSC-EXOs; UCMSC-EXOs in HA-SH microgels; chondrocyte-targeting UCMSC-EXOs in HA-SH microgels. | Rat knee OA model (ACLT) | Inhibited p53 signaling pathway, decreased the expression of OA cartilage aging gene, improved the vitality of OA cartilage cells, and promoted the secretion of hyaline cartilage matrix and COL II, allowing its cartilage to regenerate | No relevant tests were performed | [41] |
| Mammals | Human | UCMSCs | None | Exosomes | TEM (30-200 nm with a typical membrane structure), ZETASIZER Nano series-Nano-ZS (82.5% of the hUC-MSCs-Exos ranging 30-200 nm), WB (CD9^+^, CD63^+^, CD81^+^, TSG101^+),^ Flow cytometry (CD63:71.3%, CD81:79.2%) | 4×10^7 particles/mL | None | HUC-MSCs-Exos co-culture with macrophages and chondrocytes in transwell systems). | *In vitro* only. | Reduced the apoptosis of chondrocytes and promoted the proliferation and differentiation of chondrocytes | No relevant tests were performed | [42] |
| Mammals | Human | UCMSCs | Exogenous miR-223 was loaded into hUC-EVs by electroporation, and a collagen II-targeting peptide (WYRGRL) was modified onto the surface of hUC-EVs | miR-223 | Flow cytometry (CD73^+^, CD90^+^; CD34^-^, CD45^-^)，TEM (a typical spherical bilayer membrane structure), NTA(average diameter of 142.9nm, and the main peak at 131.3nm), WB (CD81^+^, Tsg101+, Alix^+^; Calnexin^-^) | 50 μL EV (10^10^ particles/ml) | Intra-articular injection/Engineering modified technology | Intra-articular injections of 50 μL PBS or 50LEV (10^10^ particles/ml) for 4 weeks (once a week) 1 week after the MIA injection were administered to rats. | Rat knee OA model (MIA-induced OA) | Inhibited the activation of the NLRP3 pathway, thus reducing inflammatory response and apoptosis of chondrocytes, promoting cartilage repair and recovery of joint function | No relevant tests were performed | [43] |
| Mammals | Human | UCMSCs | The sEVs (10 × 10^8^ particles/mL) were added into the mixture and composited hydrogels were obtained after chemical cross-linking by ultraviolet radiation for 3 min. | sEVs | TEM (a spherical microvesicle structure, 50-150nm), NTA (average diameters about 142.9nm, 99.2% ranging at 30-150nm), WB (CD81^+^, CD63^+^, CD9^+^) | 10×10^8^ particles/mL | Surgical implantation to fill the defect/composited hydrogel | Gelma/nanoclay/sEVs (Gel-nano-sEVs) (10 × 10^8^ particles/mL) | A full-thickness cylindrical cartilage defects (2 mm in diameter and 1.5 mm in depth) | Promoted cartilage regeneration by activating the PTEN/AKT signaling pathway and sustained release of EVs (up to 31 days) | The Gelma/nanoclay hydrogel showed the highest ultimate strength increased significantly from 48.2±8.1 kPa to 259.8±35.6 kPa with addition of nanoclay | [29] |
| Mammals | Human | UCMSCs | 3D printed with upper scaffold adds black phosphorus (BP) and hUMSCs exosomes (exos) in GelMA; the lower scaffold, in addition to BP and hUMSCs exos, *β*-TCP). | Exosomes | TEM (a spherical microvesicle structure), NTA (average diameters about 115.7nm), WB (the exosomal surface marker CD9^+^, CD63^+^, TSG101^+^) | Not explicitly state | Implanted with 3D bioprinted scaffolds | GelMA/BExo//GelMA/β-TCP/BExo maintaining 6 weeks and 14 weeks post-surgery | Rabbit cartilage defect with a diameter of 4 mm and a depth of 4 mm was made on the center of the femoral trochlea with an electric drill. | The hydrogel scaffold could promote the differentiation and repair of cartilage tissue, exos could be released continuously within 28 days, and the final release rate reached 92%. | After adding 3% β-TCP, the maximum compression stress of the GelMA hydrogel scaffold increased significantly, reach to 48.99 ± 15.67 kPa. The Young's modulus of GelMA/β-TCP increased from 232.86 ± 12.45 to 306.17 ± 17.82 kPa by adding 1% and 3% β-TCP. | [30] |
| Mammals | Human | Embryonic mesenchymal stem cells (EMSCs) | None | Exosomes | NTA (about 100 nm), WB (CD81^+^, ALIX^+^; TSG101^+^) | 100 μg exosomes per 100 μl of injection | Intra-articular injection | 100 μg exosomes per 100 μl of injection administered immediately after the surgery and subsequently on a weekly basis. | Rat osteochondral defect model  (1.5 mm diameter and 1 mm depth) | Promoted the proliferation (PCNA), reduced the apoptosis of (cleaved caspase-3, CCP3), and inhibited the production of inflammatory cytokines | No relevant tests were performed | [44, 45] |
| Mammals | Human | EMSCs | None | Exosomes | TEM (38nm - 169nm and the mean value was 78nm.), WB (CD63^+^ and CD9^+^) | 5 μL ESC-MSCs (1 × 10^6^/joint) | Intra-articular injection | Multiple injections of 5 μL ESC-MSCs (1 × 10^6^/joint) every 3 days for 4 weeks | Mice knee OA model (DMM surgery) | Increased collagen II synthesis and decreased ADAMTS5 expression, thereby reducing cartilage destruction and matrix degradation in a destabilization of the DMM mouse model of OA | No relevant tests were performed | [46] |
| Mammals | Human | EMSCs | None | Exosomes | NTA (100-200 nm), WB (CD81+, ALIX^+^ and  TSG101^+^) | 100 μg of exosomes in 50 μl | Intra-articular injection | Weekly intra-articular injections of either 100 μg of exosomes in 50 μl of PBS for 2, 4 or 8 weeks | Rat temporomandibular joint osteoarthritis model (induced by MIA injection) | Inhibited inflammation, restored stromal homeostasis, promoted cell proliferation and reduced cell apoptosis, so as to repair the damage of the temporomandibular joint | No relevant tests were performed | [47] |
| Mammals | Human | EMSCs | None | Exosomes | WB (CD9+, CD63^+^, and CD81^+^), NTA (average size 177±49nm), | 20 µg /injection | Intra-articular injection | pExo by single (OA+pExo1, day 5) or triple (OA+pExo3, days 5, 12, and 19) | Rat knee OA model (MCLT+MMT) | Promoted chondrocyte migration and proliferation by regulating cell growth-related signaling pathways (such as the AKT and ERK signaling pathways) and inhibited inflammation and the process of cartilage degradation in vitro | No relevant tests were performed | [48] |
| Mammals | Human | EMSCs | None | Exosomes | NanoOrange Protein Quantification Kit (100 to 200 nm and CD81^+^, ALIX^+^, and TSG101^+^) | Each injection comprised 200 μg of exosomes suspended in 1 mL of 3% (w/v) HA. | Intra-articular injection/Hydrogel | For exosome + HA treatment, each injection comprised 200 μg of exosomes suspended in 1 mL of 3% (w/v) HA on days 7 and 14 post-surgery. | Rabbits osteochondral defect model (Bilateral critical-size osteochondral defects (4.5-mm diameter and 1.5-mm depth) | The combination of MSC exosomes and HA exhibited a superior cartilage reparative response when compared with HA alone | The Young's moduli of exosome + HA repaired tissues at the inferior (12.68 MPa) and superior points (18.03 MPa) | [49] |
| Mammals | Human | EMSCs | None | Exosomes | TEM | 1 mg exosomes in 1 mL PBS | Intra-articular injection | The Exosome+HA group received a sequential administration of 1 mg exosomes in 1 mL phosphate-buffered saline (PBS), followed by 1 mL HA | Pig osteochondral defect model (Bilateral osteochondral defects (6-mm diameter and 1-mm depth)) | Promoting functional cartilage and subchondral bone repair | No relevant tests were performed | [50] |
| Mammals | Human | EMSCs | None | Extracellular Vesicles (Not explicitly stated) | NanoOrange Protein Quantification Kit (50 - 200 nm; ALIX^+^, TSG101^+^, and CD81^+^) | 1 mg MSC-EVs in 1 mL PBS | Intra-articular injection | 1 mg MSC-EVs in 1 mL PBS | Pig osteochondral defect model | Intraarticular injections of MSC-EVs were effective in enhancing healing of subchondral bone instead of cartilage and MSC-EVs impaired optimal cartilage repair | No relevant tests were performed | [51] |
| Mammals | Human | Synovial mesenchymal stem cells (SMSCs) | None | Extracellular Vesicles (Not explicitly stated) | TEM (both EVs and LPS-pre EVs were round or disk-shaped), NanoSight (Most EVs and LPS-pre EVs ranging 50 - 200nm), WB (CD9^+^, CD63^+^, Alix^+^, and TSG101^+^ in EVs and LPS-pre EVs but not in SMSC) | 10 μl, 10^11^ particles/ml | Intra-articular injection | PBS-EVs (10 μl, 10^11^ particles/ml), or PBS-LPS-pre EVs (10 μl, 10^11^ particles/ml) twice-weekly for 6 weeks | Mice knee OA model (DMM) | Inhibited extracellular matrix (ECM) degradation and alleviated knee OA | No relevant tests were performed | [53] |
| Mammals | Human | SMSCs | None | Exosomes | DLS (30 - 150 nm), TEM (hollow spherical microvesicles), WB (CD63^+^, CD9^+^, CD81^+^ and Alix^+^) | 100 μL; 10^11^ exosome particles/mL | Intra-articular injection | OA+SMSC-Exos group (rats received articular cavity injection of SMSC-Exos (100 μL; 10^11^ exosomes particles/mL)); OA+SMSC-140-Exos group (Rats received articular cavity injection of SMSC-140-Exos (100 μL; 10^11^ exosomes particles/mL)). | Rat knee OA model (ACLT+DMM) | Modification of SMSC-Exos through overexpression of miR-140-5p compensated for a deficiency of cartilage ECM and promoted the regeneration of cartilage tissue | No relevant tests were performed | [54] |
| Mammals | Human | SMSCs | None | Exosomes | NTA (100 - 120 nm), TEM (a typical cup shape), WB (CD63^+^ and CD81^+^; TFIIB^-^ | 30 μL; 10^11^ Exo particles/mL | Intra-articular injection | SMSC-Exos [30 μL; 10^11^ Exo particles/mL] and saline); SMSC-155-5p-Exos [30 μL; 10^11^ Exo particles/mL] and saline | Mice knee OA model | Promoted the proliferation and migration of chondrocytes, and the secretion of ECM-related proteins CoII and SOX9 | No relevant tests were performed | [55] |
| Mammals | Human | SMSCs | None | Exosomes | TEM (50 nm–100 nm), WB (CD9^+^, CD63^+^ and HSP70^+^) | None | None | Cells were divided into three groups: miR-129-5p mimic, miR-  129-5p inhibitor and the negative control | *In vitro* only | Alleviated IL-1β-induced inflammatory response and apoptosis of chondrocytes by targeting HMGB1 | No relevant tests were performed | [56] |
| Mammals | Human | SMSCs | None | Exosomes | TEM (the single membrane structure of SMSC-Exos), DLS (30 - 150nm), WB (CD63^+^, CD9^+^, and Alix^+^) | 2 µg exosomes | None | Cells were divided into three groups: Control, SMSC-Exos and SMSC-212-5p-Exos | *In vitro* only | Inhibited the upregulation of IL-1β-induced E74 Like ETS Transcription Factor 3 (ELF3), reduced the expression of degenerate molecules and matrix metalloproteinases, and reduced the expression of inflammatory molecules. | No relevant tests were performed | [57] |
| Mammals | Human | BMMSCs or BMSCs | None | Extracellular Vesicles (Not explicitly state) | Electron microscopy and NTA (40 - 150nm), WB (CD63^+^ and CD9^+^) | Not explicitly state | None | None | *In vitro* only | Promoted the production of proteoglycan and type II collagen, and restored and maintained the homeostasis of cartilage | No relevant tests were performed | [60] |
| Mammals | Human | BMSCs | None | Exosomes | NTA (50-150nm), TEM (a cup-shaped or round morphology, 50-150nm), WB (CD9^+^, CD63^+^, CD81^+^, and HSP70^+^). | 15 µl Exos (500g/mL) | Intra-articular injection | MSC-Exos and MSC-miR-92a-3p-Exos groups were injected with 15 µl MSC-Exos (500g/mL) or 15 µl MSC-miR-92a-3p-Exos in PBS (500g/mL). | Mice knee OA model (Collagenase-induced) | Cartilage degradation was reduced by inhibiting the WNT5A signaling pathway | No relevant tests were performed | [61] |
| Mammals | Human | BMSCs | None | Exosomes | TEM (the typical 30-150 nm vesicle structure of the exosomes), WB (CD9^+^, CD63^+^, and TSG101^+^), | Not explicitly state | Intra-articular injection | Injection of BMSC-EXOs, and pBMSC-EXOs into the OA, BMSC-EXOs, and pBMSC-EXOs, respectively, on days 7, 14, and 21. | Mice knee OA model (collagenase VII-induced) | Improved the knee cartilage injury of the OA mouse model induced by collagenase, and reduced the OARSI score significantly | No relevant tests were performed | [62] |
| Mammals | Human | BMSCs | None | Extracellular Vesicles (Not explicitly state) | NTA (the average size of 124.6 nm ± 81.1 nm), SEM (90-120 nm), WB (CD9^+^, CD63^+^, and CD81^+^)). | 10 µg/ml | None | Stimulated with IL-1 for 24 h, and incubated with hBMSC-EVs (10 µg/ml) for 24 h | *In vitro* only | Reversing the down-regulation of chondrocyte degradation gene expression under IL-1β stimulation and reducing the phosphorylation level of inflammation-related signaling pathways | No relevant tests were performed | [63] |
| Mammals | Human | BMSCs | None | Exosomes | TEM (exosomes exhibited a cup-shaped or nearly round morphology with a diameter of approximately 100 nm), NTA (major peak of particle size was approximately 116 nm), WB (CD9^+^, CD63^+^, and HSP70^+^) | 20 g/mL best | None | The inflammatory chondrocytes were treated with different concentrations of exosomes (0, 1, 5, 10, 20, and 50 g/mL) | *In vitro* only | Significantly increased the survival of IL-1β-induced chondrocytes and inhibited their apoptosis | No relevant tests were performed | [64] |
| Mammals | Human | BMSCs | None | Exosomes | TEM (the majority of these vesicles exhibited typical sphere-shaped bilayer membrane structure), NTA (average diameter of 143.9 nm and the main peak at 113 nm), WB (TSG101^+^, CD81^+^ and CD63^+^; negative for the non-exosomal marker) | 100 μl （400 μg/ml） | Intra-articular injection | Injection with 100 μl of BMSC-derived exosomes (400 μg/ml) twice a week for 4 weeks. | Rat knee OA model (ACLT+MMx) | Enhanced chondrocyte proliferation and promoted cartilage matrix synthesis, while also inhibiting inflammation associated with osteoarthritis | No relevant tests were performed | [65] |
| Mammals | Human | BMSCs | None | Exosomes | TEM (a cub-like shape coated with bilayer membranes), NTA (average diameter 131.2nm), WB (CD81+, TSG101+, and Flotillin 1+; Actin-) | 300 μl of 1×10^10^ particles/ml (low dosage) or 5×10^10^ particles/ml (high dosage) | Intra-articular injection | One week post-surgery, rabbits in the EXO group received 300 μl of 1×10^10^ particles/ml (low dosage) or 5×10^10^ particles/ml (high dosage) by intra-articular injection | Rabbit osteochondral defect model (4mm diameter, 3mm depth) | Promoted the repair of bone cartilage defects of the patellar groove in rabbits | No relevant tests were performed | [66] |
| Mammals | Human | BMSCs | None | Exosomes | TEM (rounded nanoparticles of ∼100 nm), NTA (the size of N-Exos and H-Exos was predominantly around 117.9 nm and 122 nm), WB (TSG101^+^, CD9^+^, and CD63^+^; calnexin^-^) | 10 μL SF hydrogel; The concentrations of ACs and exosomes in each group were 5 × 10^6^ cells/mL and 1 × 10^9^ particles/mL, respectively | Surgical implantation to fill the defect/Hydrogel | SF/N-Exos group, defects were injected with 10 μL SF hydrogel blended with ACs and N-Exos; SF/H-Exos group, defects were injected with 10 μL SF hydrogel blended with ACs and H-Exos; and SF/miR-205–5p-N-Exos group, defects were injected with 10 μL SF hydrogel blended with ACs and miR-205–5p-N-Exos. | Rat osteochondral defect model (2 mm in diameter and 1.5 mm in depth) | Sustainably released exosomes for 31 days and effectively promoted cartilage regeneration in a rat model of cartilage deficiency | No relevant tests were performed | [67] |
| Mammals | Human | Adipose mesenchymal stem cells (ADSCs) | Engineering exosomes derived from MSCs^SC^ that are fusing an chondrocyte-binding peptide CAP with the exosomal membrane protein Lamp 2b, and miR-199a-3p loading by electroporation | Exosomes | NTA (varied from 70 to 150nm and the main peak diameter was 118.9nm), TEM (a sphere-shaped morphology and a size around 100nm), WB (CD9^+^, CD81^+^, Alix^+^ and Tsg101^+^) | 50 μl Exos^SC^ (2×10^10^particles/ml) | Intra-articular injection | 50μl ExosSC/week (2×10^10^ particles/ml) for 6 weeks in rats; 10 μl ExosSC/week (1 × 10^10^ particles/ml) for 4 weeks in mice. | Rat knee OA model (DMM+ACLT), Mice knee OA model (DMM) | Inhibited the expression of mTOR to induce chondrocyte autophagy and promote cartilage repair | No relevant tests were performed | [34] |
| Mammals | Human | ADSCs | None | Exosomes | TEM (most of these vesicles exhibited typical sphere-shaped bilayer membrane structure, whose diameter were about 100 nm), NTA (the average diameter of 121.9 nm and the main peak located at 125.9 nm), WB (CD81^+^, CD9^+^ and CD63^+^) | 10 μl MSC^IPFP^-Exos (10^10^ particles/ml) | Intra-articular injection | 10 μl MSC^IPFP^-Exos (10^10^ particles/ml) for 4 weeks or 6 weeks (twice a week) | Mice knee OA model (DMM) | Up-regulate autophagy by inhibiting the mTOR pathway, reducing the damage of IL-1β to cartilage, enhancing anabolism and inhibiting catabolism | No relevant tests were performed | [72] |
| Mammals | Human | ADSCs | None | Microvesicles (MV) and exosomes (EX) | Tunable resistive pulse sensing (TRPS)(mean diameter values of 295nm and 115nm for MV and EX), TEM | MV (3.6×10^7^ particles/ml), EX (7.2×10^7^ particles/ml) | None | Chondrocytes stimulated with interleukin (IL)-1β (10 ng/ml) in the presence MV (3.6×10^7^ particles/ml), EX (7.2×10^7^ particles/ml) or CM (0.4 ml for 24-well plates, 1 ml for 6-well plates or 1.5 ml for 3.5 cm plates). | *In vitro* only | Reduce the activity of matrix metalloproteinases and the expression of MMP-13 while enhancing the expression of collagen II to protect cartilage | No relevant tests were performed | [71] |
| Mammals | Human | ADSCs | None | Small extracellular vesicles | DLS, NTA, TEM (double-layer membrane structure, mean diameter was determined to be 86.46 nm), Flow cytometry and WB (CD9^+^, CD63^+^, CD81^+^ and Alix^+^; GM130^-^, Calnexin^-^) | EVs (1×10^8^ particles), 30 μL per joint for rats; EVs (1×10^8^ particles), 6 µL per joint for mice | Intra-articular injection | Once a week for 21 days for rats; once a week for 11 weeks for mice | Rat knee OA model (MIA-induced) and Mice knee OA model (DMM) | Reduced the activity of matrix metalloproteinases and the expression of MMP-13, while enhancing the expression of collagen II to protect cartilage | No relevant tests were performed | [74] |
| Mammals | Human | ADSCs | None | Small Extracellular Vesicles | NTA (size distribution of 152.4+/10.4nm), TEM (round or cup-shaped vesicles with a prevalent size of 50-100nm), Western blot | 10 µg/mL | None | Co-cultures were assembled, the medium containing IL-1 was substituted with fresh DMEM+10% FBS containing 10 µg/mL of sEV | *In vitro* only | Inhibited the activation of the NF-κB signaling pathway, down-regulates ADAMTS5, and counteracts the IL-1β-induced inflammatory effects | No relevant tests were performed | [75] |
| Mammals | Human | ADSCs | None | Exosomes | TEM, WB (CD63^+^, TSG101^+^ and CD9^+^) | 100 µg/250 µl | Intra-articular injection | After 2 weeks of MIA injection, Exos (100 µg/250 µl) were injected into the joint | Rat knee OA model（MIA-induced) | Regulated the WNT-β-catenin signaling pathway by inhibiting the expression of WNT3 and WNT9a, alleviating chondrocyte degradation and synovial fibrosis | No relevant tests were performed | [76] |
| Mammals | Human | ADSCs | None | Exosomes | TEM (a bilayer membrane structure), NTA (an average diameter of 140.3nm), WB (ALIX^+^, TSG101^+^, and CD81^+^; Calnexin^-^) | 500 µg | Implanted with 3D bio-printed scaffold | Printed Bi-Hydrogel-Exos scaffold | Rat osteochondral defect model (2.5 mm in diameter and 3 mm in depth) | Up-regulated the expression levels of aggregation protein (ACAN), gel prototype (COL II) and SRY-box transcription factor 9 (SOX9), and accelerated the synchronous regeneration of cartilage and subchondral bone tissue in OA rats | No relevant tests were performed | [77] |
| Mammals | Human | iPSCs | None | Exosomes | NTA (50–150 nm), TEM(the size of 30–60 nm that exhibited spherical morphology), WB (CD9^+^, CD63^+^ and CD81^+^) | 20 μL containing 1 × 10^11^ mL^−1^ exosomes | Surgical implantation to fill the defect  /Hydrogel | 20 μL hydrogels or suspension containing 1 × 10^11^ mL^−1^ exosomes | Rabbitsosteochondral Defect Model (4 mm in diameter and 3 mm in depth) | Promoted the repair and regeneration of rabbit patellar bone cartilage defect | No relevant tests were performed | [79] |
| Mammals | Human | iPSCs | None | small extracellular vesicles | Nanoflow cytometry (verage diameter of 76.92 nm) and zeta potential analysis (-15.6 mV), TEM (a typical cup-shaped morphology), WB (positive expression of EV-associated surface makers such as CD9^+^, CD63^+^, TSG101^+^; GM130^-^, lamin A/C^-^) | 100 µL 1 × 10^10^ p/mL | Intra-articular injection/Hydrogel | For the multi-sEVs group, 100 µL 1 × 10^10^ p/mL suspension of iMSC-sEVs was given on the 7th, 14th, 21st, and 28th day after surgery | Rat knee OA model (ACLT+pMMx) | Inhibition of the NF-κB signaling pathway promoted the polarization of macrophages from M1 to M2, thereby enhancing cartilage regeneration | No relevant tests were performed | [80] |
| Mammals | Human | Chondrocytes | The surface of exosomes  were binding to chondrocyte-affinity peptide (CAP)  by CAP-Lamp2b or CAP-EGFP-lamp2b plasmids transfection and loaded with miRNA-140 by electroporation | Exosomes | WB (CD63^+^ and CD9^+^), TEM (typical cup-shaped structures of 40-200 nm), NTA (40-200 nm) | 100 μg | Intra-articular injection | Rats underwent DMM  surgery and exosome preparations (exosome/miR-140 or CAP-exosome/miR-140, 100 μg of  exosomes in 100 μl PBS) were administrated to OA rats via intra-articular injection once every week for  consecutive four weeks | Rat knee OA model (DMM) | Targeted delivery of miR-140 by CAP-exosomes to chondrocytes increases miR-140  level and suppresses MMP-13 and Adamt-5, and reverse the activated ECM degrading enzymes by  IL-1β-induction in chondrocytes in vitro. The CAP-exosome/miR-140 treatment  group have smooth and flat cartilage surface almost identical to that of the healthy control in vivo. | No relevant tests were performed | [81] |
| Mammals | Human | Platelet-rich plasma (PRP) | Embedded in gels constructed by 17.9% (w/w) Poloxamer 407 and 5% (w/w) 188 | Exosomes | NTA, TEM (round nanoparticles; 40 - 100m), WB (CD9^+^, CD63^+^, and CD81^+^) | 4µg Exo/2µl Gel | Joint injection/Hydrogels | After surgery, 2µl PBS, 2µl blank Gel, 2µl PBS containing 2µg Exo, or 2µl Exo-Gel (4µg Exo/2µl Gel) were respectively injected into subtalar joint | Mice subtalar osteoarthritis (transecting lateral ligaments (ATFL/CFL) | Alleviated cartilage damage in a mouse model of subtalar osteoarthritis, and Exo-Gel sustained exosomes release in vivo for up to 28 days | No relevant tests were performed | [83] |
| Mammals | Mice | BMSCs | None | Exosomes, microparticles | TEM (round shaped vesicles surrounded by a bilayer membrane with heterogeneous sizes of MPs), NTA, Flow cytometry analysis (CD9^+^ and CD81^+^ in Exos; Sca-1^+^, CD44^+^, CD29^+^ in MPs), Western blot analysis (Hsp70^+^, Tsg101^+^ and Alix^+^ in Exos) | 250 ng of Exos and 600 ng of MPs. | Intra-articular injection | EVs were injected intravenously at day 18 and 24. | Mice knee OA model (Collagen-induced  ) | In inflammatory arthritis, mesenchymal stem cell (MSCs) -derived exosomes (Exos) and microparticles (MPs) both have anti-inflammatory effects and can alleviate bone degradation, but Exos is more effective in inhibiting inflammation. | No relevant tests were performed | [84] |
| Mammals | Mice | BMSCs | None | Exosomes | NTA (the exosome diameter mainly was 87nm, ranging 95.4±18.9nm), TEM (round or oval vesicles with double membraned structure, 30-100nm), WB (CD63^+^ and Alix^+^) | 100 µL exosomes (10^11^ exosome particles/mL) | Intra-articular injection | The articular cavity was injected with 100 µL exosomes twice/w (10^11^ exosome particles/mL were injected into knee joints | Mice knee OA model (ACLT or Collagen-induced  ) | Decreased the apoptosis index (AI) of OA mice by negatively regulating Elf3, promoted the proliferation and differentiation of osteoblasts, and alleviated OA in mice | No relevant tests were performed | [85] |
| Mammals | Mice | BMSCs | None | Exosomes | TEM (round or oval vesicles), NTA (60–150 nm), WB (TSG101^+^, CD63^+^, and CD81^+^) | 0.5 mg/ml | Intra-articular injection | Rats in the BMSCs-exo group were injected with BMSCs-exo 0.5 mg/mL in the tailward intra-articular | Rat knee OA model (MCLT+ACLT+PCLT+MMT) | Decreased the percentage of M1-type macrophages and increased the percentage of M2-type macrophages, thereby improving the pathological status of osteoarthritis | No relevant tests were performed | [86] |
| Mammals | Mice | BMSCs | None | Nanovesicle | NTA (average size of MSC-NVs was 130.7 nm; the percentage of vesicles with a size of 30–150 nm was 75.52%), TEM (100–150 nm, spherical morphology, and an intact membrane structure), WB CD9^+^ and CD63^+^) | 10 µL (80 µg mL^-1^) | Intra-articular injection/Hydrogels | Four weeks after DMM MSC-NVs (80 µg mL-1), or GelMA-NVs were administered 10 µL intra-articular injections respectively | Mice knee OA model (DMM) | Significantly polarized macrophages towards M2 phenotype and inhibited the secretion of pro-inflammatory cytokines, thus reducing the severity of cartilage injury. The sustained release time of nanovesicles (MSC-NVs) derived from mesenchymal stem cells was up to 30 days | The compressive strength of GelMA hydrogels is 12.096 kPa, whereas the compressive strength of GelMA-NVs increases to 16.779 kPa, | [87] |
| Mammals | Mice | BMSCs | Embedded in different hydrogels constructed by chitosan, catechol-modified chitosan (CAT-C), β-glycerophosphate disodium salt hydrate and Dialdehyde-functionalized-PEG | Exosomes | NTA (the particle size distribution of EXO was normally, 30 – 150 nm, with an average size of 127.4 nm), TEM (obvious spherical morphology with a significant lipid bilayer structure), WB (HSP70^+^ and CD81^+^) | 150 µl | Intra-articular injection/Hydrogels | 150 µl of the preparations were injected in situ into the knee joint cavity, respectively, and then the rats were fed normally for 5 weeks | Rat knee OA model (100 µl of 4% papain solution was injected into the knee joint cavity) | Decelerated cartilage degeneration, promoted cartilage regeneration, inhibited MMP13 secretion, and showed cartilage protection *in vivo* | No relevant tests were performed | [88] |
| Mammals | Mice | BMSCs | HMPs hydrogel and HMPs@Exos were prepared by microfluidic technology | Exosomes | NTA (30 - 150nm), TEM (a cup-shaped or round morphology with a diameter of 50-150nm), WB (CD9^+^, CD63^+^, and TSG101^+^) | 10 μL Exos (5 μg) | Intra-articular injection directly or with hydrogels | Intra-articular injections of 10 μL Exos (5μg) twice a week for 4 weeks; 30 μl hydrogels containing 20 μg Exos at the first day for 4 or 8 weeks | Mice knee OA model (DMM) | Extended exosome retention time, inhibited ADAMTS4 expression and enhanced extracellular matrix integrity, which is beneficial for the long-term treatment of osteoarthritis | No relevant tests were performed | [89] |
| Mammals | Mice | Macrophage | None | Apoptotic bodies (ABs) | SEM, DLS (1 μm), WB (CD86^+^ and CD206^+^; INOS^-^ and ARG-1^-^), Confocal  microscopy | M1-ABs (10 μg/10 μL) and  M2-ABs (10 μg/10 μL) | Intra-articular injection | Injected  into the joint cavity for 5 consecutive  weeks, once a week. | Mice knee OA model (ACLT) | Alleviated the inflammatory response caused by M1 macrophages, prevented articular cartilage injury, and improved gait abnormalities in OA mice. | No relevant tests were performed | [21] |
| Mammals | Mice | SMSCs | None | Exosomes | NTA, TEM (the classic morphology as a vacuolar cup surrounded by a double membrane, 50-160nm), WB (CD63^+^, CD81^+^ and CD9^+^) | 100 μl (1×10^11^ exosome particles/mL) | Intra-articular injection | Three weeks later, BMP-7 exo (1×10^11^ exosome particles/mL) was intraarticular injected for the rats in BMP-7-exo group, 100 μl every time, once every three days, and same volume and concentration of exo was used in exo group | Rat knee OA model (unilateral intraarticular injection with 8% sodium iodoacetate) | Promoting synovial macrophage M2 polarization and improve the pathological changes of osteoarthritis | No relevant tests were performed | [90] |
| Mammals | Mice | SMSCs | None | Exosomes | TEM (a double-layer membrane structure and showed a tea-ware tray shape), DLS (30-100 nm), and WB (CD63^+^, HSP70^+^; Calnexin^-^) | 10 μL SMSC-Exo (100 μg/mL) | Intra-articular injection | Intra-articular injection of lentivirus of oe-NC, oe-MATN3, sh-NC, sh-MATN3, PBS (10 μL), and SMSC-Exo (10 μL), respectively. | Mice knee OA model (DMM) | Inhibited the release of inflammatory cytokines, ECM degradation and autophagy defects in chondrocytes by delivering MATN3 | No relevant tests were performed | [91] |
| Mammals | Mice | SMSCs | None | Exosomes | TEM (single membrane structure), DLS (100 nm), WB (CD81^+^ and TSG101^+^) | Not clearly described in the article | Co-culture | Exosomes added in IL-1β-exposed chondrocytes | *In vitro* only | Suppressing apoptosis, inflammation, and ECM degradation of osteoarthritic cartilage cells by targeting NRP1 to inactivate the PI3K/Akt pathway | No relevant tests were performed | [92] |
| Mammals | Mice | Vascular endothelial cell | None | Exosomes | TEM (the morphological features of isolated EC-Exos, and the observed 60-110 nm bilayer disc-like vesicles had characteristics consistent with exosomes), NTA (90-180 nm), WB (Alix^+^, CD81^+^, CD9^+^ and Tsg101^+^) | 100 μg | Tail vein injection | Injection of EC-Exos (100 μg each) through the tail vein once per week. | Mice knee OA model (DMM) | Reduced the antioxidant capacity of chondrocytes, inhibited autophagy, and exacerbated knee degeneration in mice with DMM-induced osteoarthritis | No relevant tests were performed | [93] |
| Mammals | Mice | Macrophages | None | Extracellular Vesicles | TEM (129.2 ± 9.2 nm for EVs from non-stimulated macrophages and 148.1 ± 24.7 nm from LPS-stimulated macrophages), WB (TSG101^+^, Alix^+^, CD9^+^, and annexin V^+^) | 10 μl (100 μg/ml) | Intra-articular injection | The inflammatory macrophage EVs were intraarticularly administrated twice a week for 3 weeks at a protein concentration of 100 μg/ml | Mice knee OA model (collagenase-inducedand ACLT) | EVs secreted from inflammatory macrophages initiate the noncanonical pyroptosis of chondrocytes, thus leading to cartilage catabolism and the development of OA. | No relevant tests were performed | [94] |
| Mammals | Rat | BMSCs | None | Exosomes | TEM (hollow spherical microvesicles), WB (CD63^+^, CD81^+^ and CD9^+^) | 100 μl, 10^11^particles/ml | Intra-articular injection | The corresponding solution was injected into the joint cavity of each group (100 μl, 10^11^particles/ml) | Rat knee OA model (MCLT+MMT) | Negatively regulate Sp1 and promote the polarization of M2 macrophages, thus promoting the repair of cartilage and proliferation of chondrocytes | No relevant tests were performed | [95] |
| Mammals | Rat | BMSCs | None | Exosomes | TEM (typical circular or elliptical structure, and the exosomes had a particle diameter of 30-120 nm), WB (CD63^+^ and CD9^+^) | 20 g | None | IL-1 induced chondrocytes were treated with 20 g exosomes each time | *In vitro* only | Inhibited CDH11 in chondrocytes and further block the Wnt/β-catenin pathway, thereby protecting chondrocytes from OA injury | No relevant tests were performed | [97] |
| Mammals | Rat | BMSCs | None | Exosomes | DLS (mean size was 153 nm), TEM (exhibited an ova shape), WB (Flotillin-1^+^, TSG101^+^, and CD63^+^; Calnexin^-^) | 40 µg/100 µl) | Intra-articular injection | Intraarticular injection of BMSC-derived exosomes (40 µg/100 µl)) was performed once a week | Rat knee OA model (injection with 8% sodium iodoacetate) | Reduced OARSI scores and joint pain in the OA rats | No relevant tests were performed | [98] |
| Mammals | Rat | BMSCs | None | Exosomes | TEM (typical cup-like morphology), NTA (average vesicle diameter was about 140 nm), WB (CD63^+^, CD81^+^ and CD9^+^) | 10 µl (10^10^ particles/ml) | Intra-articular injection | At 4 weeks post-operation, rats were injected intra-articularly with 10 µl of BMSC-derived exosomes (10^10^ particles/ml) for 3 days for 4 weeks. | Rat knee OA model (induced using the modified Hulth technique) | Reduce the infiltration of synovial inflammatory cells and the damage of articular cartilage, thereby delaying the progression of OA | No relevant tests were performed | [99] |
| Mammals | Rat | BMSCs | None | Exosomes | TEM (40–100 nm, a round or tea-shaped morphology), WB (CD9^+^, TSG101^+^ and CD63^+^) | Not clearly described in the article | Intra-articular injection | MSC-derived exosomes, the exosomes carrying miR-9-5p mimics, miR-9-5p inhibitors, or their negative controls,  or miR-9-5p-embedded liposomes into the joint cavity of the left hind limb ankle joint. | Rat knee OA model (anterior cruciate ligament (ACL)/medial collateral ligament (MCL) transection) | Targeting SDC1 to lower inflammatory factor levels and reduce oxidative stress damage can alleviate osteoarthritis. | No relevant tests were performed | [100] |
| Mammals | Rat | BMSCs | Novel photo crosslinking spherical GelMA-encapsulated cartilage affinity WYRGRL (W) peptide-modified engineered Exo | Exosomes | SEM (round-shaped, the modification of the targeted peptide increased to ~107.4nm compared with that of unmodified exosomes in ~83nm), WB (TSG101^+^, CD81^+^, and CD9^+^; β-actin^-^) | 10 µL | Injecting hydrogels (DMM+Exo@GelMA, DMM+Exo-L@GelMA, and DMM+W-Exo-L@GelMA) | On the third day after the surgery, 10 µL corresponding solution was injected into the joint cavity, respectively, every two weeks for 8 weeks. | Mice knee OA model (DMM) | It has a strong chondrocyte targeting effect and can reduce chondropathy and subchondral bone loss in mouse models of OA | No relevant tests were performed | [101] |
| Mammals | Rat | BMSCs | None | Exosomes | TEM (a round or elliptical morphology with complete cell membrane structure), NTA (mainly around 100 nm), WB (CD9^+^ and TSG101^+^) | 15 μl | Injected into the defect site and rapidly crosslinked in situ under UV  irradiation/Hydrogels | A mixture of around 15 μl GMOCS-Exos was injected into the defect site and rapidly crosslinked in situ under UV irradiation. | Rat osteochondral defect model (d= 2.0 mm) | Alleviated the damage of IL-1 to chondrocytes, enhanced the synthesis of ECM in chondrocytes, promoted chondrogenesis and exos could be released continuously for 14 days | No relevant tests were performed | [102] |
| Mammals | Rat | BMSCs | None | Exosomes | TEM (spherical microvesicles), NTA (average diameter 80 nm), WB (HSP70+, TSG101^+^ and CD9^+^) | 100 μg | Injecting with hydrogels | The AD/CS/RSF/EXO were injected into the defect | Rat osteochondral Defect Model (d = 2 mm and h = 1 mm) | Accelerated the regeneration and extracellular matrix remodeling of a cartilage defect of the patellar groove in rats | At the beginning period of adhesion, the strength of AD/CS/RSF hydrogel reached to 121.7 ± 12.3 kPa in 50% humidity environment at 26 °C | [103] |
| Mammals | Rat | Synovial fibroblasts (SFCs） | None | Exosomes | TEM (generally round morphology), NTA (100±10nm), WB (CD63^+^ and CD81^+^) | 40 μl of 500 μg/ml | Intra-articular injection | At 4 weeks post-surgery, intra-articularly injected with 40μl of 500 μg/ml SFC-miRNA-126-3p-Exos or SFC-control-Exos once per week. | Rat knee OA model (ACLT+MMx) | Promoted chondrocyte proliferation, inhibited apoptosis and inflammation, and saved cartilage degeneration | No relevant tests were performed | [104] |
| Mammals | Rat | Primary  chondrocytes | None | Exosomes | TEM (a typical hollow, spherical-like morphology), NTA (40-140nm, with an average value of ~90 nm), WB (CD63^+^, CD9^+^, and CD81^+^) | 200 μl | Intra-articular injection/Hydrogels | At 4 weeks post-operation, once every 2 weeks for 3 times. | Rat knee OA model (DMM) | Regulating proliferation, migration, and differentiation of chondrocytes, the polarization of macrophages, and reduced excessive inflammatory response, so as to effectively prevent the destruction of articular cartilage | No relevant tests were performed | [105] |
| Mammals | Rat | L-cells | None | Exosomes | Electron microscopy, NTA, WB (Tsg101^+^) | 7 μl | Intra-articular injection | Liquid rat collagen type 1 gel containing exosomes was injected until the defect was full | Mice osteochondral Defect Model (0.78+/ 0.042mm wide and 1.79+/ 0.056mm deep) | Injecting WNT3A-loaded exosomes into the joints of mice consistently activated the WNT signaling pathway and improved the repair of osteochondral defects. | No relevant tests were performed | [108] |
| Mammals | Rat | Primary chondrocytes | Small extracellular vesicles (exos) are ligated with chondrocyte affinity peptide (CAP) using Sortase A and subsequently incubated with cholesterol-modified ASO-MMP13 to construct a chondrocyte-targeted drug delivery exo (CAP-exoASO). | Small extracellular vesicles | TEM (most particles appearing below 200 nm), NTA (most CAP-exoASOs were in the 50–150 nm diameter range), WB (CD9^+^ and CD81^+^) | 100 μL CAP-exoASO (100 μg) | Intra-articular injection/ Engineering modified technology | Intra-articular injections through the joint cavity once a week for 4 weeks | Rat knee OA model (ACLT) | CAP-exoASOs attenuated inflammation, promoted chondrocyte proliferation and collagen synthesis, and reduced apoptosis, thereby restoring joint homeostasis. | No relevant tests were performed | [109] |
| Mammals | Rat | Induced pluripotent stem cells (iPSCs) | Surface charge reversion, phosphatidylethanolamine (PPD) modification | Small extracellular vesicles | TEM (round spherical), Nanoflow cytometry (76.27 ± 5.01nm), WB (CD9^+^, CD63^+^, and TSG101^+^) | 10 μl (1× 10^10^ particles/ml) | Intra-articular injection | 10 μl (1× 10^10^ particles/ml) of sEVs , PPDsEVs or PBS was intraarticularly injected into the knee joint cavity of mice once every two weeks. | Mice knee OA model (ACLT) | Enhanced cartilage uptake, cartilage penetration, and joint retention capacities.increase the intraarticular bioavailability of sEVs for OA improvement. | No relevant tests were performed | [110] |
| Mammals | Rabbit | Chondrocytes（CC） | None | Exosomes | TEM (a cup-shaped or round-shaped form with a diameter of 30-200nm), WB (CD9+, CD63+, and CD81+), Fluorescence microscope images (CM Dil-labeled exosomes) | 30 μg exosomes per 100 μL injection | Subcutaneous injection | CC-Exos and BMSC-Exos (30g exosomes per 100L injection) were administered subsequently on a weekly basis | Subcutaneous non-chondrogenic sites in nude mice | Maintaining the stability of constructed cartilage, reduce the inward growth of blood vessels, and prevent cartilage hypertrophy. Anti-angiogenic properties, which help promote cartilage repair | No relevant tests were performed | [111] |
| Mammals | Rabbit | BMSCs | None | Exosomes | TEM (nearly round-shaped, 50 - 15 nm), WB (CD9^+^, CD63^+^, and CD81^+^) | 80 μg/mL | None | None | *In vitro* only | Effectively inhibitd the changes of IL-1β-induced mitochondrial membrane potential, cell survival rate, apoptosis, and mitochondria-mediated apoptosis by regulating p38, ERK, and Akt signaling pathways | No relevant tests were performed | [112] |
| Mammals | Rabbit | BMSCs | None | Exosomes | DLS (40 - 110 nm), TEM (hollow spherical microvesicles), WB (TSG101^+^, CD9^+^, and CD63^+^) | Not explicitly state | Implanted with 3D bioprinted scaffold | The left defect was not treated (Group I) or received a 3D printed ECM/GelMA scaffold implant (Group III), whereas the right defect received a 3D printed GelMA (Group II) or 3D printed ECM/GelMA/exosome scaffold (Group IV) implant. | Rabbitsosteochondral defect Model (4 mm in diameter and 4 mm in height) | The scaffold could effectively retain exosomes for 14 days in vitro and 7 days in vivo, and greatly promoted cartilage regeneration and subchondral bone regeneration, reversed mitochondrial dysfunction, and polarized synovial macrophages to the M2 phenotype. | No relevant tests were performed | [113] |
| Mammals | Rabbit | Serum | None | Exosomes | TEM (the mean diameter from 32-25 nm), DLS (the size distribution was observed to be 37.1 nm), | Not explicitly state | None | Incorporation of miR‐140 via Lipofectamine | *In vitro* only | Promoted the differentiation of BMSCs into chondrocytes | No relevant tests were performed | [114] |
| Mammals | Deer | Antler stem cells (ASCs) | None | Exosomes | TEM, NTA (displayed a classical cup- or sphere-shaped morphology with an average diameter of ~100 nm) | 10 μL (1×10^8^ exosomes) | Intra-articular injection | For exosome injection, 1×10^8^ ASC derived exosomes in 10 μL PBS were locally injected into the articular cavity after 1 week of ACLT surgery with a frequency of once a week for 8 times. | Mice knee OA model (ACLT) | Restored grip strength levels, alleviated bone erosion and cartilage degradation, promoted cell proliferation, and reduced cell senescence | No relevant tests were performed | [118] |
| Mammals | Deer | ASCs | None | Exosomes | TEM, NTA (50–100 nm), WB (Alix+, CD63+, and TSG101+, and GM130-) | 200 µg/mL | Hydrogels | ASC-Exos (200 µg/mL), or hADSC-Exos (200 µg/mL, positive control) or hydrogels mix with exosomes | Rat osteochondral defect model (2 mm in diameter, 1 mm in depth) | ASC-Exos enhanced the proliferation capacity of chondrocytes and the proliferation and migration capacity of bone marrow mesenchymal stem cells, and played a significant role in the repair of cartilage injury. | No relevant tests were performed | [119] |
| Mammals | Pig | Articular chondrocytes | None | EVs | TEM (spheroid morphology), NTA (the concentration peak was at 139.5 nm) | None | None | None | *In vitro* only | Promoting proliferation, migration, chondrogenesis and differentiation of ADSCs (increased expression levels of COL2A1, ACAN and SOX-9) | No relevant tests were performed | [120] |
| Plants | Citrus limon | Juice | None | Exosomes | TEM (a uniform distribution in size (< 200 nm) and round shape), NTA (average size was around 153.8 nm) | 100 μg/mL | None | None | *In vitro* only | Promoted cell proliferation and down-regulated chondrogenesis | No relevant tests were performed | [131] |
| Plants | Solanum lycopersicum | Juice | None | Exosomes | TEM (a uniform distribution in size (< 200 nm) and round shape), NTA (average particle size was around 100.4 nm) | 100 μg/mL | None | None | *In vitro* only | Promoted differentiation of adipose stem cells into chondrocytes and upregulated the expression of genes associated with chondrogenesis, such as COL2, ACAN, and SOX9 | No relevant tests were performed | [131] |
|  |  |  |  |  |  |  |  |  |  |  |  |  |
| Bacteria | Proteus mirabilis | Bacteria | None | Outer-membrane vesicles (OMVs) | TEM, NTA | 5*10^8^ OMVs | Intra-articular injection to ankle joint | OMVs (5*10^8^) were injected into ankle joints. | Mice OA model (Collagen-Induced Arthritis (CIA)) | Down-regulating miR95-5p inhibits osteoclast formation and bone resorption to reduce CIA-induced bone loss | No relevant tests were performed | [136] |

**Table S2** Definition and usage recommendation of EVs, extracellular particles and other vesicles.

| **Term** | **Definition** | **Usage** |
| --- | --- | --- |
| Extracellular vesicles (EVs) | Particles that are released from cells, are delimited by a lipid bilayer, and cannot replicate on their own. | Recommended |
| Non-vesicular extracellular particles (NVEPs) | Multimolecular assemblies that are released from cells and do not have a lipid bilayer (non-vesicular extracellular particle fraction). | Recommended |
| Extracellular particles (EPs) | Umbrella term for all particles outside the cell, including EVs and NVEPs. | Recommended |
| EV mimetic | EV-like particles that are produced through direct artificial  manipulation. This term is preferred over ‘exosome-like vesicles’and similar terms that imply specific biogenesis-related properties. | Recommended |
| Artificial cell-derived vesicles (ACDVs) | EV mimetics that are produced in the laboratory under conditions of induced cell disruption, such as extrusion. | Recommended |
| Synthetic vesicles (SVs) | EV mimetics that are synthesized de novo from molecular components or made as hybrid entities, e.g., fusions between liposomes and native EVs. | Recommended |
| Small EVs (operational term) | Based on the diameter of the separated particles, small EVs are often described as <200 nm in diameter. However, measured diameter is related to the specific characterization method. | Recommended, but caution required |
| Large EVs (operational term) | Based on the diameter of the separated particles, large EVs are often described as >200 nm in diameter. However, measured diameter is related to the specific characterization method. | Recommended, but caution required |
| Other ‘operational terms’ | Physical characteristics: e.g., diameter: small extracellular vesicles (sEVs), large EVs (lEVs), density: low, medium, high (defined ranges). Biochemical composition: e.g., contains a specific (macro)molecule, such as a protein. Cellular origin and/or conditions under which EVs were generated: terms that highlight specific aspects of biogenesis such as molecular mechanisms, energy-dependence (or lack thereof) and functional state of the parent cell related to stress or death. | Recommended, but caution required |
| Exosome | Biogenesis-related term indicating origin from the endosomal system. Unless subcellular origin can be demonstrated, it is likely that a broad population of EVs is being studied, not exosomes specifically. Exosomes represent a subtype of small EVs: the diameter of intraluminal vesicles of endosomes is generally smaller than 200 nm. | Discouraged unless subcellular origin can be demonstrated |
| Ectosome | Biogenesis-related term indicating origin from the plasma membrane. Unless subcellular origin can be demonstrated it is likely that a broad population of EVs is being studied, not ectosomes specifically. Ectosomes can have a wide range of sizes, including sizes similar to those of exosomes. | Discouraged unless subcellular origin can be demonstrated |
| Microvesicle | Biogenesis-related term indicating origin from the plasma membrane. However, historically, the term has often been used to designate large EVs or all EVs, whatever their subcellular origin. This term can therefore lead to confusion. | Discouraged |
| Exosome-like vesicles | As ‘exosome’ is a biogenesis-related term indicating origin from the endosomal system, this and similar terms are discouraged for synthesized EV mimetics. | Discouraged |

Reproduced from ref. [22] with permission. Copyright 2024 The Authors. Journal of Extracellular Vesicles published by Wiley Periodicals, LLC on behalf of the International Society for Extracellular Vesicles.
